# Multistate Dihydroazulene‐Spiropyran Dyads: Path‐Dependent Switchings and Refinement of the “*Meta*‐rule” of Photoactivity

**DOI:** 10.1002/chem.202501061

**Published:** 2025-04-27

**Authors:** Siri Krogh Vegge, Jonas N. Lienert, Marie Erskov Krogh, Stine G. Stenspil, Mathias Dowds, Jonathan K. S. Hansen, Christoffer Warming, Caroline von Aufschnaiter, Viktor Bliksted Roug Pedersen, Josef Wachtveitl, Martina Cacciarini, Mogens Brøndsted Nielsen

**Affiliations:** ^1^ Department of Chemistry University of Copenhagen Universitetsparken 5 Copenhagen Ø DK‐2100 Denmark; ^2^ Institute of Physical and Theoretical Chemistry Goethe University Max‐von‐Laue‐Straße 7 60438 Frankfurt am Main Germany; ^3^ Department of Chemistry “U. Schiff” University of Florence Via della Lastruccia 3–13 Sesto F.no (FI) 50019 Italy

**Keywords:** charge transfer, conjugation, cross‐coupling, fluorescence, photochromism

## Abstract

Multistate switches are interesting systems for a plethora of potential applications, such as for data storage involving many different states or for logic operations characterized by specific outputs. The main challenge is to achieve a precise control of accessibility to a specific state via a given sequence of multiple stimuli. Here, we have connected dihydroazulene (DHA) and spiropyran (SP) photoswitches in dyads to elucidate differences in optical and switching properties between *ortho‐, meta‐*, and *para‐*phenylene‐bridged dyads. Dyads were prepared by Suzuki and Sonogashira coupling reactions and photoisomerizations studied in detail by stationary and ultrafast spectroscopies. Moreover, the kinetics of thermal back‐reactions of meta‐stable states were studied. The results show path‐dependent switchings of the dyads using light in combination with other stimuli (acid/base/heat), allowing access to eight distinct states. The accessibility to some specific states via only one sequence of external stimuli provides an additional degree of data storage—information is not only stored as the state itself but also as the unique sequence of stimuli required to reach this state. By changing the bridging unit between the photoswitches, various properties (outputs) were finely tuned such as absorption and fluorescence behaviors, lifetime of meta‐stable state, and photoisomerization dynamics.

## Graphical abstract

Dihydroazulene‐spiropyran (DHA‐SP) photochromic dyads were prepared and investigated by stationary and ultrafast spectroscopies. By comparing the overall properties, including dynamics of photoswitching, to those of monomeric units, the dyads satisfy the so‐called “*meta*‐rule”. The dyads can reach eight different states, some only by a specific sequence of stimuli, here represented by a finite‐state‐machine diagram—this path‐dependent switching adds an additional degree of data storage.

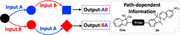

## 1 Introduction

Multiphotochromic systems consisting of several molecular switches are attractive candidates for advanced optical devices and functional smart materials, and they represent an emerging approach to achieve a spatiotemporal control of complex applications by light.^[^ ^1^ ^]^

**Figure 1:**
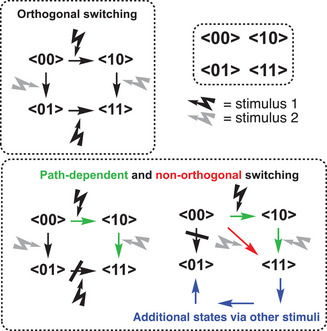
Conceptual illustration of switching pathways for bi‐photochromic system consisting of two molecular photoswitches that each can exist in two states (0 and 1).

While a singular molecular switch is typically a binary system, in which the presence of an external stimulus biases the switch toward the *on‐* or the *off‐*state, incorporation of multiple stimuli‐responsive subunits within a single molecule is a key tool to access multiple storage and nondestructive readout capacity, and it allows exploration of new properties. Such multimode‐photochromic structures can behave as complex logic devices depending on how they interact. If two units each represented by binary numbers (0 and 1) can be addressed independently by light of different wavelengths, orthogonal photoswitching^[^ ^1^, ^2^ ^]^ is obtained via two sequential routes as illustrated in Figure 1.

Instead, if the initial state <00> can only be transformed to the final state <11> via one route, we shall term it path‐dependent switching. Here having reached the <11> state contains information about the pathway and hence additional storage information; the switching events must have proceeded via the <10> state and not via the <01> state. This may result from loss of switching ability of one unit when the other has been switched, and it hence relates to the interaction between the units. Alternatively, it may be possible to only selectively switch one of the two units via one pathway, but not via the other (nonorthogonality). This implies that one intermediate state, e.g. <01>, cannot be reached at all or requires other stimuli than light as illustrated by the blue arrows in Figure 1.

**Figure 2:**
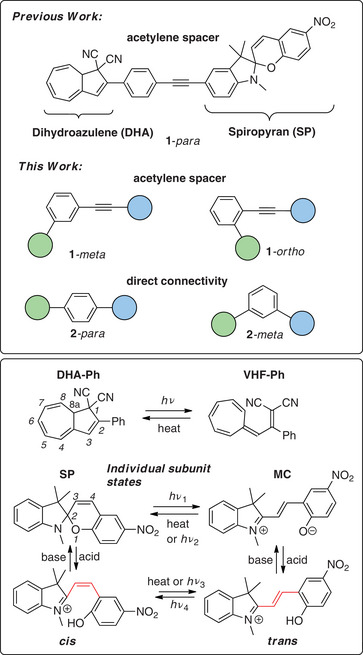
Top: Previously reported **1** *‐para* dyad and newly designed and investigated **1**‐*meta*, **1**‐*ortho*, **2** *‐para* and **2** *‐meta* dyads. Bottom: Individual switching states of dihydroazulene/vinylheptafulvene (DHA‐Ph/VHF‐Ph) and spiropyran/merocyanine (SP/MC) together with the protonated merocyanines as *cis* or *trans* isomers.

As an example of the latter system, we have recently reported a *para*‐connected photochromic dyad, **1**‐*para*, comprised of a dihydroazulene (DHA) and a spiropyran (SP) unit linearly conjugated with an acetylenic‐spacer (Figure 2, top).^[^ ^3^ ^]^ For dyad **1**‐*para*, the SP unit can be addressed by not only light stimuli, but also by acid, and the photoisomerized units all behaved as thermoswitches (thermal back‐conversions). The system exhibited eight distinct species. Two states derive from the light/heat‐induced isomerization between DHA and vinylheptafulvene (VHF),^[^ ^4^ ^]^ and four states from the light‐ or pH‐induced transformation of SP into merocyanine (MC) or into the corresponding *cis*‐or *trans*‐protonated MCs (Figure 2).^[^ ^5^ ^]^ The eight possible outputs were accessible by specific input sequences, that is, path‐dependent stimuli with light/heat and acid/base.^[^ ^6^ ^]^ In addition, dyad **1**‐*para* showed unexpected fluorescence solvatochromism, possibly originating from an intramolecular charge‐transfer state (ICT), not related to the individual units. Our experimental work on this DHA‐SP dyad was recently supported by a detailed computational study on the system by Deveaux et al.^[^ ^7^ ^]^

Interestingly, the photoswitching ability of both the DHA and SP units in **1**‐*para* seemingly violates the so‐called “*meta*‐rule”^[^ ^8^ ^]^ of photoactivity, which states that photoisomerization requires weak coupling (*meta*‐configuration) between the units. Such an empirical rule was, for example, established for DHA‐azobenzene dyads,^[^ ^9^ ^]^ in part for DHA‐norbornadiene dyads^[^ ^10^ ^]^, and for azobenzene multimers.^[^ ^11^ ^]^ It was, however, not required for azobenzene‐spiropyran dyads, for which orthogonal photoswitching was observed for *meta*‐ and *para*‐connectivity.^[^ ^2b^ ^]^ In its simplest form, the *meta*‐rule basically only takes into account the ability to undergo photoisomerization. As we shall show in this work, if the dynamics of photoswitching is integrated in the rule (by comparing the dynamics within dyads to that of individual monomer units), which is a reasonable demand, the rule holds for DHA‐SP dyads.

Herein we study more systematically the influence of connectivity patterns in DHA‐SP dyads, including the less explored *ortho‐*connectivity, not only in regard to optical properties and photoswitching, but also in regard to photoisomerization dynamics. We report the synthesis and properties of the regioisomers **1**‐*meta* and **1**‐*ortho* and the dyads **2**‐*par*a and **2**‐*meta*, missing the acetylenic unit within the spacer (Figure 2).

## 2 Results and Discussion

The syntheses of the two isomeric dyads connected with an acetylenic spacer at the *meta* or *ortho* positions relative to the 2‐phenyl ring on DHA (**1**‐*meta* and **1**‐*ortho*) were achieved following a copper‐free (i.e., no addition of copper salt) Sonogashira coupling (see Supporting Information, Scheme S1), in analogy with the protocol for the previously studied dyad **1**‐*para*,^[^ ^3^ ^]^ reacting an acetylenic spiropyran^[^ ^12^ ^]^ with the corresponding *meta*‐iodo DHA^[^ ^13^ ^]^ or *ortho*‐iodo DHA.^[^ ^14^ ^]^ As the DHA and SP precursors are chiral molecules used as racemates, **1**‐*meta* and **1**‐*ortho* were obtained as mixtures of diastereoisomers, inseparable by standard chromatography. In the ^1^H‐NMR spectrum at 500 MHz of **1**‐*ortho*, a splitting of some characteristic signals (H‐8a and H‐8 on the DHA unit and CH_3_ on the SP unit) was detected as shown in Supporting Information (Figure S6).

The synthesis of the two dyads **2**‐*para* and **2**‐*meta*, constituted by an SP moiety directly connected to the phenyl ring of a DHA unit at the *para* or *meta* position, was achieved via a Suzuki coupling of a spiropyran incorporating a boronic ester (Supporting Information, Scheme S2) and the corresponding *para*‐^[^ ^15^ ^]^ or *meta*‐iodo‐phenyl DHA, following the route depicted in Scheme S3. In both cases, azulene by‐products (formed by elimination of HCN) were isolated in up to 10% yield. Compounds **2**‐*meta* and **2**‐*para* were obtained as mixtures of indistinguishable diastereoisomers according to ^1^H‐NMR spectroscopic analysis (minor splitting of signals was detected only in the ^13^C‐NMR spectrum) and TLC. Their composition was analyzed by chiral HPLC before and after irradiation (see Supporting Information). The DHA‐SP chromatogram showed four distinguishable peaks, of which two, however, are overlapping, corresponding to the four different stereoisomers (two enantiomeric pairs). Chiral HPLC analysis after irradiation showed two new peaks and disappearance of the four original ones, indicating the presence of only two stereoisomers after photoconversion. This experiment thereby supports a selective ring‐opening of one switch (DHA to VHF or SP to MC) that results in the removal of one stereocenter and hence only the presence of two stereoisomers. From NMR and UV‐Vis spectroscopic studies, the irradiation at 415 nm had selectively resulted in DHA‐to‐VHF conversions (*vide infra*).

### 2.1 Spectroscopic Studies

**Figure 3:**
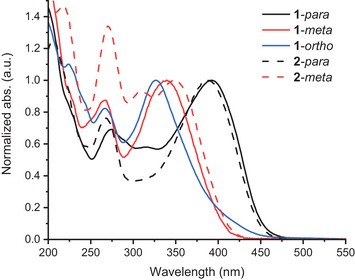
UV‐Vis absorption spectra in MeCN of the novel dyads together with reference compound **1**‐*para*.

The UV‐Vis absorption spectra of compounds **1–2** in MeCN are shown in Figure 3. The **1**‐*ortho* derivative has the most blueshifted longest‐wavelength absorption band within the acetylenic linker series, with a maximum at 327 nm; there is, however, a weak absorption observed in the range 400–450 nm which could indicate a first transition, resembling that of the **1**‐*para* derivative. We speculate that the phenyl ring is twisted out of the plane of the DHA due to the steric bulk of the two moieties, which results in the first transition having lower oscillator strength and therefore low absorption.^[^ ^14^ ^]^ Isomer **1**‐*meta* has its longest‐wavelength absorption maximum at 339 nm, hence significantly blueshifted related to the reference compound **1**‐*para* (392 nm). In the series without an acetylenic bridge, a slight blueshift of both the first and second transition is observed for **2**‐*para* versus **1**‐*para*, associated to the DHA and SP absorptions, respectively, likely due to shortening of the conjugated system. For **2**‐*meta*, direct linking of DHA and SP resulted in the splitting of the lowest‐energy absorption band (see Supporting Information, Figure S98). A blueshifted absorption of the *meta* versus the *para* dyad is observed also in this series, with absorption maxima at 349 for **2**‐*meta* and at 387 nm for **2**‐*para*.

Next, an extended investigation of the ability of the dyads to undergo switching upon different stimuli, such as light, acid/base, and heat was performed.

### 2.2 Photoswitchings

Upon light irradiation, the DHA unit can be converted into VHF and the SP unit into MC as depicted in Figure 2. The parent **DHA‐Ph** has a characteristic absorption maximum at 353 nm (and onset around 420 nm) in MeCN and the **VHF‐Ph** isomer at 470 nm.^[^ ^16^ ^]^ The parent **SP** has a characteristic absorption maximum at 343 nm (and onset before 400 nm) in MeCN and the **MP** isomer at 567 nm.^[^ ^17^ ^]^

**Figure 4:**
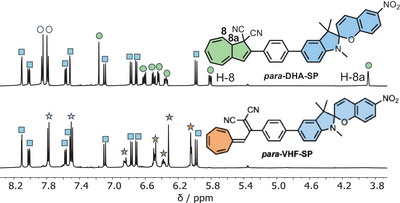
^1^H‐NMR spectra of **2**‐*para* in CD_3_CN before (top) and after irradiation at 415 nm (bottom), showing the disappearance of the typical DHA unit protons, H8a and H8. White symbols refer to phenylene protons, green symbols to DHA protons, blue symbols to SP protons, and orange symbols to VHF protons.

**Figure 5:**
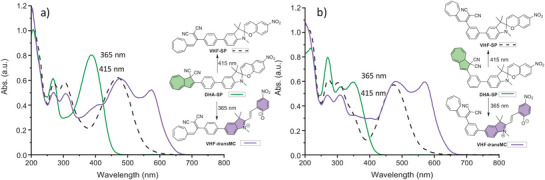
UV‐Vis absorption spectra of a) **2**‐*para* and b) **2**‐*meta* in MeCN at 25 °C: DHA‐SP state (green lines), VHF‐SP state (dashed lines, obtained by irradiation of DHA‐SP at 415 nm) and VHF‐MC state (purple lines, obtained by irradiation of DHA‐SP at 365 nm).

A selective light‐induced ring‐opening of the DHA to VHF was achieved for **2**‐*para* and **2**‐*meta* upon irradiation at 415 nm, leaving unaffected the SP unit, and forming only the VHF‐SP form. The conversion of compound **2**‐*para* was followed by both NMR (Figure 4) and UV‐Vis absorption (Figure 5, left) spectroscopies. For **2**‐*para*, the characteristic DHA absorption peak at *λ*_max_ 387 nm disappeared, and a new one at *λ*_max_ 468 nm appeared, characteristic for VHF (Figure 5, left, green solid line to black dash). Similarly, for compound **2**‐*meta*, the characteristic DHA absorption peak at *λ*_max_ 349 nm disappeared, and the characteristic VHF absorption peak at *λ*_max_ 475 nm appeared (Figure 5, right, green solid line to black dash). As the absorption maximum (349 nm) for **2**‐*meta* is very close to that of the parent **DHA‐Ph** (353 nm), the DHA and SP units in **2**‐*meta* seem not to be strongly coupled. In terms of onsets of the parent individual **DHA‐Ph** and **SP** (Figure 2), only the DHA unit has an absorption onset beyond 415 nm, which is in line with the selective isomerization of this unit of the dyad at this wavelength. For **2**‐*para*, the redshifted “DHA absorption” at 387 nm signals the presence of a larger chromophoric unit than just the DHA itself, but the DHA still exhibits its individual photoactivity. The selective DHA‐to‐VHF transformation induced by light at 415 nm was also confirmed by ^1^H‐ NMR spectroscopic studies, which showed the disappearance of the characteristic H‐8a and H‐8 signals on the DHA unit (see Figure 4 for compound **2**‐*para*).

Conversely, irradiation at 365 nm induced both the transformation of the DHA unit into VHF and the SP unit into MC, forming the VHF‐MC form of both dyads. For compound **2**‐*para*, the absorption peak at *λ*_max_ 387 nm disappeared, and together with an absorption at *λ*_max_ 474 nm of the VHF form, a peak at 573 nm appeared indicating the presence of the MC form (Figure 5, left, green line to purple). Analogously for compound **2**‐*meta*, the absorption peak at *λ*_max_ 349 nm disappeared, and together with an absorption at *λ*_max_ 481 nm of the VHF form, a MC absorption peak at *λ*_max_ 570 nm appeared (Figure 5, right, green line to purple).

**Figure 6:**
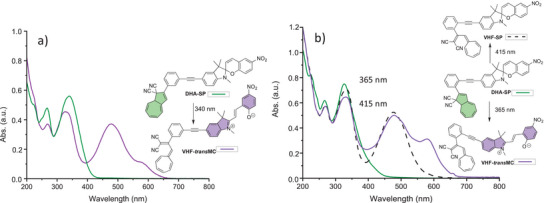
UV‐Vis absorption spectra of a) **1**‐*meta* and b) **1**‐*ortho* in MeCN at 25 °C: DHA‐SP (green lines), VHF‐SP (dashed line, obtained by irradiation of DHA‐SP at 415 nm for **1**‐*ortho*) and VHF‐MC (purple lines, obtained by irradiation of DHA‐SP at 340 nm for **1**‐*meta* and at 365 nm for **1**‐*ortho*).

For **1**‐*ortho*, irradiation at 415 nm gave rise to the formation of new peaks in the VHF absorption region (477 nm) (and none in the more redshifted MC region), but only a minimum decrease of the peak at 327 nm characteristic of the DHA‐SP species (Figure 6, right). Yet, the strong VHF absorption at 477 nm convincingly indicates DHA‐to‐VHF conversion, and chiral HPLC analysis (SI, Figure S57) shows the change from four to two stereoisomers (SP enantiomers) upon irradiation, in support of selective DHA‐SP to VHF‐SP conversion also with the *ortho* connectivity. Irradiation at 365 nm of **1**‐*ortho* or 340 nm for **1**‐*meta* did not significantly decrease the absorbance at 327 nm (**1**‐*ortho*) or 339 nm (**1**‐*meta*) but showed the formation of two new peaks at 479 nm (VHF form) and 579 nm (MC form) for **1**‐*ortho* and at 478 nm and 575 nm for **1**‐*meta* (Figure 6), corresponding to formation of VHF‐MC isomers.

### 2.3 Thermal Back‐Reactions

All five dyads gave stepwise thermal back‐reactions; that is, from VHF‐MC to VHF‐SP and then from VHF‐SP to DHA‐SP. These conversions were followed by UV‐Vis absorption spectroscopy and evaluated by mono‐exponential fitting of absorbance changes. The results are reported in Table 1, together with the absorption maxima in MeCN for each form.

**Table 1:** Longest‐wavelength absorption maxima (*λ*_max_, nm) and corresponding extinction coefficients (ε, 10^3^ M^−1^ cm^−1^) in MeCN, MC and VHF half‐lives (*t*_1/2_, minutes) at 25 °C in MeCN.

	1‐*para* ^[^ ^a^ ^]^	1‐*meta*	1‐*ortho*	2‐*para*	2‐*meta*
DHA‐SP form					
*λ*_max_ (*ε*)	392 (38.8)	339 (40.8)	327 (36.9)	387 (40.5)	349 (28.6)
VHF‐SP form					
*λ*_max_ (*ε*)	473 (27.0)	n.d.	477 (25.7)	468 (31.3)	475 (27.1)
VHF‐MC form					
*λ*_max_ (*ε)*	580 (21.5), 477 (26.4)	575^[^ ^d^ ^]^ (6.34), 478^[^ ^d^ ^]^ (27.1)	579 (11.6), 479 (23.9)	573 (25.2), 474 (31.1)	570 (27.1), 481 (29.0)
*t*_1/2‐MC_	0.37	0.68	0.53	1.66	1.80
*t*_1/2‐VHF_	139	155	1508^[^ ^b^ ^]^ 122^[^ ^c^ ^]^	182	328

^[a]^ Ref. [3].

^[b]^ Not fully completed.

^[c]^ At 45 °C.

^[d]^ Irradiation at 340 nm.

The MC half‐life *t*_1/2_ at 25 °C was in general very short and only slightly affected by the structure, spanning from 0.37 minutes (**1**‐*para*) to 0.53 minutes (**1**‐*ortho*) and then to 0.68 minutes (**1**‐*meta*). Removal of the acetylenic unit of the bridge induced a 4‐fold increase in the MC half‐life of compound **2**‐*para* in respect to **1**‐*para* (1.66 minutes versus 0.37 minutes) and a 3‐fold increase for **2**‐*meta* in comparison to **1**‐*meta* (1.80 minutes versus 0.68 minutes). As for the VHF half‐life, that of compound **1**‐*ortho* (1508 minutes) differed by one order of magnitude from those of **1**‐*para* (139 minutes) and **1**‐*meta* (155 minutes). These results confirm the trend observed for VHF‐Ph with an *ortho* substituent at the phenyl unit; a 1,2 connectivity elongates the VHF half‐life.^[^ ^14^ ^]^ We assume that this connectivity renders the s‐*cis* conformation of the VHF, required for ring closure, more difficult to obtain.

Removal of the acetylenic unit of the linker amplified the difference in the VHF half‐life between the isomeric couple **2**‐*para* and **2**‐*meta* (182 minutes versus 328 minutes), in comparison to acetylenic compounds **1**‐*para* and **1**‐*meta* (139 minutes versus 155 minutes).

Spectroscopic and isomerization studies were performed also for some of the other spiropyrans isolated (boronic ester derivative and azulene by‐products; see Supporting Information). The data reveal a slight enhancement of the half‐life when the MC group is connected to an azulene ring in comparison to a DHA unit.

### 2.4 Acid‐Promoted Switchings

As alternative stimulus to light, the SP/MC isomerization can be promoted also by pH changes, as depicted in Figure 2. Treatment with a strong acid, such as trifluoroacetic acid (TFA), causes protonation of the SP and ring opening into the protonated MC, initially formed in the *cis* configuration. It is possible to switch between the *cis/trans* configurations by irradiation at different wavelengths and/or by heating.^[^ ^5b^ ^]^ Addition of base to the *cis* isomer can regenerate the original SP, while addition of base to the *trans* isomer gives rise to the MC (Figure 2).

**Figure 7:**
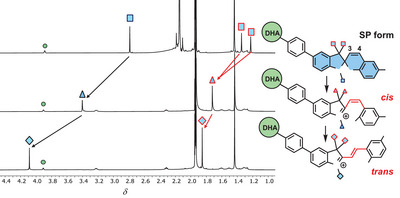
Aliphatic region of ^1^H‐NMR spectra (500 MHz, CD_3_DN) of **2**‐*para*, before (top) and after (middle) addition of TFA, and after heating at 50 °C for 42 hours (bottom). The selected region reveals switching of the SP unit to protonated MC as a *cis* isomer followed by its thermal conversion into a *trans* isomer.

The switching behavior of dyads **2**‐*meta* and **2**‐*para* upon addition of TFA and then heating at 50 °C was investigated by one‐ and 2D NMR spectroscopy experiments in CD_3_CN. The selective switching of the SP unit into a protonated merocyanine bearing a *cis* configuration and then the *cis*‐to‐*trans* isomerization was verified. The DHA unit was unaffected by the acidic treatment. Expansions of the aliphatic region of the ^1^H‐NMR spectra of **2**‐*para* at 500 MHz before (top spectrum, SP form) and after (middle spectrum, *cis* isomer) addition of TFA and then after heating at 50 °C (bottom spectrum, *trans* isomer) for 42 hours are shown in Figure 7. These spectra are informative of the protonation step and ring opening of the SP. In the ^1^H‐NMR spectrum of **2**‐*para*, the singlet at 2.79 ppm (square, black frame) can be assigned to the methyl group on the nitrogen, and the two singlets at 1.21 and 1.34 ppm (squares, red frame) can be assigned to the two other methyl groups on the *sp* ^3^ carbon in the SP moiety. When TFA is added to the sample, the SP ring‐opens to the protonated MC, and the stereogenic spiro center is lost. This is observed in the ^1^H‐NMR spectrum by the two methyl groups on the *sp* ^3^ carbon now appearing as one singlet at 1.72 ppm (middle spectrum, triangle, red frame). The singlet from the methyl group on the nitrogen atom is deshielded by 0.61 ppm after addition of TFA, as it is now connected to a positively charged nitrogen. After heating the sample at 50 °C for 42 hours, both the singlets were deshielded further by 0.69 ppm and by 0.13 pm, respectively. The characteristic DHA proton resonance at 3.9 ppm (H‐8a, see Figure 4 and Supporting Information for full assignments) is here unaffected by the stimuli.

**Figure 8:**
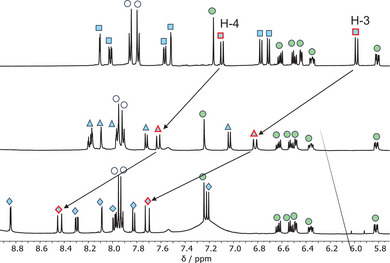
Aromatic region of ^1^H‐NMR spectra (500 MHz, CD_3_DN) of **2**‐*para*, before (top) and after (middle) addition of TFA, and after heating at 50 °C for 42 hours (bottom). Arrows highlight the olefinic protons. Green circles indicate DHA protons, white circles refer to phenylene protons, cyanic squares indicate SP protons (for SP numbering, see Figure 2), cyanic triangles indicate the alkene protons from *cis* protonated MC, and cyanic diamonds indicate the alkene protons from *trans* protonated MC. The broad signal around 7.2 ppm is assigned to exchangeable protons (phenol/TFA).

When comparing the aromatic region of the spectra (Figure 8), it transpires that the DHA unit is unaffected by the acid treatment and heating except for slight changes in chemical shifts (signals labelled by green circles). The olefinic signals of the SP unit, assigned by 1D and 2D NMR spectra, are evidenced in the stacked spectra and appear as doublets with a coupling constant of 10.4 Hz, typical of a *cis* double bond configuration. TFA addition and then heating induced a shift of both signals and a final coupling constant of 16.6 Hz, typical of a *trans* double bond configuration.

The different species detected by ^1^H‐NMR spectroscopy upon TFA addition were also studied for compound **2**‐*meta* (see Supporting information), and the compounds were characterized by UV‐Vis absorption spectroscopy.

### 2.5 Multiple Inputs: Path‐Dependent Switchings

**Figure 9:**
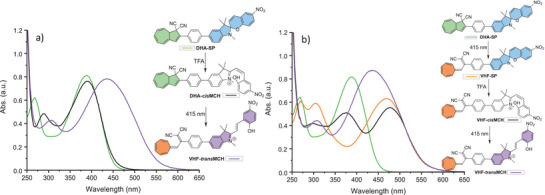
UV‐Vis absorption spectra of **2**‐*para* in MeCN. a) Before and after applying a stepwise, two‐stimuli sequence (TFA and 415 nm light). b) Before and after applying a stepwise, three‐stimuli sequence (415 nm light, TFA and 415 nm light).

To elucidate the switching topology (path‐dependent, orthogonal or nonorthogonal), different stimuli (inputs) were applied for **2**‐*para* and **2**‐*meta*. All the possible states given by the theoretical combination of DHA/VHF and SP/MC forms were accessible, i.e., eight different states, characterized by different outputs in terms of *λ*_max_, extinction coefficient, and half‐life of the specific meta‐stable isomer. For example, addition of TFA (SP‐to‐MC switching) followed by 415‐nm light irradiation (**2**‐*para*, Figure 9, left, acid+light) resulted in the formation of a VHF unit connected to a protonated merocyanine, with an absorption maximum at 450 nm (purple spectrum). Inverting the stimuli by first irradiating at 415 nm and then adding TFA provided a different spectrum (**2**‐*para*, Figure 9, right, light+acid, black spectrum) confirming a path‐dependent switching. Irradiating again at 415 nm, therefore using three stimuli, light+acid+light, gave the same spectrum obtained by the sequence acid+light, signaling a *cis*‐to‐*trans* isomerization of the protonated MC double bond (Figure 9, right, purple spectrum).

The combination of multiple inputs allowed us to study the influence of one photoswitch state on the thermal back‐reaction of a meta‐stable neighbor. The results are listed in Table S2 for MC‐to‐SP transformation, where also data for the SP boronic ester azulene derivatives are included, and Table S3 lists data for VHF‐to‐DHA conversion. In summary, the MC half‐life was not significantly affected by the state of the DHA/VHF system. Conversely, the VHF forms were more stabilized by *meta* versus *para* substitution, and by SP versus protonated MC (both for *cis* and *trans* isomers), providing longer half‐lives (see Supporting information).

### 2.6 Fluorescence Studies

**Figure 10:**
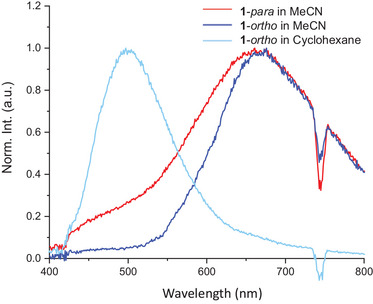
Emission spectra of **1**‐*para* in MeCN (red line) and **1**‐*ortho* in MeCN and cyclohexane (blue and light blue lines). Excitation wavelength (*λ*_exc_): 375 nm. The drop in intensity at ca. 740 nm is due to second‐order diffraction of the excitation source.

It was previously found that **1**‐*para* displayed emission from an ICT state in polar solvents such as MeCN. Herein, we want to investigate how this ICT appeared in the new derivatives. The **1**‐*meta* derivative did not display any emission, signaling the cross‐conjugation (reduced electronic coupling across the dyad) between the DHA and SP components—this lack of fluorescence is what one would expect from two separate DHA and SP photoswitches.^[^ ^18^ ^]^ Conversely, **1**‐*ortho* dyad displayed similar ICT characteristics as the **1**‐*para* compound (Figure 10). Excitation spectra confirmed the **1**‐*ortho* derivative as the emitting species (see Supporting Information, Figure S95). The emission of the locally excited state was measured in the apolar solvent cyclohexane and showed a more blueshifted emission at 497 nm. However, as the emission spectrum is not the mirror image of the absorption spectrum (see Figure S96), the emission likely originates from the transition with low absorption intensity in the 400–450 nm range.

The directly linked **2**‐*para* dyad also showed ICT emission, however with a less redshifted maximum at 637 nm and a larger emission yield of ∼1% compared to **1**‐*para* (0.37%) and **1**‐*ortho* (0.27%) (see, Supporting information, Figure S99). The emission of **2**‐*para* in cyclohexane (*λ*_em,max_ 473 nm) resembles closely the mirror image of the absorption, suggesting that the locally excited state is fully reached in this solvent (see Supporting Information, Figure S97).

### 2.7 Ultrafast Spectroscopy of the DHA‐to‐VHF Photoconversion

**Figure 11:**
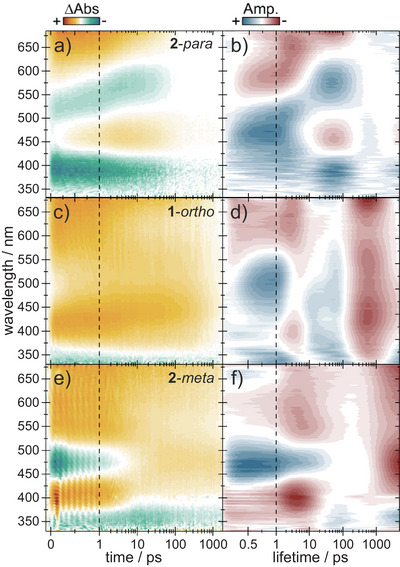
Transient absorption spectra of **2**‐*para* (a), **1**‐*ortho* (c) and **2**‐*meta* (e) along with the corresponding lifetime density maps (b, d, f) in CH_2_Cl_2_ after excitation at 400 nm. Positive signals in a, c and e refer to absorption of excited states (ESA) or photoproducts (PA) whereas negative signals account for stimulated emission (SE) or GSB. In b, d and f positive components describe the rise of negative or decay of positive signals and negative components indicate decay of negative or rise of positive signals.

According to the photostationary state analysis, transient absorption (TA) measurements with 400‐nm excitation wavelength should selectively trigger the DHA‐to‐VHF photoconversion; the results of such studies are shown in Figure 11 for **1**‐*ortho*, **2**‐*para*, and **2**‐*meta*. As there is no strong solvent effect (MeCN versus CH_2_Cl_2_) on steady‐state switchings of **2**‐*para* and **2**‐*meta* (for switchings in CH_2_Cl_2_, see Supporting Information; Figure S107), we focus in the following on studies in CH_2_Cl_2_. For **2**‐*para* (Figure 11a) in CH_2_Cl_2_, the ground‐state bleach (GSB) signal is observed mainly below 400 nm and lasts longer than the experimental observation time (> 2 ns). A positive signal accounting for excited‐state absorption (ESA) is located between 600 and 680 nm and shifts to higher wavelengths over time. The stimulated emission (SE) signal at 500 to 550 nm accompanies the ESA in this trend. After 0.5 ps, another excited‐state signal arises at 450 nm and decays together with all the other bands at around 100 ps.

To determine the underlying kinetics, the transient data was investigated by lifetime density analysis (LDA).^[^ ^19^ ^]^ A first major lifetime contribution with 0.9 ps corresponds to the decay of the DHA S_1_ → S_n_ absorption^[^ ^20^ ^]^ at 600 to 680 nm with a simultaneous rise of the 450 nm excited‐state signal (Figure 11b). The SE displays an ongoing redshift starting at 0.9 ps until 3 ps, indicating that both excited states decay radiatively. Therefore, the 450 nm ESA most likely originates from the previously mentioned ICT state present in the fluorescence spectra and described theoretically as “the presence of a low‐energy CT state” with “significant contribution to the nonlinear optical response” of the **1**‐*para* by Deveaux et al.^[^ ^7^ ^]^ All excited‐state bands decay with a lifetime distribution around 80 ps except for the 650 nm excited‐state signal, which has an additional early decay amplitude at 2 ps. The latter time matches the excited‐state decay and rise of photoproduct lifetime of the DHA monomer (see Supporting Information, Figure S103, A+B). Here, the positive remaining signal at 465 nm depicts the VHF‐SP steady‐state absorption, which possibly rises underneath the ICT band with this 2 ps. (According to previous work, s‐*cis*‐VHF is formed after the photoreaction which isomerizes on the ground‐state surface to the s‐*trans* form in ∼10 µs.)^[^ ^21^ ^]^ Remaining excited‐state population probably misses the conical intersection (CI) to the ground‐state surface and is trapped on the excited state for 80 ps. This reduces the switching quantum yield to 8% compared to the **DHA‐Ph** monomer (23%). The ICT state can be deemed unproductive toward VHF formation as well and further reduces the switching efficiency. QYs were measured according to Slavov et al. by excitation with a 405‐nm LED and observation of the signal rise at 465.^[^ ^22^ ^]^

In agreement with the observed Stokes shift, **2‐** *para* in MeCN displays similar dynamics to **2‐** *para* in CH_2_Cl_2_ with a detectable ICT state at 450 nm (see SI, Figure S100). As soon as the ICT state appears (<500 fs), the SE shifts toward longer wavelength and overlaps with the ESA. Steady‐state measurements detect this fluorescence peak at 637 nm (see Supporting Information, Figure S97). The transient spectra of **2**‐*para* in cyclohexane show no ICT absorption; the fluorescence therefore only originates from the DHA S_1_ state (see Supporting Information, Figure S100). The influence of the linker can be seen when comparing **1**‐*para* (see Supporting Information, Figure S101) and **2**‐*para*. SE and ICT absorption intensities are reduced in the presence of the alkynyl linker. As expected, the linker enhances the electronic decoupling from both moieties and therefore hinders the charge transfer from the SP to the DHA unit. This is in good agreement with the observed reduction in the fluorescence quantum yield.

As mentioned before, the absorption spectrum of **1**‐*ortho* (Figure 3) possibly displays two bands in the region of DHA S_0_ → S_1_ absorption of the *meta* and *para* compounds, one absorbing at 327 nm and another weak one absorbing between 400 and 450 nm. Isolating the DHA switching with a 327 nm TA experiment is difficult because of simultaneous SP photoconversion. Following Kasha's rule, TAS with excitation at 400 nm was carried out with expectation of the same DHA dynamics as for higher excitation (Figure 11c). Dyad **1**‐*ortho* shows the same DHA S_1_ → S_n_ absorption above 600 nm as the *para* compounds. Lifetime density analysis (Figure 11d) depicts two positive distributions at 650 nm and 400 nm, describing excited‐state decay around 2 ps. Additional decay amplitudes at 650 nm however indicate an excited‐state lifetime up to 1 ns. While the former ESA‐signal and lifetime compares well with the VHF formation dynamics observed for **2**‐*para* and DHA monomer, the latter can occur when excited‐state population misses the conical intersection toward the ground state. Another broad positive band centred at 425 nm is visible immediately after excitation. According to LDA, this signal has an early 500 fs rise and decays with 1 ns.

Taken the simultaneous 500 fs decay of an ESA band above 600 nm, a DHA‐S_1_ to dark ICT conversion in parallel to **2**‐*para* seems likely for **1**‐*ortho*. In agreement with steady‐state fluorescence data, the 425 nm band would therefore be attributed to the intramolecular CT state. Stimulated emission is not clearly visible, but a weak negative signal appears immediately after excitation at 500 nm which then shifts to higher wavelengths with the appearance of the ICT state after 0.5 ps. This band probably decays later around 575 nm and is reminiscent of the fluorescence signature of the *para* compounds. This behavior would explain the pronounced solvatochromism and reduced fluorescence quantum yield of **1**‐*ortho* in relation to **1**‐*para*.

We also explored the excited‐state dynamics of the *meta*‐connected dyad. Due to a blueshifted DHA absorption band of **2**‐*meta* compared to the *para* compounds, irradiation at 400 nm leads to excitation of both, the DHA and emerging VHF moieties. The TA spectra of the photoproduct monomer s‐*trans*‐VHF (see Supporting Information, Figure S103C‐D) show an ESA at 550 nm and a bleach at 475 nm that can be fitted with two exponential functions. The components were attributed to the S_2_ to S_1_ deexcitation and the S_1_ relaxation to the ground state by Schalk et al.^[^ ^23^ ^]^ Figure 11e of **2**‐*meta* clearly exhibits the same features. The lifetime density analysis reveals a biexponential recovery of the s*‐trans*‐VHF GSB with lifetimes of 0.5 and 10 ps. Remaining positive signals therefore belong to DHA excited states in **2**‐*meta*. Unlike the other dyads, **2**‐*meta* does not display any ICT signals. This is confirmed by steady‐state fluorescence as well as insignificant solvent dependency when switching from CH_2_Cl_2_ to MeCN (see Supporting Information, Figure S100). In agreement with the other dyads, two excited‐state signals at 400 nm and above 650 nm show different relaxation behavior. The hypsochromic ESA decays with a lifetime distribution of ∼5 ps and is productive toward VHF formation since its S_0_ → S_1_ absorption band at 465 nm rises at the same time. The longer‐lived ESA at 650 nm displays excited‐state splitting in agreement with the other dyads. An early decay at 5 ps together with the 400‐nm ESA is followed by a second decay at the end of the measurement window which exhibits a lifetime of 3 ns. This long lifetime does not participate in the formation of VHF since the intensity of the latter remains constant and therefore most likely originates from missing the CI between excited and ground state. Quantum efficiency measurements follow this trend; the yield of VHF formation for **2**‐*meta* (15%) is lower than for the **DHA‐Ph** monomer (23%) which does not show any excited‐state splitting (see Supporting Information, Figure S103). On the other hand, the yield is still higher than in **2**‐*para* which implies the ICT state and fluorescence as a more dominant loss channel than the excited‐state splitting.

The dynamics of the differently connected dyads present a refinement of the established *meta*‐rule.^[^ ^24^ ^]^ Here, the dynamics of **2**‐*meta* closely follows the findings for the monomer (see Supporting Information, Figure S103), whereas *ortho* and *para* compounds show different behavior.

### 2.8 Ultrafast Spectroscopy of the SP‐to‐MC Photoconversion

**Figure 12:**
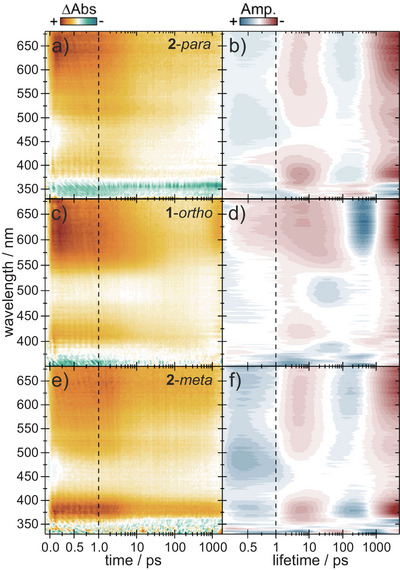
Transient absorption spectra of **2**‐*para* (a), **1**‐*ortho* (c) and **2**‐*meta* (E) along with the corresponding lifetime density maps (b, d, f) in CH_2_Cl_2_ after excitation from the 405 nm photostationary state with 360 nm.

We also investigated the influence of the connectivity pattern on the SP‐to‐MC switching. To isolate the SP dynamics, samples were illuminated according to the steady‐state analysis to reach the VHF photostationary state and then excited with 360 nm in the TAS experiment. All dyads show two long‐lived ESA, above 500 nm and around 400 nm. The GSB is located below 350 nm and remains throughout the experimentally accessible time window (Figure 12a,c,e). Lifetime density analysis (Figure 12b,d,f) yields three main lifetime distributions to describe the data. All dyads show a 500 fs rise time of the ESAs, indicating the participation of another short‐lived excited intermediate which spectrally lies outside of our detection system. The second lifetime distribution at ∼8 ps presents a splitting of the excited state since only part of the population decays. The remaining excited molecules change conformation between 300 and 700 ps indicated by the negative component in the LDM. Both excited‐state signals (above 500 nm and around 400 nm) last longer than the observation time of 2 ns. TA spectroscopy with two synchronized fs lasers helps to expand the timescale and reveals a decay of this excited state at ∼10 ns (see Supporting Information, Figure S104). Simultaneously, new absorption bands arise at 350 and 600 nm.

The NO_2_‐SP photoconversion has been studied extensively by theory^[^ ^25^ ^]^ and experiment.^[^ ^26^, ^27^ ^]^ It generally involves several triplet states whose nature is, however, unclear. Holm et al. have studied the dynamics of NO_2_‐SP with transient UV/VIS and IR experiments and found a 0.2‐ps fast decay of SP to MC due to an isoenergetic position of the SP S_1_ and the MC T_2_ state.^[^ ^28^ ^]^ After 0.5 – 2 ps, this state decays to the lowest MC triplet state, which shows characteristic absorption around 400 nm and above 500 nm (see SI, Figure S105). Here, we propose a similar scheme for the dyad systems. Decaying SP singlet excited state, which is known to display absorption below 400 nm and above 620 nm, is therefore attributed to the 500‐fs lifetime distribution.^[^ ^28^, ^29^ ^]^ The newly emerging bands are more reminiscent of the T_1_ than the T_2_ absorption of NO_2_‐SP. This implies a shift of the MC triplet states when incorporating the monomer into the dyad systems where the SP S_1_ now possibly lies isoenergetic to the MC T_1_ state. A fast T_2_ to T_1_ decay would be another plausible explanation for this lifetime structure. However, with the TA time resolution of 30 fs and the visible T_2_ 450 nm absorption of NO_2_‐SP, the T_2_ → T_1_ relaxation path seems unlikely. Further evidence for the fast SP to MC switching is provided by the 8‐ps lifetime since the decaying excited state is not accompanied by SP ground‐state recovery. Therefore, this lifetime can either be assigned as a cooling from hot MC T_1_^*^ to T_1_ or as a biexponential decay of MC T_1_ to one of the MC ground‐state isomers. The third lifetime distribution between 300 and 700 ps matches the previously reported 350‐ps time of an isomerization on the excited T_1_ surface from one MC isomer to another.^[^ ^29^ ^]^ In both works no spectral change was found for this isomerization.

To additionally confirm the theory of direct SP S_1_ to MC T_1_ relaxation, the decay of the triplet bands above 500 nm and around 400 nm was compared between the dyads and the NO_2_‐SP monomer. The previously mentioned 10‐ns lifetime is found in all measurements with only very slight variation, which makes the proposed mechanism very plausible. The lifetime as well as the emerging absorption patterns at 350 and 600 nm, match with the literature‐known MC excited‐state decay and ground‐state buildup.^[^ ^30^ ^]^ It is to note that the samples were not degassed in this work which could lead to shorter triplet excited state lifetimes. Due to a higher extinction coefficient of MC ground‐state isomers in contrast to excited‐state triplets, the resulting bands at 350 and 600 nm are higher in intensity. Comparing the last transient spectrum at few hundred ns with the steady‐state absorption reveals a blueshift of the 350‐nm band as well as a redshift of the 600‐nm signal (see Supporting Information, Figure S106). Hence, the photoreaction is still incomplete and reorganization and isomerization on the MC ground‐state surface continues into the µs time range. Unfortunately, no additional spectral changes were found for NO_2_‐SP after 30 µs by Görner and coworkers^[^ ^27^ ^]^ since MC ground‐state isomers are hard to spectrally distinguish.

In contrast to the DHA switching dynamics, the photochemistry of SP to MC does not show any significant dependence on the connectivity of the dyads. All spectral features and extracted lifetimes are very similar. Of course, delocalization of the π‐system covers the whole DHA backbone in **2**‐*para* and **1**‐*ortho* in contrast to **2**‐*meta*, whereas the photochromic part of the SP unit is separated from the large π‐system in all dyads. Therefore, connectivity matters when switching the DHA, but is less relevant for SP.

## 3 Conclusion

A selection of DHA‐SP dyads was prepared and their optical and switching properties deeply investigated. An increasing degree of electronic communication between the units was found for ethynyl(*ortho‐/meta‐/para‐*)phenylene‐bridged dyads in the sequence *ortho* (geometrical constraints) – *meta* (cross‐conjugation) – *para* (linear conjugation) and for *meta‐/para‐*phenylene‐bridged dyads in the sequence *meta* – *para* (with the *para* isomer exhibiting the most redshifted longest‐wavelength absorption maximum).

A wavelength‐selective DHA‐to‐VHF photoisomerization was possible, while SP‐to‐MC photoisomerization was always accompanied by DHA‐to‐VHF photoisomerization. Thermal MC‐to‐SP back‐reactions were in all cases very fast (minutes’ timescale), while the VHF‐to‐DHA back‐reactions were much slower (hours’ timescale), and, interestingly, one order of magnitude slower for the *ortho* isomer. The *ortho* and *para* isomers showed ICT emission, which was not displayed for *meta* isomers, in analogy to the absence of such emission of the individual parent units.

**Figure 13:**
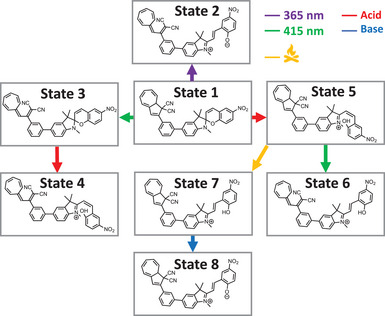
Graphical overview of the pathways to reach the eight isomerization states of **2**‐*meta* by stimuli starting from State 1. The color of the arrow indicates the stimulus/input used.

The dyads could also be stimulated by acid giving access to additional states, and Figure 13 gives an overview of the results for phenylene‐bridged dyad **2**‐*meta*. The eight theoretical states were all accessible, providing specific outputs, two of which were reached by two‐input path‐dependent routes (States 4 and 6).

**Figure 14:**
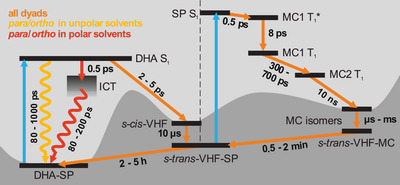
General reaction scheme of DHA‐SP dyads; when excited with a 400‐nm pulse to trigger the DHA‐to‐VHF photoconversion (left) and with 360 nm from the 400‐nm photostationary state to isolate the SP‐to‐MC switching.

In addition, ultrafast TA experiments have provided insight into the conversion dynamics of the dyads (Figure 14). Exciting the samples with 400‐nm light yields different results depending on the connectivity and solvent. Thus, *para‐* and *ortho*‐compounds display high solvatochromism, which originates from an ICT state. The fluorescence from the ICT and longevity of the DHA S_1_ state occur as loss channels affecting the photoswitchability from DHA to VHF. The investigated **2**‐*meta* dyad exhibits no fluorescence but a similar long‐lived DHA S_1_ state, which decreases the switching quantum yield compared to the DHA‐Ph monomer as well.

SP‐to‐MC photoswitching presents a more coherent picture between the dyads. The TA measurements at 360 nm were carried out on the 400‐nm photostationary state to exclude mixed dynamics between DHA and SP. Here we propose a fast relaxation from an excited SP singlet state to a MC T_1_ state, in contrast to NO_2_‐SP where a MC T_2_ state is involved. Over several excited‐state isomers, the MC triplet state of the dyads relaxes to the ground‐state surface within 10 ns. Several isomerizations occur on the µs timescale before the metastable VHF‐MC thermally relaxes back to VHF‐SP in minutes, and then to DHA‐SP within hours.

Overall, the so‐called *meta*‐rule is applicable to the DHA‐to‐VHF photoswitching because only **2**‐*meta* presents similar dynamics to the monomer, whereas for the SP‐to‐MC conversion connectivity is less important since all dyads show similar behaviour. Our results illustrate that the electronic communication between the DHA and the SP units can be finely tuned by the type of linker and by the *ortho*, *meta*, or *para* connectivity always maintaining reversible photoswitching ability, with the *ortho* and *para* connectivity exerting additional ICT emission – a novel property not exhibited by the individual units.

## Supporting Information

Synthesis protocols, characterization data, crystallographic data, photophysics data, and NMR spectra. The authors have cited additional references within the Supporting Information.^[^ ^31^, ^32^, ^33^, ^34^, ^35^, ^36^, ^37^, ^38^ ^]^

## Conflict of Interests

The authors declare no conflict of interest.

## Supporting information



Supporting Information

## Data Availability

The data that support the findings of this study are available from the corresponding author upon reasonable request.
