# Supplementary material for: Multistate Dihydroazulene‐Spiropyran Dyads: Path‐Dependent Switchings and Refinement of the “Meta‐rule” of Photoactivity
Source: Chemistry. 2025 Apr 27;31(29):e202501061. doi: 10.1002/chem.202501061 (PMC12099180; doi:10.1002/chem.202501061)
Supplement: Supplementary file 1 — Supporting Information [file CHEM-31-e202501061-s001.pdf]

SUPPORTING INFORMATION

Table of contents

- Synthesis and Characterization S02
- NMR spectra of **1**-*meta*, **1**-*ortho*, **9**, **6**, **2**-*meta*, **11**, **2**-*para*, **10** S09
- NMR spectra after single or multiple stimuli S28
- Chiral HPLC analysis S39
- UV-Vis absorption spectra and switching studies S42
- Influence of the status of one photoswitch on the TBR of the other S68
- Fluorescence spectra S69
- Ultrafast Spectroscopy S72
- Final Overview S77
- Crystal Data S78
- Bibliography S79

Synthesis and Characterization

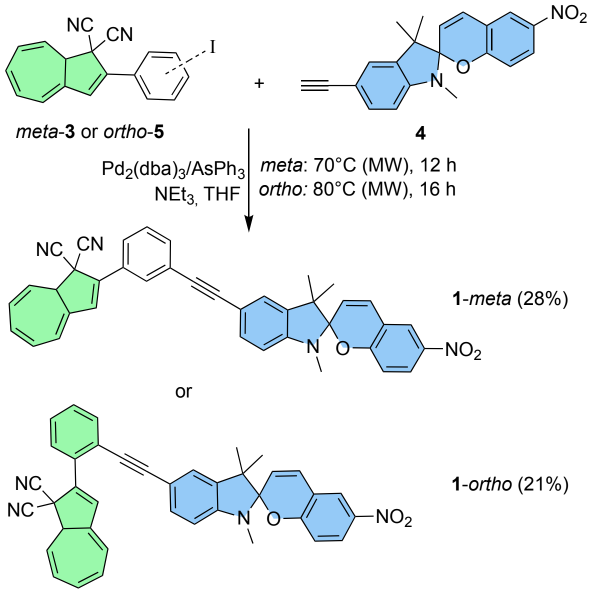

Scheme S1. Synthesis of compounds **1**-*meta* and **1**-*ortho*.

In analogy with the protocol for the previously studied dyad **1**-*para*,[3] the synthesis of **1**-*meta* from the *meta*-iodo DHA **3**[13] and the spiropyran-acetylene **4**[12] was carried out in THF at 70 °C (by microwave irradiation) with tris(dibenzylideneacetone)dipalladium(0) and triphenyl arsine as catalytic system in the presence of triethylamine. Compound **1**-*meta* was isolated in 28% yield. Analogously, **1**-*ortho* was obtained in 21% yield from the *ortho*-iodo DHA **5**[14] and the same spiropyran-acetylene **4** (Scheme S1).

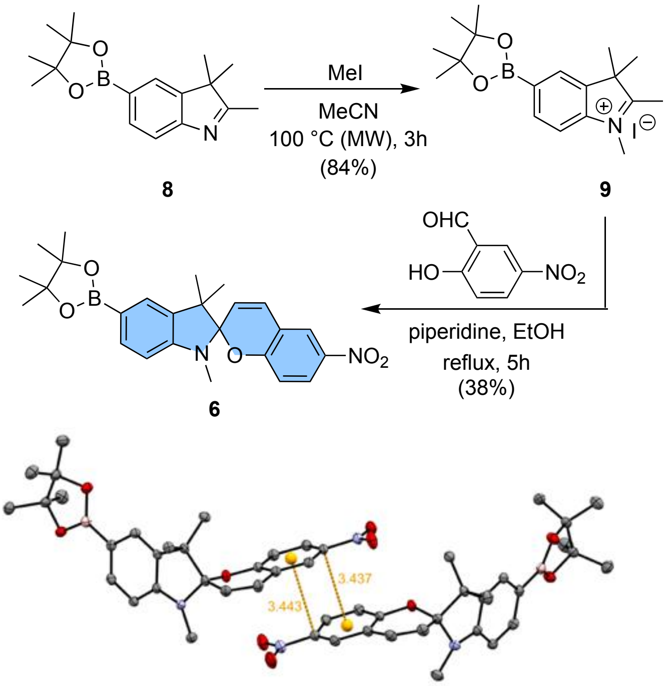

Scheme S2. Top: Synthesis of spiropyran **6**. Bottom: Molecular structure of **6** obtained by X-ray crystallography; two enantiomers shown. The C to centroid distance is highlighted.

The synthesis of the two dyads **2**-*para* and **2**-*meta*, constituted by an SP moiety directly connected to the phenyl ring of a DHA unit at the *para* or *meta* position, was achieved via a Suzuki coupling starting from the boronic ester spiropyran **6** (Scheme S2) and the known *para*- or *meta*-iodo-phenyl DHA **7**[15] and **3**, following the route depicted in Scheme S3.

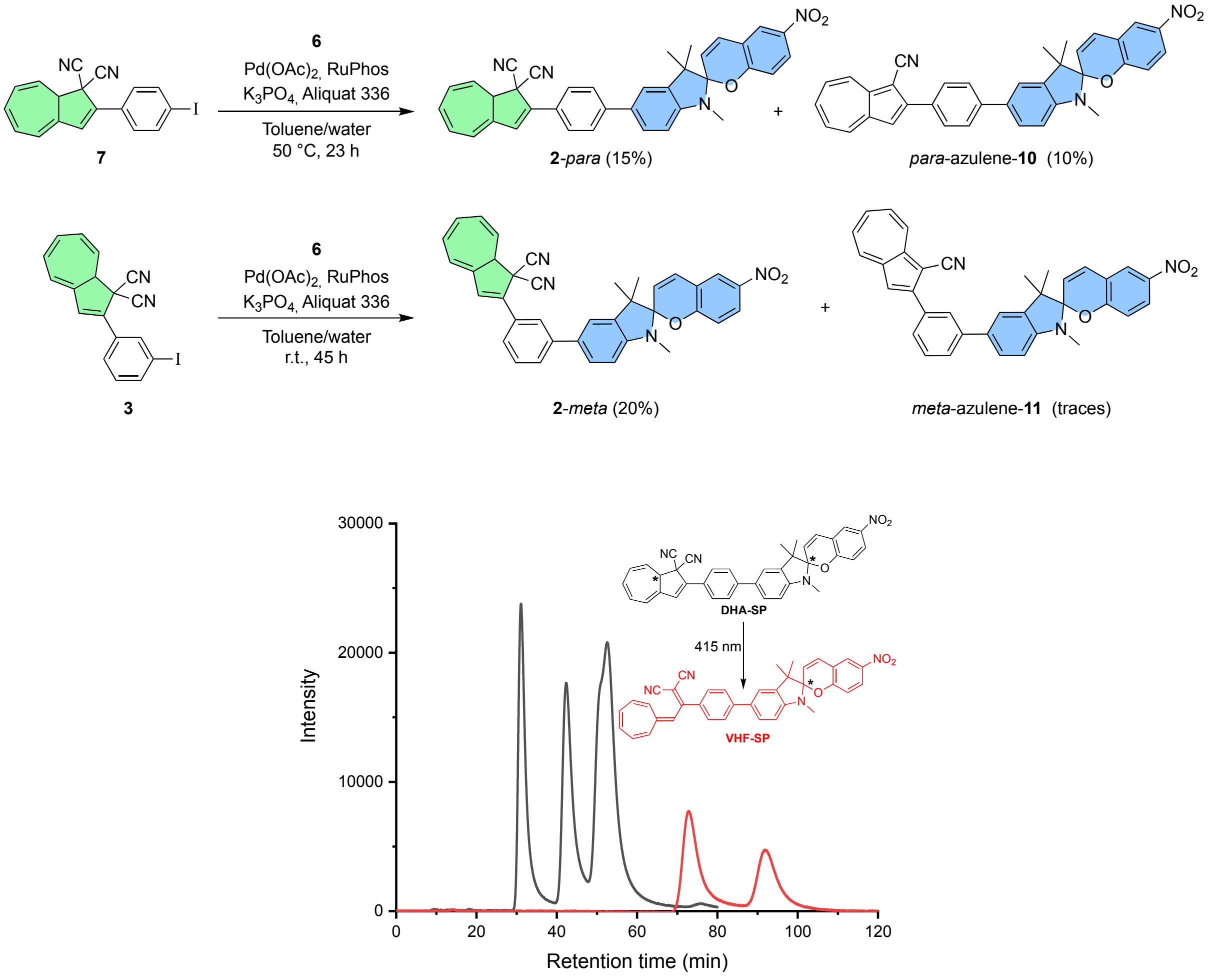

Scheme S3. Top: Synthesis of **2**-*para* and **2**-*meta* and formation of corresponding spiropyran-azulene by-products. Bottom: Chromatogram from chiral HPLC of **2**-*para* before and after irradiation at 415 nm, detected at 390 nm (black curves) and 468 nm (red curves), respectively. Eluent system: *i*-propanol/heptane.

The key intermediate boronic ester spiropyran **6** was then coupled with the *para*-iodo DHA derivative **7** using $Pd(OAc)_2$ and RuPhos, in the presence of Aliquat 336 and K_3PO_4 in toluene/water (10/1) at 50 °C for 23 h to form dyad **2**-*para*, isolated in 15% yield. The corresponding azulene by-product **10** (formed by elimination of HCN) was isolated in 10% yield. The coupling of the boronic ester spiropyran **6** with the *meta*-iodo DHA derivative **3** was performed under analogous conditions as for the *para*-iodo DHA derivative, using $Pd(OAc)_2$ and RuPhos, in the presence of Aliquat 336 and K_3PO_4 in toluene/water (10/1), at r.t. for 45 h to form dyad **2**-*meta* in 20% yield. Under these conditions, the spiropyran **6** was still present in the reaction mixture, while the *meta*-iodo DHA was fully consumed according to TLC inspection. Increasing the reaction temperature to 50 °C resulted in no formation of **2**-*meta* (reaction time: 23 h) or only 10% yield of **2**-*meta* (reaction time: 4 h). Changing the solvent mixture using toluene/ethanol/water (3/1.5/1) resulted in a faster reaction time (9 h), but

isolation of only 9% of **2**-*meta* and 23% of the corresponding azulene **11**, which was detected by TLC in every trials. Compounds **2**-*meta* and **2**-*para* were obtained as mixtures of indistinguishable diastereoisomes according to ^{1}H-NMR spectroscopic analysis (minor splitting of signals was detected only in the ^{13}C-NMR spectrum) and TLC. Their composition was analyzed by chiral HPLC before and after irradiation (see for example Figure S3 for compound **2**-*para*). The chromatogram showed four distinguishable peaks, of which two, however, are overlapping, corresponding to the four different steroisomers (two enantiomeric pairs). Chiral HPLC analysis after irradiation at 415 nm showed two new peaks and disappearance of the four original ones, indicating the presence of only two stereoisomers after photoconversion. This experiment supports a selective ring-opening of one switch (DHA to VHF or SP to MC) that results in the removal of one stereocenter and hence only the presence of two stereoisomers. From NMR and UV-Vis spectroscopic studies, the irradiation at 415 nm had selectively resulted in DHA-to-VHF conversions (*vide infra*).

The 3*H*-indole boronic ester **8**[31] was transformed into the corresponding indolium salt **9** by treatment with methyliodide in acetonitrile at 100 °C by microwave irradiation in 84% yield (on a 2-g scale, Scheme S2). Then the spiropyran ring was built by heating at reflux compound **9** together with 2-hydroxy-5-nitrobenzaldehyde and piperidine in absolute ethanol for 5 h. The boronic ester spiropyran **6** was obtained in 38% yield. Single crystals of **6** were grown by slow evaporation of a CD_3CN solution and subjected to X-ray crystallographic analysis, revealing π–π stacking of the two enantiomers (Scheme S2).[32]

General Methods

All glassware was shielded from light during the reaction and purification of light sensitive compounds. Melting points are uncorrected. All NMR spectra were recorded at 25.0 °C on a Bruker Avance 500 MHz instrument with a 5 mm CPDCH cryoprobe. NMR spectra were referenced against the residual solvent signal. HRMS were recorded with either a Bruker MicrOTOF-QII by the use of an ESI source or a Bruker Solarix ESI-MALDI-FT- ICR instrument equipped with a 7 T magnet (prior to the experiments, the instrument was calibrated using sodium trifluoroacetate (NaTFA) clusterions). MALDI mass spectra were obtained using dithranol as matrix. Microwave assisted syntheses were performed on a Biotage Initiator EXP EU microwave synthesiser (MW power: 400 W). HPLC analyses were performed using a Shimadzu CBM-20A Prominence instrument with UV detection using a 5-AmyCoat 4.6x250 mm column. The injection volume was 25 µL, and the flow rate was 1 mL/min. The concentration of the samples were ~ 0.2 mM. UV-Vis Absorption spectra were recorded in a 1-cm path length quartz cuvette, on a Varian Cary 50 UV-Vis spectrophotometer or Varian Cary 60 UV-Vis spectrophotometer equipped with a Peltier heat exchange unit. Photoswitching experiments were carried out using mounted LEDs from Thorlabs that were operated with a LEDD1B power supply (maximal current: 1200 mA): M365L2, M365L3 and M415L4. Collimation of the irradiation beam to a diameter of 50 mm was achieved using a SM2F32-A collimation adapter from Thorlabs.

Excitation spectra were measured on an Agilent Technology Cary eclipse. Fluorescence lifetimes and emission spectra were measured using a FluoroTime 300 (PicoQuant, Berlin, Germany) system with a hybrid PMT detector. The samples were excited using a pulsed solid-state laser at 407 nm (LDH-P-C-405) or 374 nm (LDH-P-C-375).

Compound 1-*meta*. *m*-iodo-DHA **3** (38.2 mg, 100 µmol), SP-alkyne **4** (34.6 mg, 100 µmol), $Pd_2(dba)_3$ (9.2 mg, 10 µmol), triphenylarsine (24.5 mg, 80.0 µmol) and Et_3N (0.07 mL) were dissolved in dry THF (5.00 mL) in a flame dried microwave vial. The reaction mixture was degassed with nitrogen for 30 min. The vial was closed and heated in microwave synthesizer at 70°C for 12 hours. The reaction mixture was filtered through a plug of Celite (eluent EtOAc), concentrated on Celite and purified by flash column chromatography (30% EtOAc in heptane), to give **1**-*meta* (17.0 mg, 28%) as yellow oil. R_f = 0.34 (30% EtOAc in heptane). ^{1}H-NMR (500 MHz, CD_3CN): δ 8.10 (d, *J* = 2.8 Hz, 1H), 8.02 (dd, *J* = 9.0, 2.8 Hz, 1H), 7.88 (t, *J* = 1.8 Hz, 1H), 7.77 (dt, *J* = 7.7, 1.6 Hz, 1H), 7.58 (dt, *J* = 7.7, 1.6 Hz, 1H), 7.53 (t, *J* = 7.7 Hz, 1H), 7.43 (dd, *J* = 8.0, 1.7 Hz, 1H), 7.32 (d, *J* = 1.7 Hz, 1H), 7.18 (s, 1H), 7.08 (d, *J* = 10.4 Hz, 1H), 6.77 (d, *J* = 9.0 Hz, 1H), 6.65 – 6.59 (m, 2H), 6.51 (dd, *J* = 11.2, 6.1 Hz, 1H), 6.46 (d, *J* = 6.3 Hz, 1H), 6.35 (ddd, *J* = 10.3, 6.1, 2.3 Hz, 1H), 5.95 (d, *J* = 10.4 Hz, 1H), 5.80 (dd, *J* = 10.3, 3.7 Hz, 1H), 3.89 (dt, *J* = 3.9, 2.0 Hz, 1H), 2.78 (s, 3H), 1.30 (s, 3H), 1.18 (s, 3H) ppm. ^{13}C-NMR (126 MHz, CD_3CN) δ 160.29, 149.43, 142.23, 139.51, 139.46, 137.94, 135.38, 133.21, 133.18, 132.18, 131.86,131.76, 130.58, 129.42, 129.39, 128.56, 126.64, 126.46, 126.00, 125.76, 123.73, 122.91, 122.00, 120.72, 119.97, 116.26, 116.11, 113.99, 113.75, 108.02, 107.28, 92.55, 87.14, 52.91, 51.85, 46.28, 29.07, 25.99, 19.94 ppm. HRMS (ESI): *m/z* calculated for $C_{39}H_{28}N_4O_3$, $[M+H]^+$ = 601.22342; found: m/z= 601.22305.

Compound 1-*ortho*. *Ortho*-iodo-DHA **5** (80.4 mg, 210 µmol) and SP-alkyne **4** (73.4 mg, 212 µmol) were transferred to a microwave vial. Freshly distilled and nitrogen-degassed THF and nitrogen-degassed triethylamine (150 µL, 1.08 mmol) were added to the vial, and the reaction mixture was degassed with nitrogen for 20 min. Triphenylarsine (49.6 mg, 162 µmol) and $Pd_2(dba)_3$ (29.5 mg, 32.2 µmol) were added to the vial and the reaction mixture was degassed with nitrogen for additional 20 min and heated to 80 °C for 16 h in a microwave synthesizer.

The reaction mixture was filtered through a plug of Celite (eluent: THF), and the solvent was evaporated under reduced pressure. The residue was dissolved in CH_2Cl_2 and evaporated on Celite. Repeated purification by flash column chromatography (20% EtOAc/heptane) gave product **1**-*ortho* as a mixture of diastereoisomers, labelled A and B (26.3 mg, 43.8 µmol, 21%) as an orange solid. R_f = 0.25 (20% EtOAc/heptane). M. p.: 132 °C (decomp.).^{1}H-NMR (500 MHz, CD_3CN) δ 8.09 (d, *J* = 2.8 Hz, 1H, isomer A+B), 8.01 (dd, *J* = 9.0, 2.8 Hz, 1H, isomer A+B), 7.83–7.80 (m, 1H, isomer A+B), 7.68–7.65 (m, 1H, isomer A+B), 7.53–7.45 (m, 2H, isomer A+B), 7.37 and 7.36 (s, 1H, isomer A+B), 7.31 (dd, *J* = 8.0, 1.6 Hz, 1H, isomer A+B), 7.15 and 7.14 (d, *J* = 1.6 Hz, 1H, isomer A+B), 7.07 (d, *J* = 10.4 Hz, 1H, isomer A+B), 6.755 and 6.749 (d, *J* = 9.0 Hz, 1H, isomer A+B), 6.63–6.56 (m, 2H, isomer A+B), 6.53–6.47 (m, 2H, isomer A+B), 6.37–6.31 (m, 1H, isomer A+B), 5.92 (d, *J* = 10.4 Hz, 1H, isomer A+B), 5.78 and 5.76 (t, *J* = 3.1 Hz, 1H, isomer A+B), 3.90 and 3.89 (dt, *J* = 3.7, 1.8 Hz, 1H, isomer A+B), 2.75 (s, 3H, isomer A+B), 1.213 and 1.207 (s, 3H, isomer A+B), 1.13 (s, 3H, isomer A+B) ppm (additional splitting of the signals are due to the presence of diastereoisomers). ^{13}C-NMR (126 MHz, CD_3CN) δ 160.3, 149.5, 142.3, 139.9, 139.8, 139.5, 139.4, 138.7, 138.6, 138.0, 134.0, 133.9, 133.8, 133.7, 132.9, 131.85, 131.82, 130.58, 130.57 129.5, 129.2, 128.70, 128.66, 128.20, 128.17, 126.7, 126.1, 124.7, 124.6, 123.8, 122.7, 122.6, 121.9, 121.08, 121.06, 120.0, 116.31, 116.29, 116.22, 116.21, 114.08, 114.06, 113.8, 108.1, 107.3, 96.74, 96.71, 87.28, 87.25, 52.86, 52.85 51.62, 51.60, 49.02, 49.01, 29.1, 26.1, 20.0 ppm (signals missing due to overlap). HRMS (ESI): *m/z* calculated for $C_{39}H_{28}N_4O_3$ $[M+H]^+$ = 601.22342; found: m/z= 601.22399.

Compound 9. Iodomethane (0.88 mL, 14 mmol) was added to a solution of compound **8** (2.02 g, 7.05 mmol) in acetonitrile (16 mL). The reaction mixture was heated by microwave irradiation at 100 °C for 3 h. After cooling to rt, the solvent and excess iodomethane were removed under reduced pressure. The residue was suspended in cold EtOAc (25 mL), filtered under vacuum and washed with cold EtOAc (10 mL) to give product **9** (2.54 g, 5.94 mmol, 84%) as an orange solid. M.p.: 250 °C (decomp., color change started at 225 °C). ^{1}H-NMR (500 MHz, $CDCl_3$) δ 8.00 (d, *J* = 8.1 Hz, 1H), 7.92 (s, 1H), 7.62 (d, *J* = 8.1 Hz, 1H), 4.29 (s, 3H), 3.15 (s, 3H), 1.67 (s, 6H), 1.37 (s, 12H) ppm. ^{13}C-NMR (126 MHz, $CDCl_3$) δ 197.2, 144.1, 140.6, 136.2, 129.0, 114.4, 84.7, 54.7, 37.4, 25.0, 23.2, 17.5 ppm (one signal missing). HRMS (MALDI$^+$): *m/z* calculated for $C_{18}H_{27}BNO_2$ $[M]^+$ = 300.21294; found 300.21268.

Compound 6. Compound **9** (4.01 g, 9.39 mmol), 2-hydroxy-5-nitrobenzaldehyde (1.64 g, 9.81 mmol), and piperidine (2.6 mL, 26 mmol) were added to nitrogen-degassed absolute EtOH (250 mL). The reaction mixture was heated to reflux for 5 h under a nitrogen atmosphere. After cooling to rt, the solvent was evaporated under reduced pressure. The residue was dissolved in CH_2Cl_2 (200 mL) and washed with a saturated aqueous solution of Na_2CO_3 (150 mL), H_2O (200 mL), and brine (2 x 150 mL). The water phases separated from the washes with brine were combined and additionally extracted with CH_2Cl_2 (100 mL, 50 mL). The combined organic phases were dried over $MgSO_4$ and concentrated *in vacuo* together with Celite. Purification by flash column chromatography (20% EtOAc/heptane) gave product **6** (1.59 g, 3.54 mmol, 38%) as a yellow solid. R_f = 0.40 (20% EtOAc/heptane). M.p.: 192 °C (decomp., color change started at 187 °C). ^{1}H-NMR (500 MHz, CD_3CN) δ 8.07 (d, *J* = 2.7 Hz, 1H), 7.98 (dd, *J* = 9.0, 2.7 Hz, 1H), 7.58 (dd, *J* = 7.8, 1.1 Hz, 1H), 7.42 (d, *J* = 1.1 Hz, 1H), 7.06 (d, *J* = 10.4 Hz, 1H), 6.72 (d, *J* = 9.0 Hz, 1H), 6.60 (d, *J* = 7.8 Hz, 1H), 5.93 (d, *J* = 10.4 Hz, 1H), 2.74 (s, 3H), 1.30 (s, 12 H), 1.27 (s, 3H), 1.14 (s, 3H) ppm. ^{13}C-NMR (126 MHz, CD_3CN) δ 160.5, 151.6, 142.2, 136.7, 136.5, 129.3, 128.3, 126.6, 123.7, 122.3, 120.0, 116.3, 107.6, 107.4, 84.3, 52.9, 29.0, 26.2, 25.24, 25.20, 20.1 ppm (signals missing due to overlap). HRMS (MALDI$^+$): *m/z* calculated for $C_{25}H_{29}BN_2O_5$, $[M+H]^+$ = 449.22423; found 449.22369.

Compound 2-*para*. A solution of compound **7** (308 mg, 806 μmol), compound **6** (301 mg, 671 μmol), and Aliquat 336 (58.6 mg, 145 μmol) in toluene (30 mL) and water (3 mL) was degassed with nitrogen for 30 min. K_3PO_4 (285 mg, 1.34 mmol), $Pd(OAc)_2$ (14.4 mg, 64.1 μmol), and RuPhos (58.0 mg, 124 μmol) were added to the reaction vessel, and the mixture was degassed with nitrogen for additional 30 min and then heated to 50 °C for 23 h. After cooling to rt, the reaction mixture was filtered through a plug of Celite (eluent: CH_2Cl_2), and the solvent was removed under reduced pressure. The residue was dissolved in CH_2Cl_2 and concentrated *in vacuo* together with Celite. Repeated purification by flash column chromatography (toluene) gave product **2**-*para* as a mixture of undistinguishable diastereoisomers (59.9 mg, 104 μmol, 15%) as a yellow solid. Further purification of mixed fractions by flash column chromatography (first column: 30% EtOAc/heptane, second column: 20-100% EtOAc/heptane) gave product *para*-azulene-**10** (35.8 mg, 65.1 μmol, 10%) as a dark purple solid. **2**-*para*: R_f = 0.35 (toluene). M.p.: 200 °C (decomp., color change started at 190 °C). ^{1}H-NMR (500 MHz, $CDCl_3$) δ 8.05–8.01 (m, 2H), 7.81–7.78 (m, 2H), 7.70–7.67 (m, 2H), 7.50 (dd, *J* = 8.1, 1.8 Hz, 1H), 7.36 (d, *J* = 1.8 Hz, 1H), 6.96 (d, *J* = 10.3 Hz, 1H), 6.91 (s, 1H), 6.82 (d, *J* = 8.7 Hz, 1H), 6.65 (d, *J* = 8.1 Hz, 1H), 6.58 (dd, *J* = 11.2, 6.4 Hz, 1H), 6.48 (d, *J* = 11.2, 6.1 Hz, 1H), 6.35 (d, *J* = 6.4 Hz, 1H), 6.32 (ddd, *J* = 10.2, 6.1, 2.0 Hz, 1H), 5.89 (d, *J* = 10.3 Hz, 1H), 5.84 (dd, *J* = 10.2, 3.8 Hz, 1H), 3.81 (dt, *J* = 3.8, 2.0 Hz, 1H), 2.80 (s, 3H), 1.36 (s, 3H), 1.25 (s, 3H) ppm. ^{13}C-NMR (126 MHz, $CDCl_3$) δ 159.8, 148.0, 143.3, 141.2, 140.2, 139.1, 137.2, 131.8, 131.6, 131.1, 130.9, 128.6, 128.4, 127.8, 127.3, 127.1, 126.8, 126.1, 122.9, 121.4, 120.8, 120.6, 119.6, 118.7, 115.7, 115.4, 113.0, 107.5, 106.5, 52.5, 51.3, 45.3, 29.1, 26.2, 20.1 ppm (signals missing due to overlap). HRMS (MALDI$^+$): *m/z* calculated for $C_{37}H_{28}N_4O_3$, $[M+H]^+$ = 577.22342; found 577.22347. *para*-azulene-**10**: R_f = 0.31 (30% EtOAc/heptane). M.p.: 258-260 °C. ^{1}H-NMR (500 MHz, $CDCl_3$) δ 8.64 (d, *J* = 9.7 Hz, 1H), 8.41 (d, *J* = 9.7 Hz, 1H), 8.18–8.14 (m, 2H), 8.06–8.01 (m, 2H), 7.80–7.74 (m, 3H), 7.60 (s, 1H), 7.550 (dd, *J* = 8.0, 1.8 Hz, 1H), 7.547 (t, *J* = 9.7 Hz, 1H), 7.49 (t, *J* = 9.7 Hz, 1H), 7.41 (d, *J* = 1.8 Hz, 1H), 6.96 (d, *J* = 10.3 Hz, 1H), 6.82 (d, *J* = 8.7 Hz, 1H), 6.67 (d, *J* = 8.0 Hz, 1H), 5.90 (d, *J* = 10.3 Hz, 1H), 2.81 (s, 3H), 1.38 (s, 3H), 1.26 (s, 3H) ppm. ^{13}C-NMR (126 MHz, $CDCl_3$) δ 159.8, 152.0, 147.9, 146.1, 142.8, 142.7, 141.2, 138.7, 137.8, 137.2, 135.6, 132.4, 132.3, 129.2, 128.6, 128.2, 128.0, 127.3, 127.1, 126.1, 122.9, 121.5, 120.7, 118.8, 118.4, 116.4, 115.7, 107.5, 106.6, 94.1, 52.5, 29.1, 26.2, 20.1 ppm (signals missing due to overlap). HRMS (MALDI$^+$): *m/z* calculated for $C_{36}H_{27}N_3O_3$, $[M+H]^+$ = 550.21252; found 550.21214.

Compound 2-*meta*. A solution of **3** (54.6 mg, 143 μmol), compound **6** (50.8 mg, 113 μmol), and Aliquat 336 (12.3 mg, 30.4 μmol) in toluene (5.0 mL) and water (0.5 mL) was degassed with nitrogen for 30 min. K_3PO_4 (50.0 mg, 236 μmol), $Pd(OAc)_2$ (3.2 mg, 14 μmol), and RuPhos (9.41 mg, 20.2 μmol) were added to the reaction vessel, and the mixture was degassed with nitrogen for additional 30 min and then stirred at rt for 45 h. The reaction mixture was filtered through a plug of Celite (eluent: CH_2Cl_2), and the solvent was removed under reduced pressure. The residue was dissolved in CH_2Cl_2 and concentrated *in vacuo* together with Celite. Purification by flash column chromatography (toluene) gave product **2**-*meta* as a mixture of undistinguishable diastereoisomers (13.3 mg, 23.1 μmol, 20%) as a yellow solid. Rf = 0.37 (toluene). M.p.: 132 °C (decomp., color change started at 120 °C). ^{1}H-NMR (500 MHz, $CDCl_3$) δ 8.05–8.01 (m, 2H), 7.90 (s, 1H), 7.67 (d, *J* = 7.8 Hz, 1H), 7.63 (d, *J* = 7.8 Hz, 1H), 7.52 (t, *J* = 7.8 Hz, 1H), 7.47 (dd, *J* = 8.0, 1.6 Hz, 1H), 7.32 (d, *J* = 1.6 Hz, 1H), 6.954 (d, *J* = 10.2 Hz, 1H), 6.949 (s, 1H), 6.83 (d, *J* = 8.7 Hz, 1H), 6.65 (d, *J* = 8.0 Hz, 1H), 6.58 (dd, *J* = 11.2, 6.3 Hz, 1H), 6.49 (dd, *J* = 11.2, 6.1 Hz, 1H), 6.37 (d, *J* = 6.3 Hz, 1H), 6.32 (ddd, *J* = 10.1, 6.1, 1.9 Hz, 1H), 5.89 (d, *J* = 10.2 Hz, 1H), 5.84 (dd, *J* = 10.1, 3.7 Hz, 1H), 3.82 (dt, *J* = 3.7, 1.9 Hz, 1H), 2.80 (s, 3H), 1.36 (s, 3H), 1.25 (s, 3H) ppm. ^{13}C-NMR (126 MHz, $CDCl_3$) δ 159.8, 147.9, 142.9, 141.2, 140.6, 138.9, 137.2, 132.6, 132.3, 131.07, 131.05, 131.0, 129.7, 128.62, 128.57,

127.8, 127.2, 126.1, 124.8, 124.3, 122.9, 121.5, 121.1, 120.8, 119.7, 118.8, 115.7, 115.4, 113.0, 107.5, 106.6, 52.5, 51.3, 45.5, 29.1, 26.1, 20.1 ppm (missing signals due to overlap). HRMS (MALDI$^+$): *m/z* calculated for $C_{37}H_{28}N_4O_3$, $[M+H]^+$ = 577.22342; found 577.22253. Replacement of the solvent mixture with toluene/EtOH/water (3/1.5/1) and stirring the reaction at rt for 20 h gave product **2**-*meta* in 9%. In addition, further repeated purification of mixed fractions by flash column chromatography (30% EtOAc/heptane) gave *para*-azulene-**11** (42.4 mg, 77.1 µmol, 23%) as a dark purple solid. Rf = 0.47 (40% EtOAc/heptane). M.p.: 146-149 °C. ^{1}H-NMR (500 MHz, $CDCl_3$) δ 8.68 (d, *J* = 9.6 Hz, 1H), 8.44 (d, *J* = 9.7 Hz, 1H), 8.29 (d, *J* = 1.4 Hz, 1H), 8.03 (d, *J* = 8.6 Hz, 1H), 8.02 (s, 1H), 7.97 (d, *J* = 7.6 Hz, 1H), 7.80 (t, *J* = 9.8 Hz, 1H), 7.67 (d, *J* = 7.6 Hz, 1H), 7.62 (s, 1H), 7.61–7.54 (m, 3H), 7.50 (t, *J* = 9.7 Hz, 1H), 7.44 (s, 1H), 6.95 (d, *J* = 10.3 Hz, 1H), 6.82 (d, *J* = 8.6 Hz, 1H), 6.67 (d, *J* = 8.0 Hz, 1H), 5.90 (d, *J* = 10.3 Hz, 1H), 2.80 (s, 3H), 1.38 (s, 3H), 1.27 (s, 3H) ppm. ^{13}C-NMR (126 MHz, $CDCl_3$) δ 159.9, 152.6, 147.7, 145.8, 142.70, 142.68, 141.1, 139.0, 138.1, 137.1, 136.0, 134.9, 132.8, 129.7, 128.5, 128.2, 127.92, 127.87, 127.22, 127.19, 126.7, 126.1, 122.9, 121.6, 120.9, 118.8, 118.3, 116.7, 115.7, 107.5, 106.6, 94.6, 52.5, 29.1, 26.2, 20.1 ppm. HRMS (MALDI$^+$): *m/z* calculated for $C_{36}H_{27}N_3O_3$, $[M+H]^+$ = 550.21252; found 550.21256.

NMR spectra

A full assignment of the ^{1}H-NMR spectra is reported based on the 1D and 2D spectra. Protons on the DHA unit are indicated as d1, d2, d3 etc, and protons on the SP unit as s1, s2, s3 etc.

- Compound **1**-*meta*

2-(3-((1',3',3'-trimethyl-6-nitrospiro[chromene-2,2'-indoline]-5'-yl)ethynyl)phenyl)azulene-1,1(8aH)-dicarbonitrile

^{1}H NMR (500 MHz, CD_3CN) δ 8.10 (d, *J* = 2.8 Hz, 1H, H s5), 8.02 (dd, *J* = 9.0, *J* = 2.8 Hz, 1H, H s7), 7.88 (t, *J* = 1.8 Hz, 1H, H d2'), 7.77 (dt, *J* = 7.7, *J* = 1.6 Hz, 1H, H d6'), 7.58 (dt, *J* = 7.7, *J* =1.6 Hz, 1H, H d4'), 7.53 (t, *J* = 7.7 Hz, 1H, H d5'), 7.43 (dd, *J* = 8.0, *J* = 1.7 Hz, 1H, H s6'), 7.32 (d, *J* = 1.7 Hz, 1H, H s4'), 7.18 (s, 1H, H d3), 7.08 (d, *J* = 10.4 Hz, 1H, H s4), 6.77 (d, *J* = 9.0 Hz, 1H, H s8), 6.65–6.59 (m, 2H, H d5 and s7'), 6.51 (dd, *J* = 11.2, 6.1 Hz, 1H, H d6), 6.46 (d, *J* = 6.3 Hz, 1H, H d4), 6.35 (ddd, *J* = 10.3, 6.1, *J* = 2.3 Hz, 1H, H d7), 5.95 (d, *J* = 10.4 Hz, 1H, H s3), 5.80 (dd *J* = 10.3, 3.7 Hz, 1H, H d8), 3.89 (dt, *J* = 3.9, *J* = 2.0 Hz, 1H, H d8a), 2.78 (s, 3H, N s1'-CH_3), 1.30 (s, 3H, C s3' -CH_3), 1.18 (s, 3H, C s3'-CH_3) ppm.

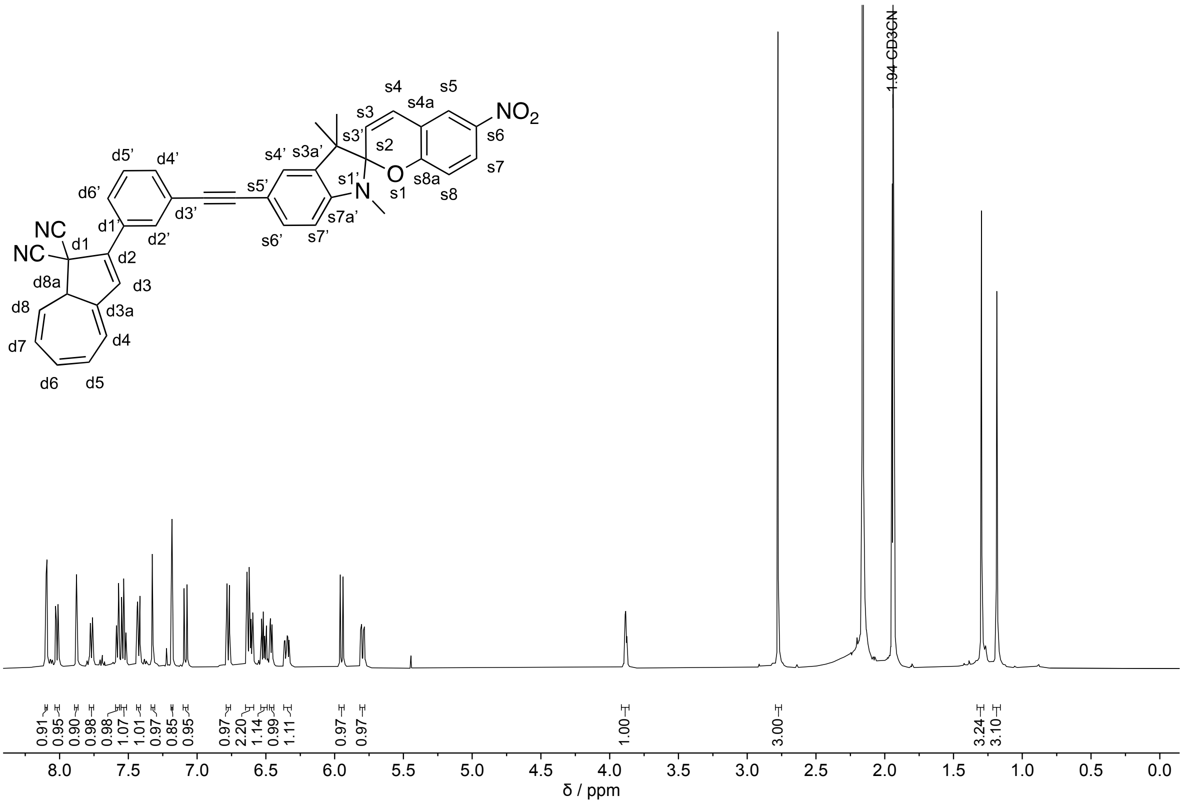

Fig. S1. ^{1}H NMR spectrum (500 MHz, CD_3CN) of compound **1**-*meta*.

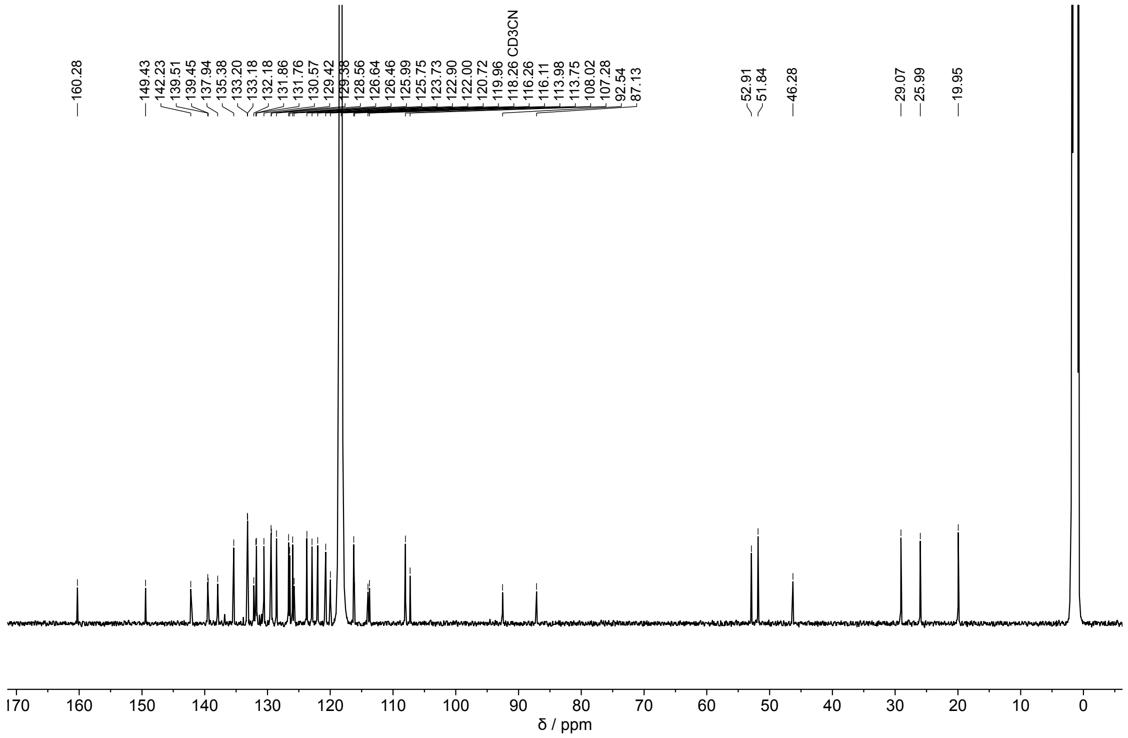

Fig. S2. ^{13}C NMR spectrum (126 MHz, CD_3CN) of compound **1**-*meta*.

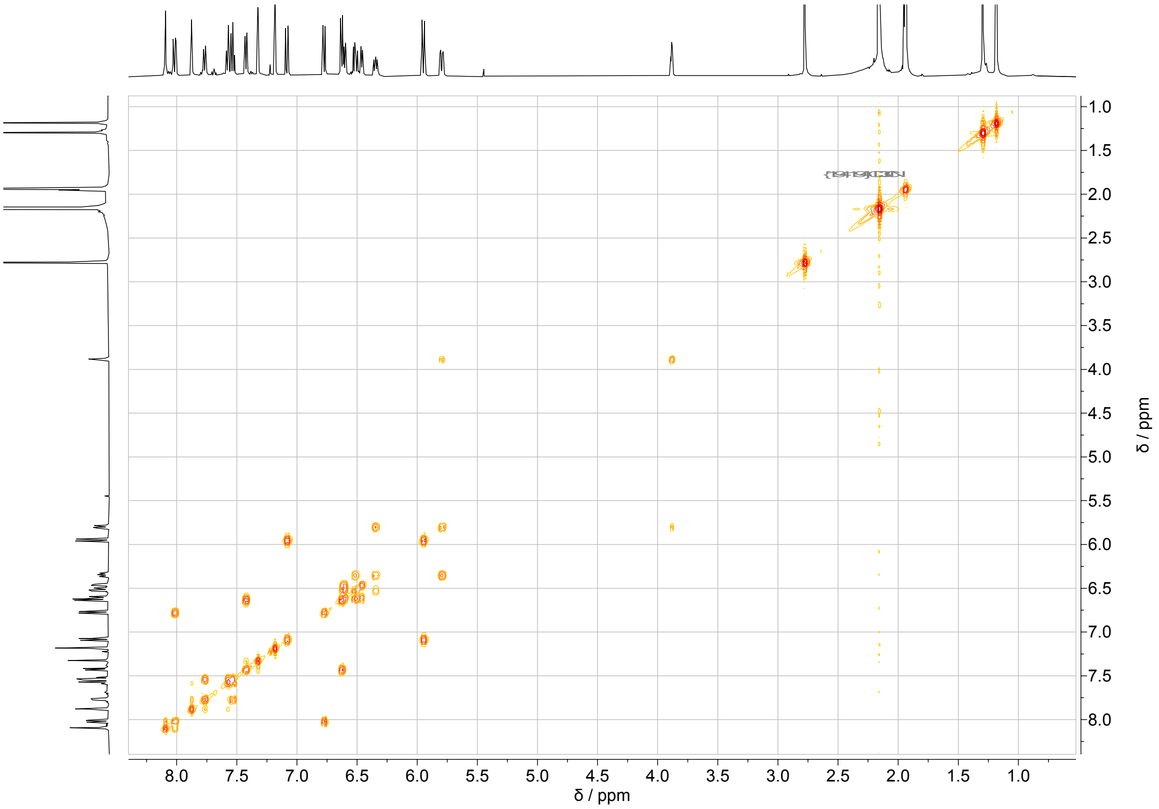

Fig. S3. COSY spectrum (500 MHz, CD_3CN) of compound **1**-*meta*.

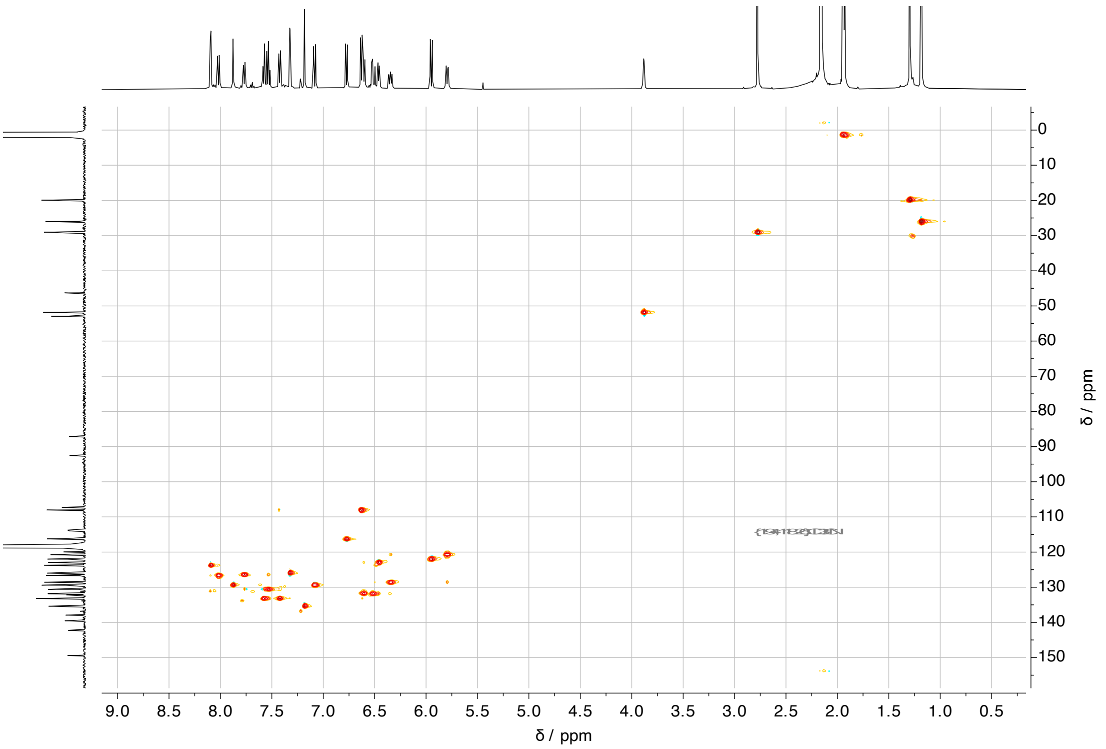

Fig. S4. HSQC spectrum (500/126 MHz, CD_3CN) of compound **1**-*meta*.

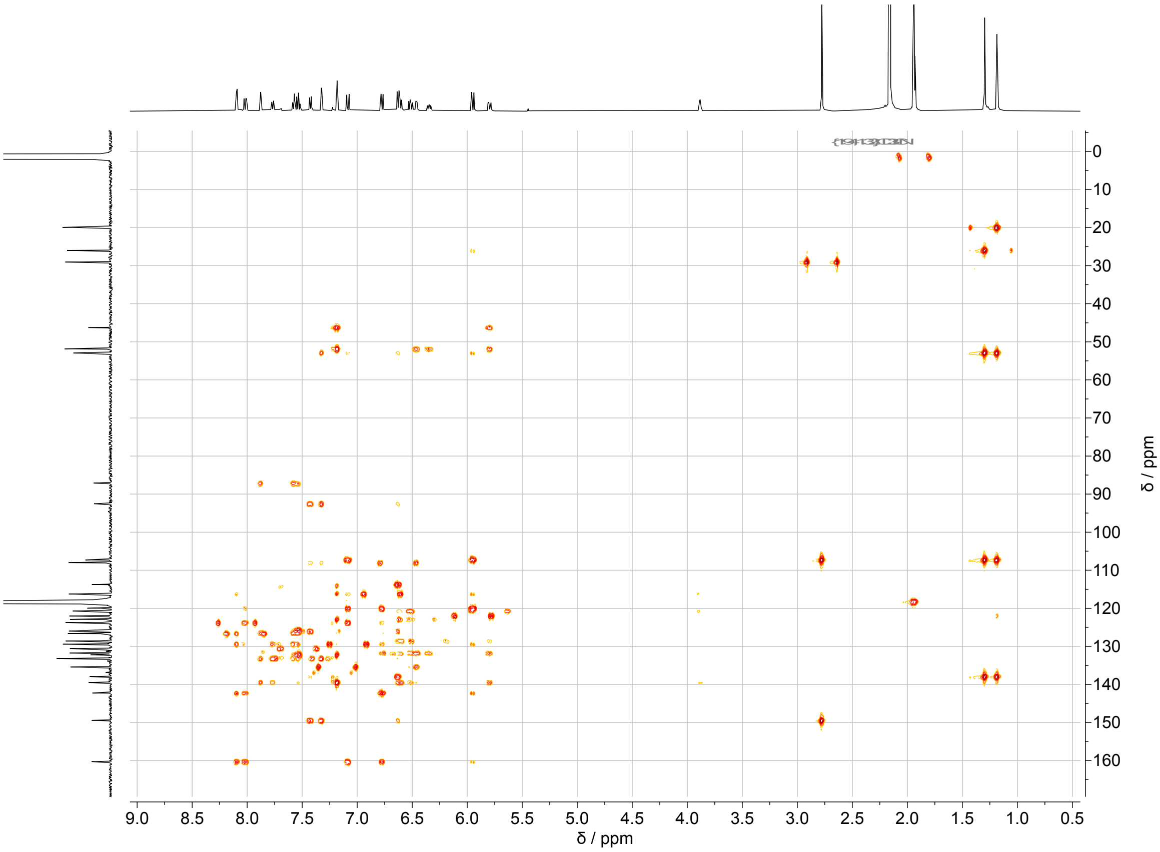

Fig. S5. HMBC spectrum (500/126 MHz, CD_3CN) of compound **1**-*meta*.

- Compound **1**-*ortho*

2-(2-((1',3',3'-trimethyl-6-nitrospiro[chromene-2,2'-indoline]-5'-yl)ethynyl)phenyl)azulene-1,1(8a*H*)-dicarbonitrile

^{1}H NMR (500 MHz, CD_3CN) δ 8.09 (d, *J* = 2.8 Hz, 1H, H s5, isomer A+B), 8.01 (dd, *J* = 9.0, 2.8 Hz, 1H, H s7, isomer A+B), 7.83–7.80 (m, 1H, H Ph, isomer A+B), 7.68–7.65 (m, 1H, H Ph, isomer A+B), 7.53–7.45 (m, 2H, H Ph, isomer A+B), 7.37 and 7.36 (s, 1H, H d3, isomer A+B), 7.31 (dd, *J* = 8.0, 1.6, 1H, H s6', isomer A+B), 7.15 and 7.14 (d, *J* = 1.6 Hz, 1H, H s4', isomer A+B), 7.07 (d, *J* =10.4 Hz, 1H, H s4, isomer A+B), 6.755 and 6.749 (d, *J* = 9.0 Hz, 1H, H s8, isomer A+B), 6.63–6.56 (m, 2H, H s7' and d4 or d5, isomer A+B), 6.53–6.47 (m, 2H, H d6 and d4 or d5, isomer A+B), 6.37–6.31 (m, 1H, H d7, isomer A+B), 5.92 (d, *J* = 10.4, 1H, H s3, isomer A+B), 5.78 and 5.76 (t, *J* = 3.1 Hz, 1H, H d8, isomer A+B), 3.90 and 3.89 (dt, *J* = 3.7, 1.8 Hz, 1H, H 8a, isomer A+B), 2.75 (s, 3H, N s1'-CH_3, isomer A+B), 1.213 and 1.207 (s, 3H, C s3' -CH_3, isomer A+B), 1.13 (s, 3H, C s3' -CH_3, isomer A+B) ppm (additional splitting of the signals are due to the presence of diastereoisomers).

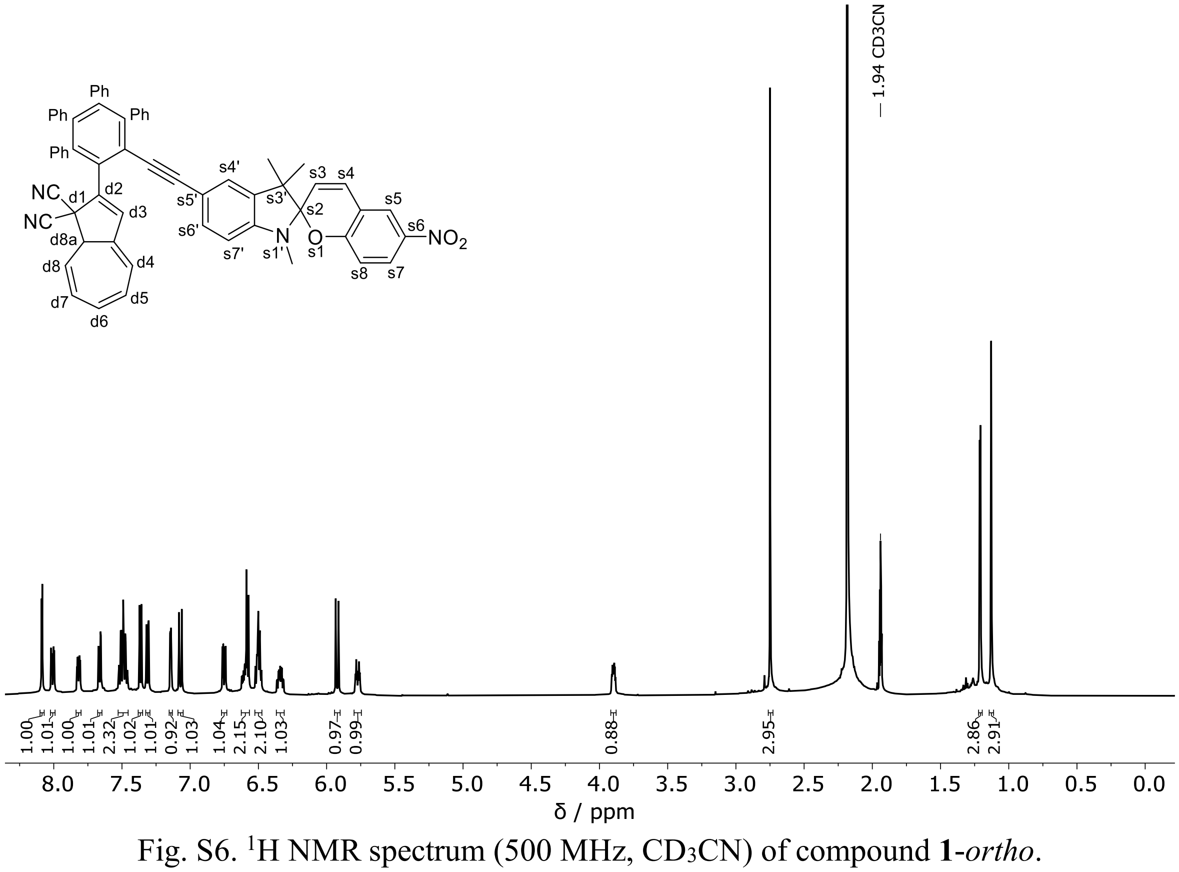

Fig. S6. ^{1}H NMR spectrum (500 MHz, CD_3CN) of compound **1**-*ortho*.

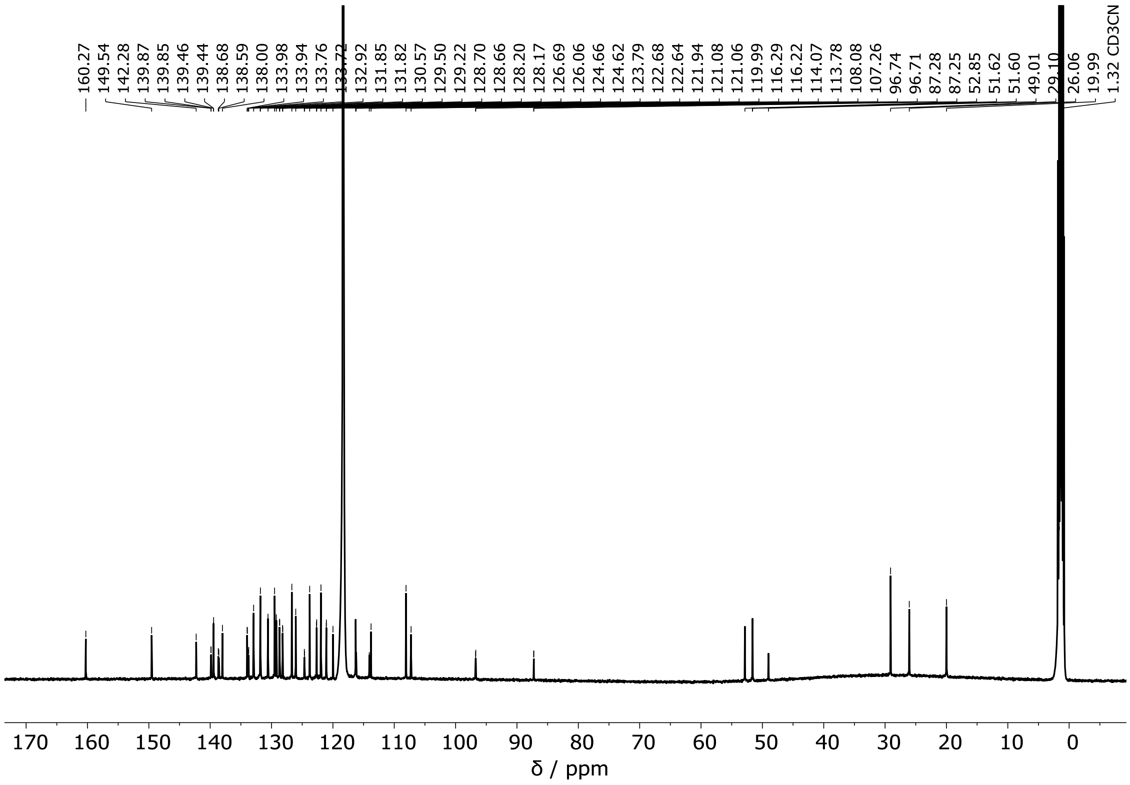

Fig. S7. ^{13}C NMR spectrum (126 MHz, CD_3CN) of compound **1**-*ortho*.

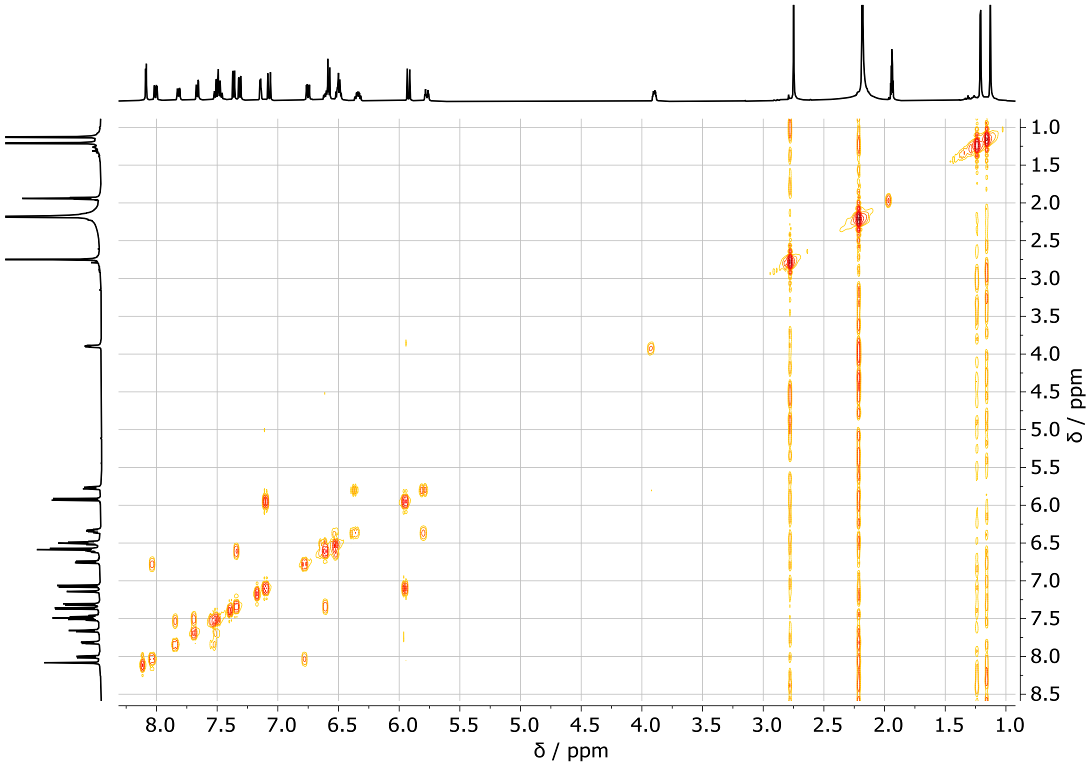

Fig. S8. COSY spectrum (500 MHz, CD_3CN) of compound **1**-*ortho*.

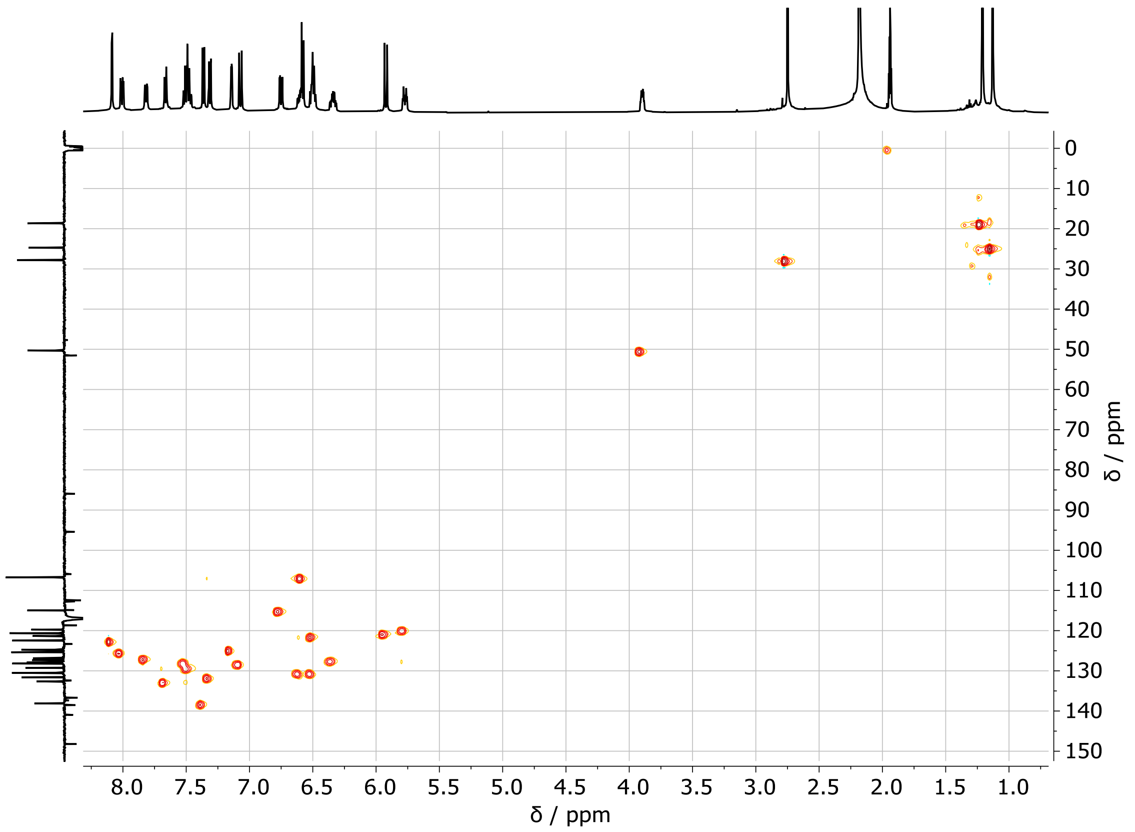

Fig. S9. HSQC spectrum (500/126 MHz, CD_3CN) of compound **1**-*ortho*.

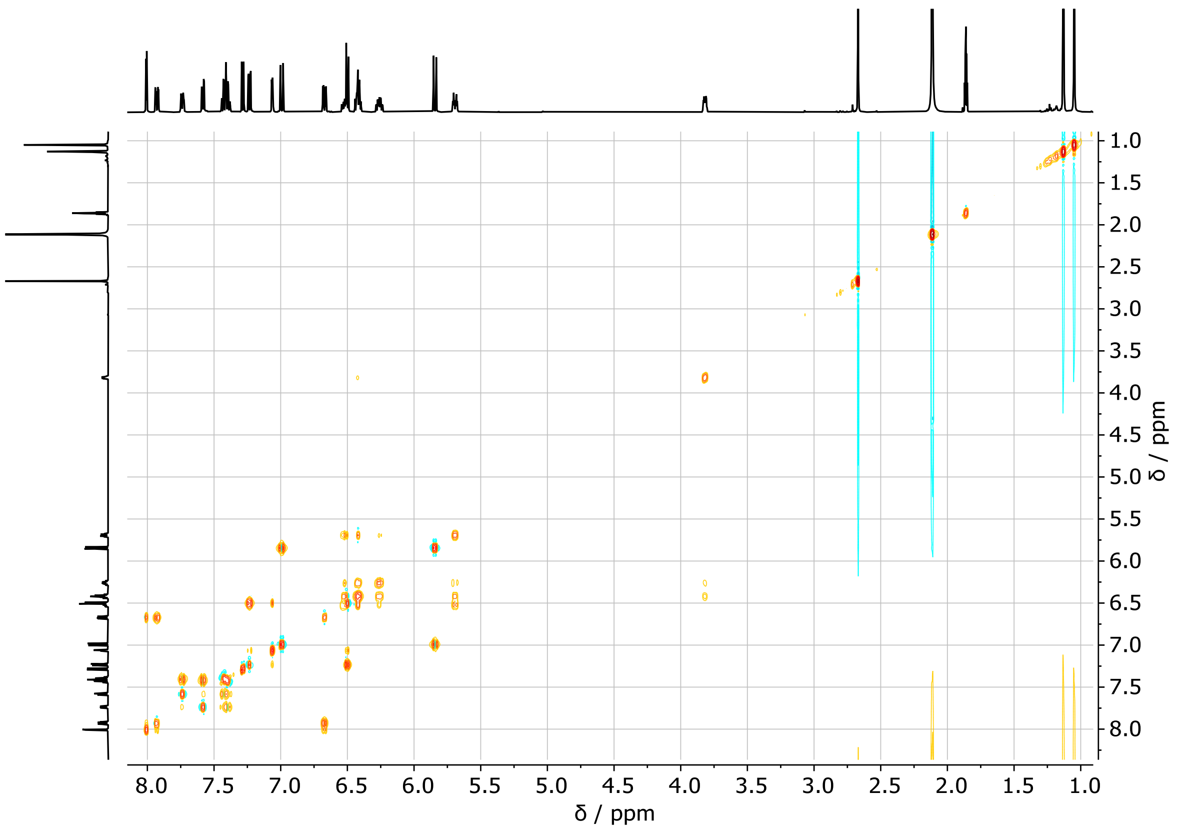

Fig. S10. TOCSY spectrum (500 MHz, CD_3CN) of compound **1**-*ortho*.

- 1,2,3,3-tetramethyl-5-(4,4,5,5-tetramethyl-1,3,2-dioxaborolan-2-yl)-3*H*-indol-1-ium iodide (**9**)

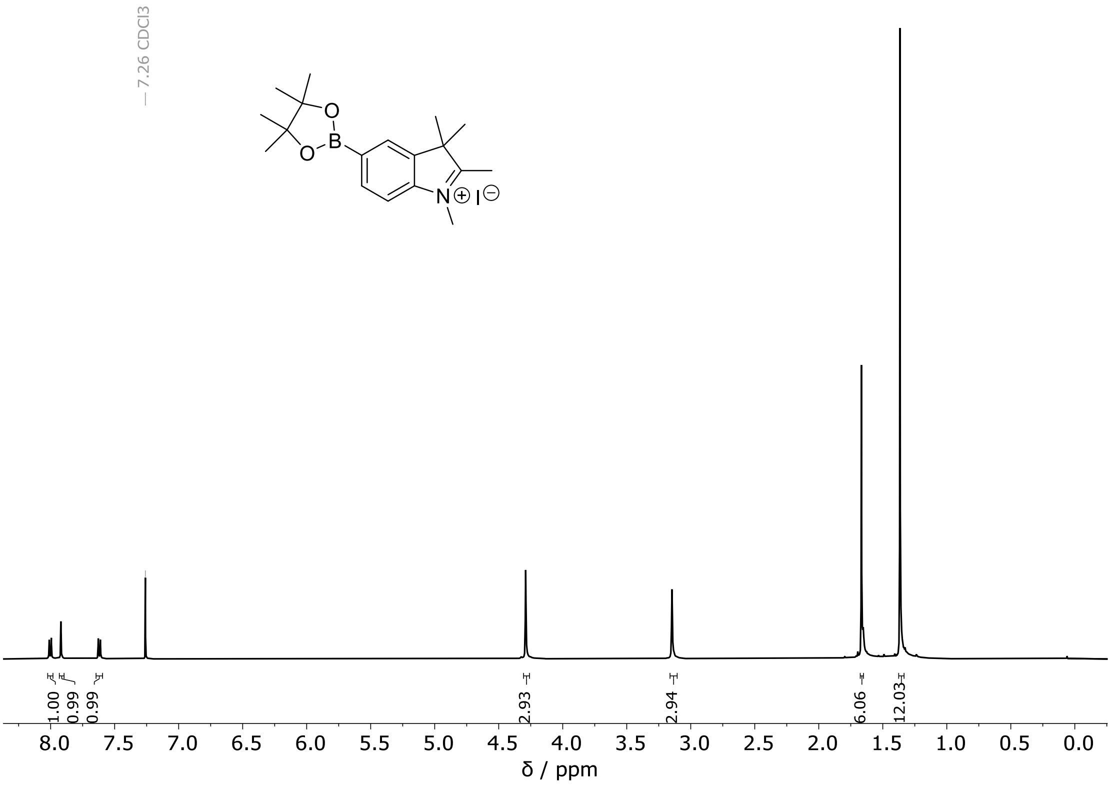

Fig. S11. ^{1}H NMR spectrum (500 MHz, $CDCl_3$) of compound **9**.

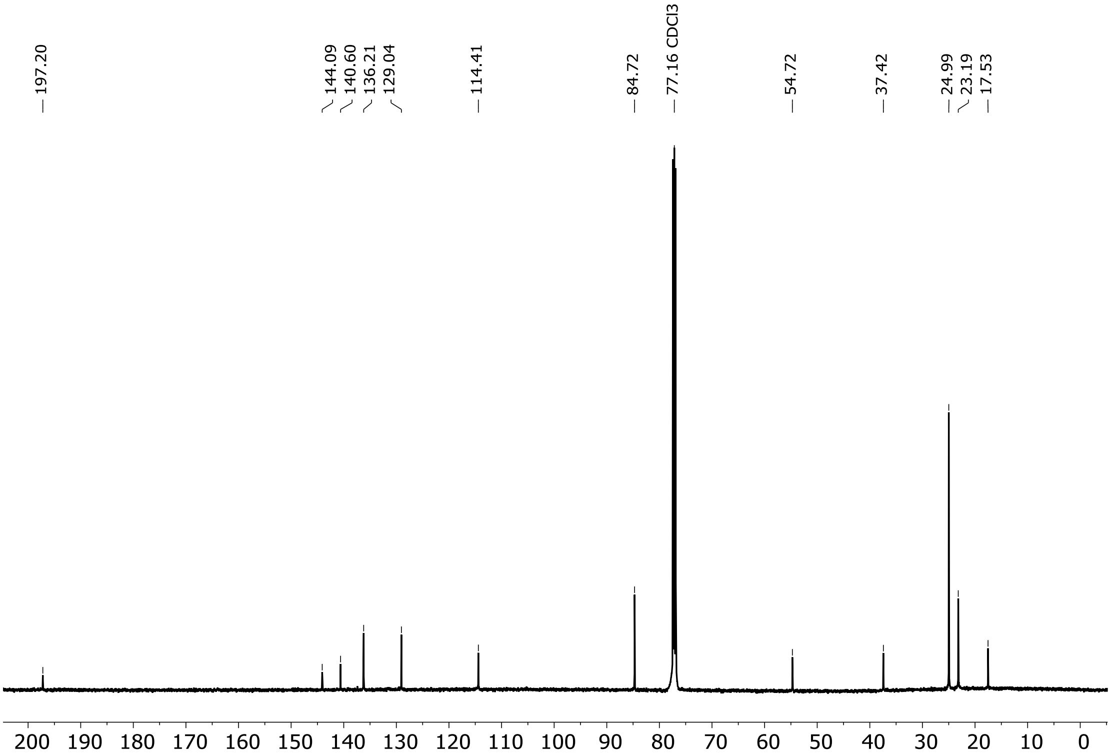

Fig. S12. ^{13}C NMR spectrum (126 MHz, $CDCl_3$) of compound **9**.

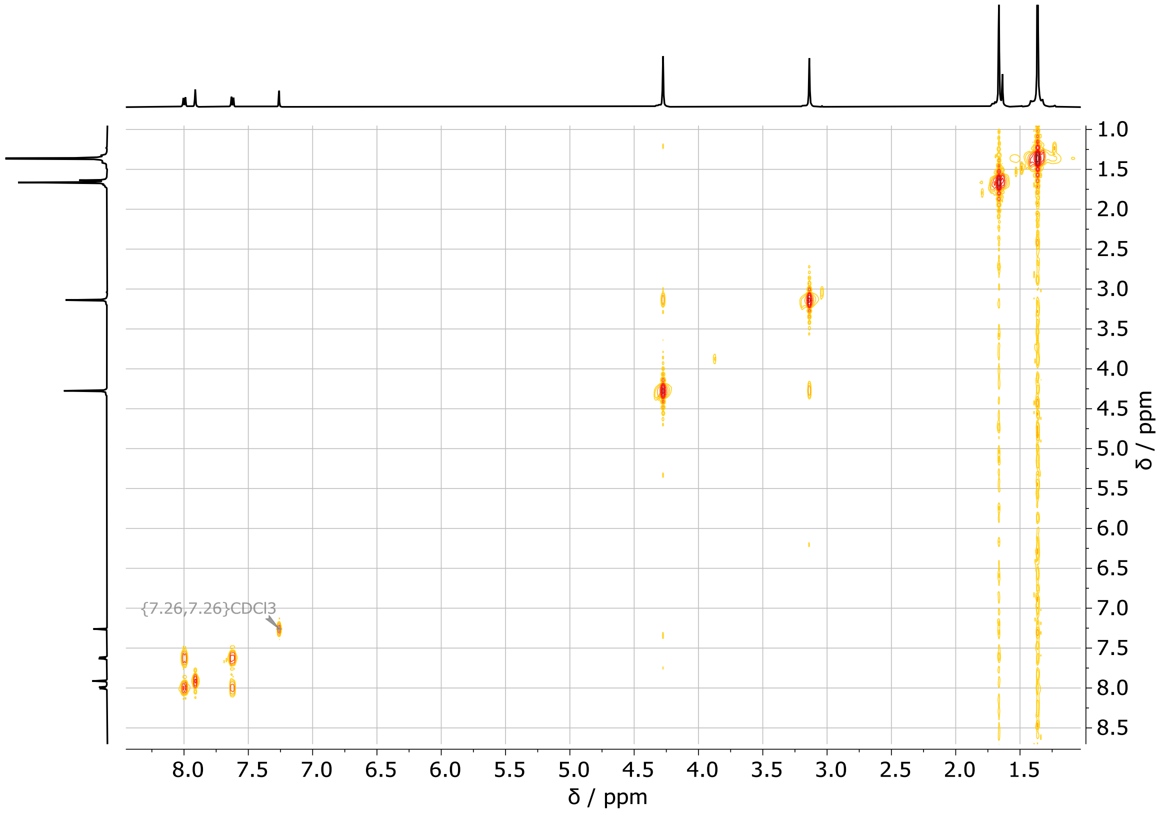

Fig. S13. COSY spectrum (500 MHz, $CDCl_3$) of compound **9**.

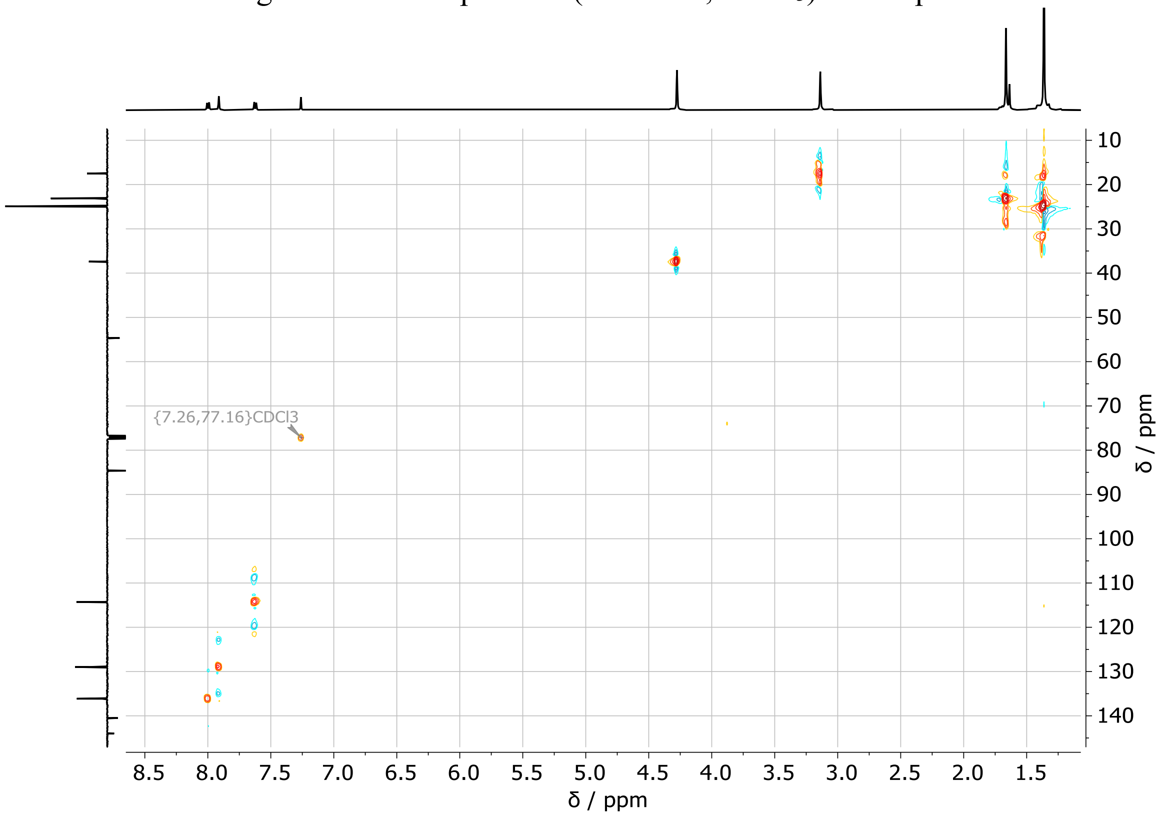

Fig. S14. HSQC spectrum (500/126 MHz, $CDCl_3$) of compound **9**.

- 1',3',3'-trimethyl-6-nitro-5'-(4,4,5,5-tetramethyl-1,3,2-dioxaborolan-2-yl)spiro[chromene-2,2'-indoline] (**6**)

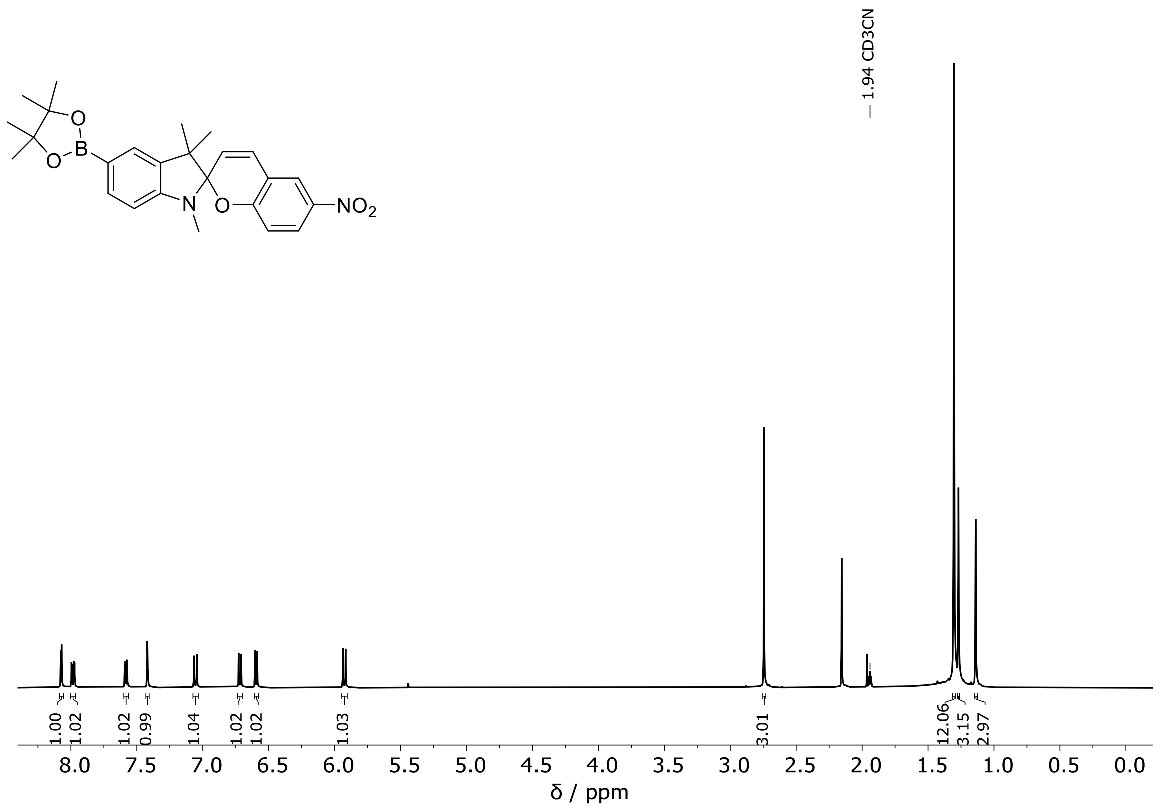

Fig. S15. ^{1}H NMR spectrum (500 MHz, CD_3CN) of compound **6**.

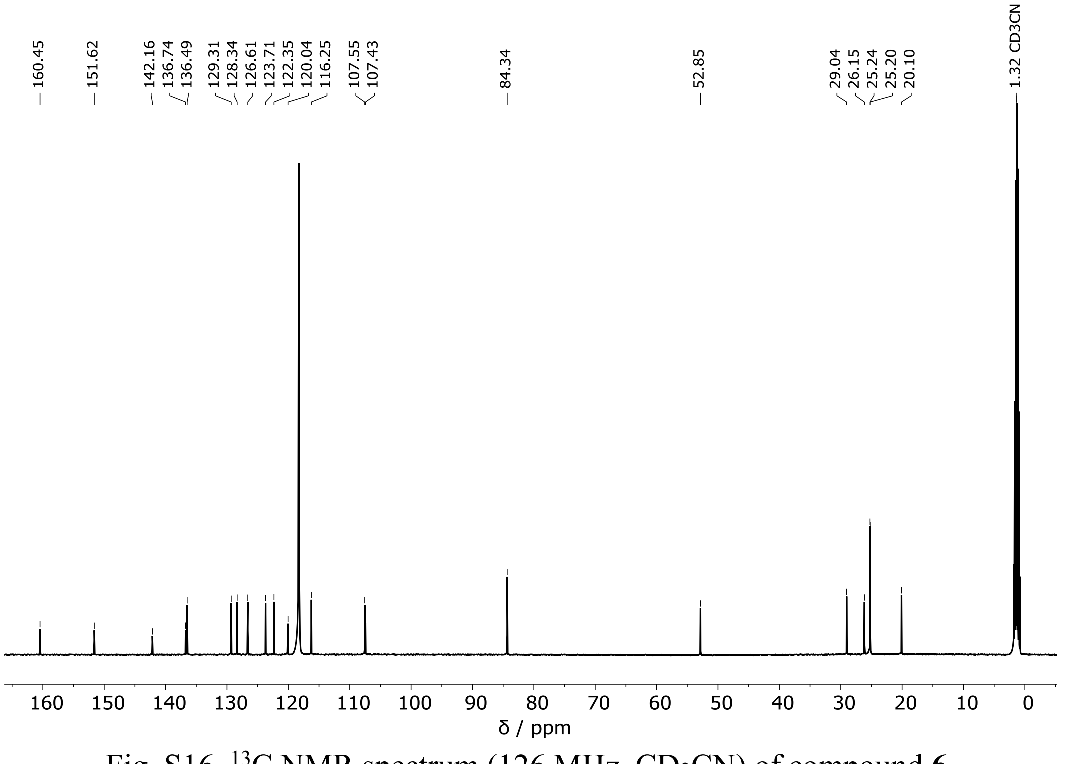

Fig. S16. ^{13}C NMR spectrum (126 MHz, CD_3CN) of compound **6**.

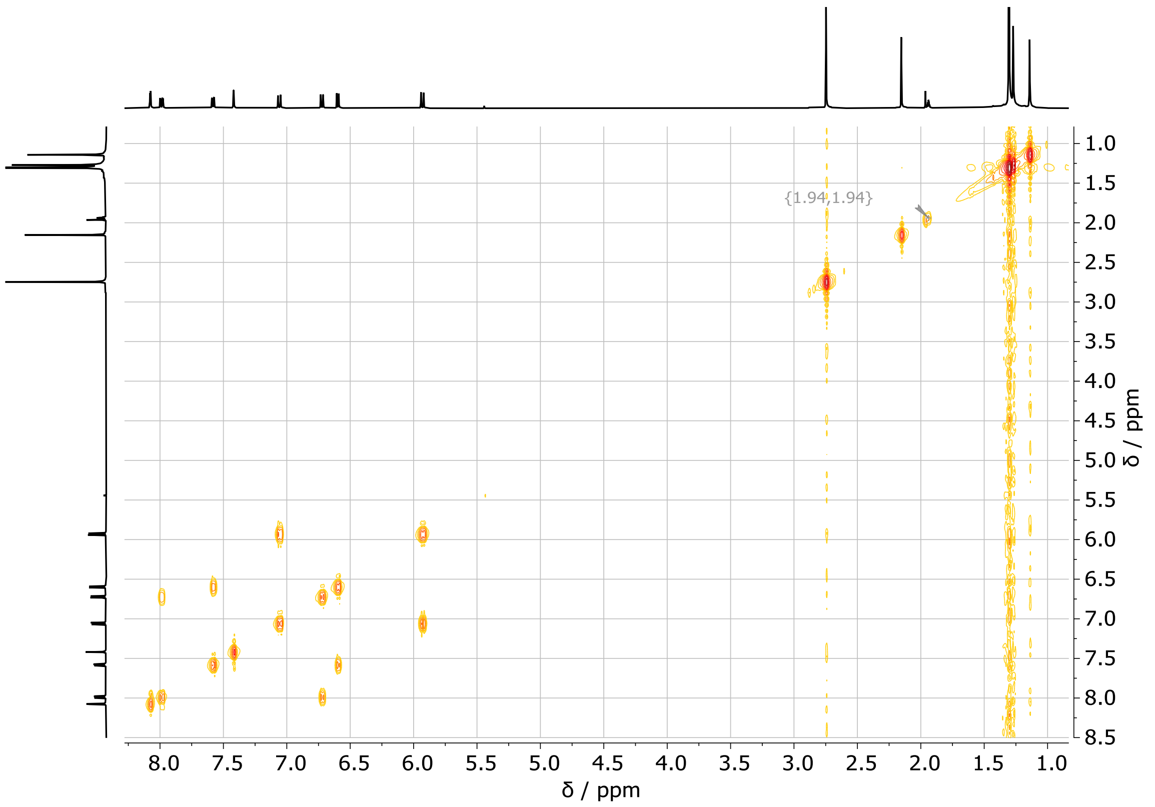

Fig. S17. COSY spectrum (500 MHz, CD_3CN) of compound **6**.

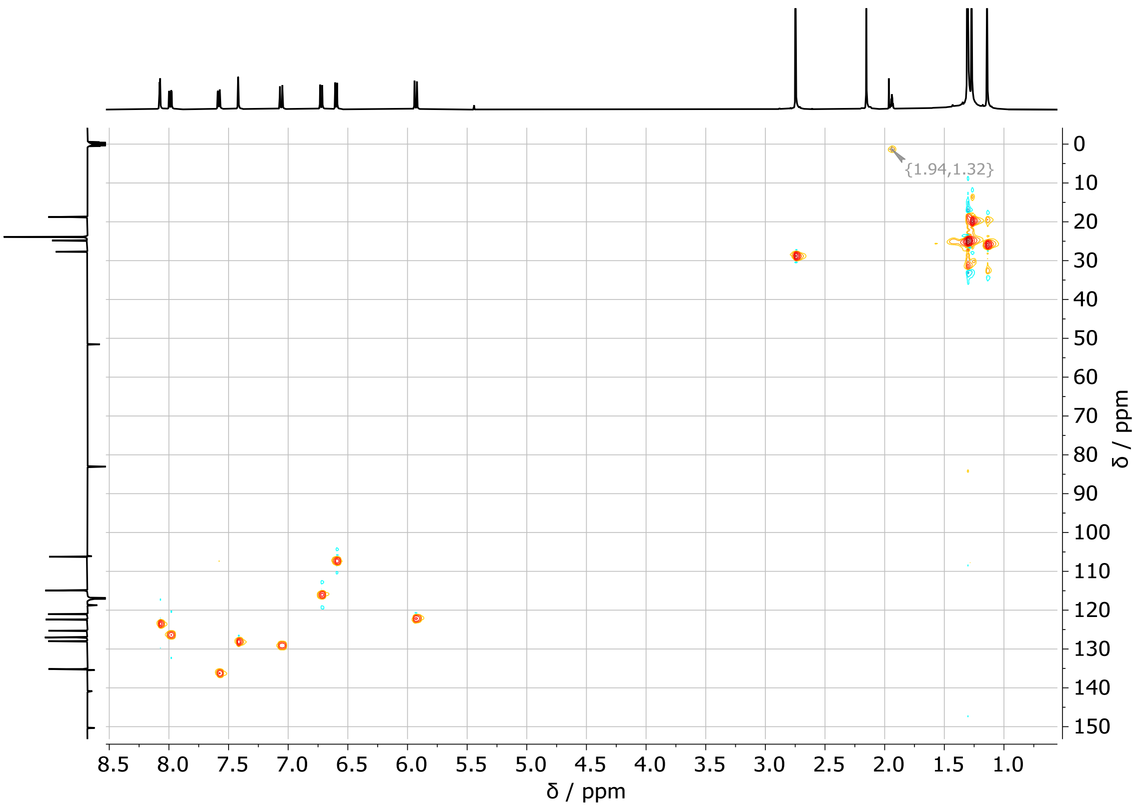

Fig. S18. HSQC spectrum (500/126 MHz, CD_3CN) of compound **6**.

- Compound **2**-*meta*

2-(3-(1',3',3'-trimethyl-6-nitrospiro[chromene-2,2'-indoline]-5'-yl)phenyl)azulene-1,1(8a*H*)-dicarbonitrile

^{1}H NMR (500 MHz, $CDCl_3$) δ 8.05–8.01 (m, 2H), 7.90 (s, 1H), 7.67 (d, *J* = 7.8 Hz, 1H), 7.63 (d, *J* = 7.8 Hz, 1H), 7.52 (t, *J* = 7.8 Hz, 1H), 7.47 (dd, *J* = 8.0, 1.6 Hz, 1H), 7.32 (d, *J* = 1.6 Hz, 1H), 6.954 (d, *J* = 10.2 Hz, 1H), 6.949 (s, 1H), 6.83 (d, *J* = 8.7 Hz, 1H), 6.65 (d, *J* = 8.0 Hz, 1H), 6.58 (dd, *J* = 11.2, 6.3 Hz, 1H), 6.49 (dd, *J* = 11.2, 6.1 Hz, 1H), 6.37 (d, *J* = 6.3 Hz, 1H), 6.32 (ddd, *J* = 10.1, 6.1, 1.9 Hz, 1H), 5.89 (d, *J* = 10.2 Hz, 1H), 5.84 (dd, *J* = 10.1, 3.7 Hz, 1H), 3.82 (dt, *J* = 3.7, 1.9 Hz, 1H), 2.80 (s, 3H), 1.36 (s, 3H), 1.25 (s, 3H) ppm.

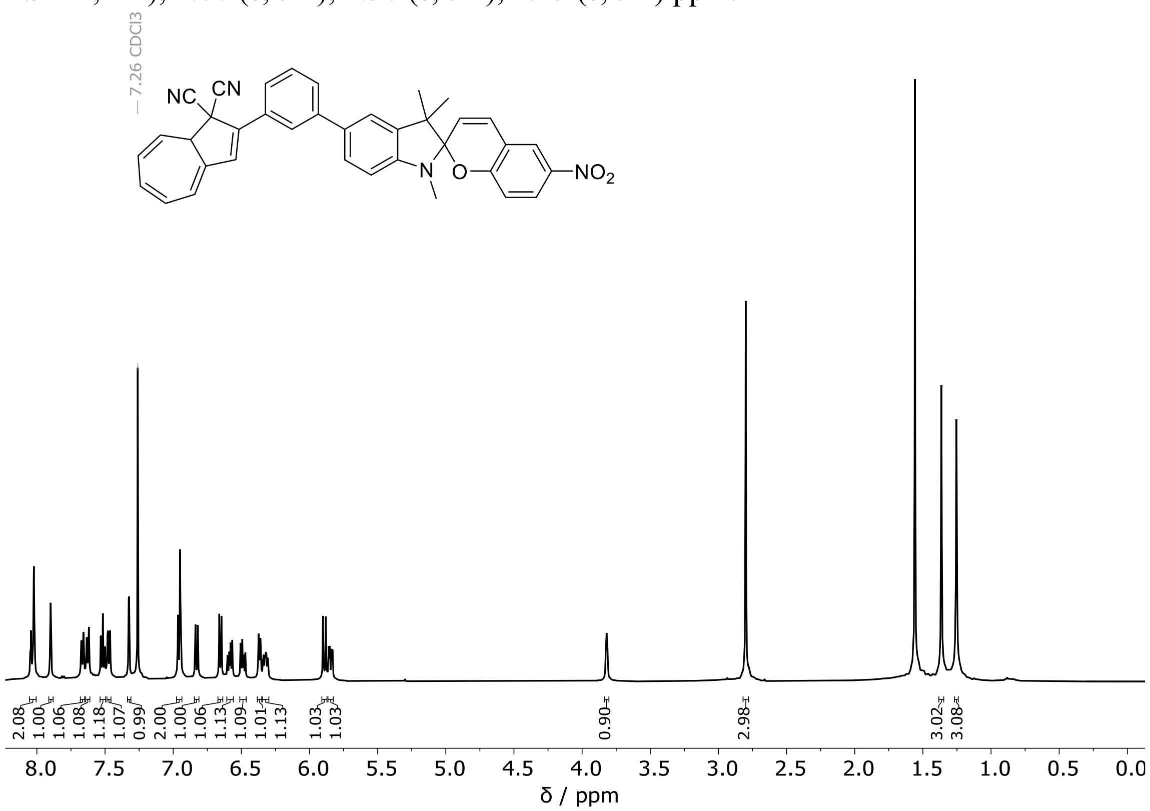

Fig. S19. ^{1}H NMR spectrum (500 MHz, $CDCl_3$) of compound **2**-*meta*.

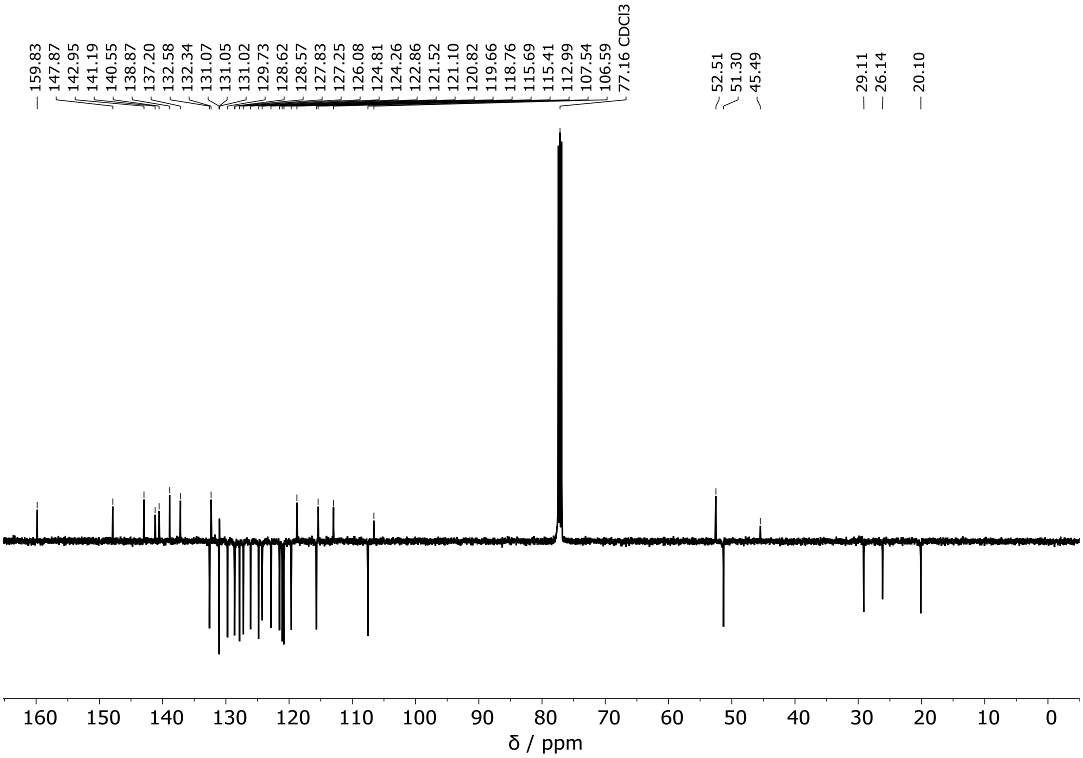

Fig. S20. ^{13}C APT spectrum (126 MHz, $CDCl_3$) of compound **2**-*meta*.

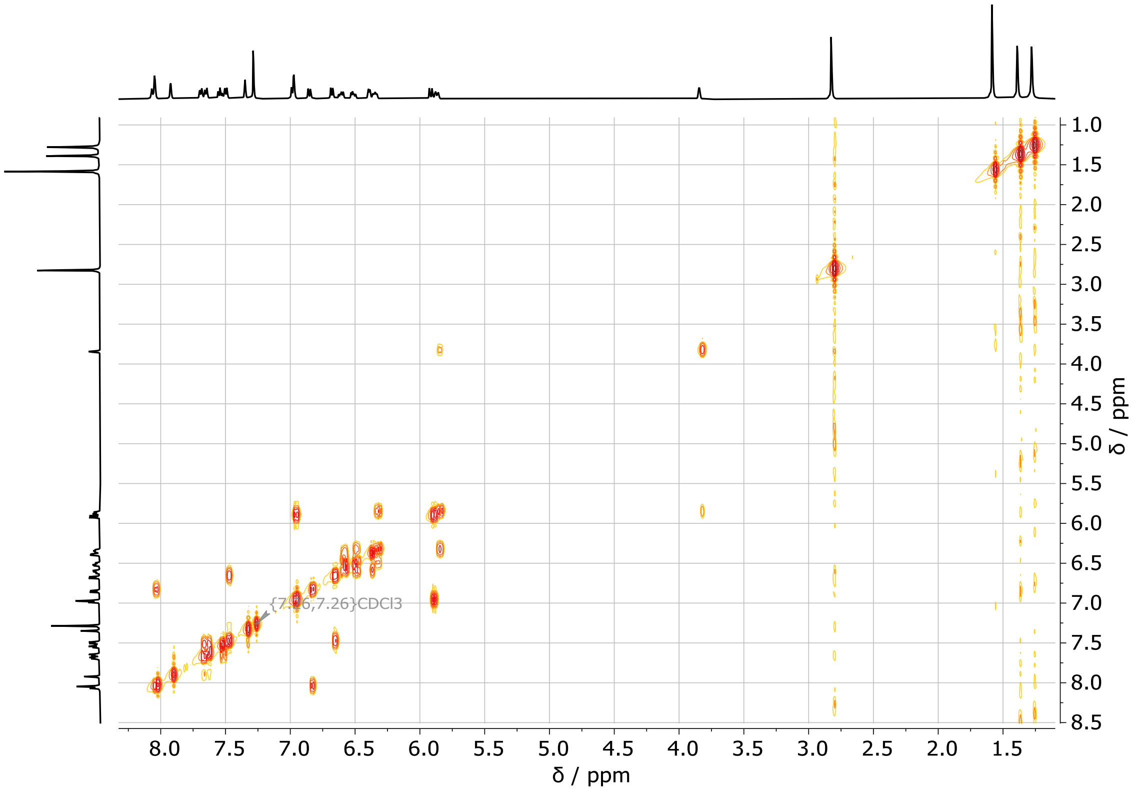

Fig. S21. COSY spectrum (500 MHz, $CDCl_3$) of compound **2**-*meta*.

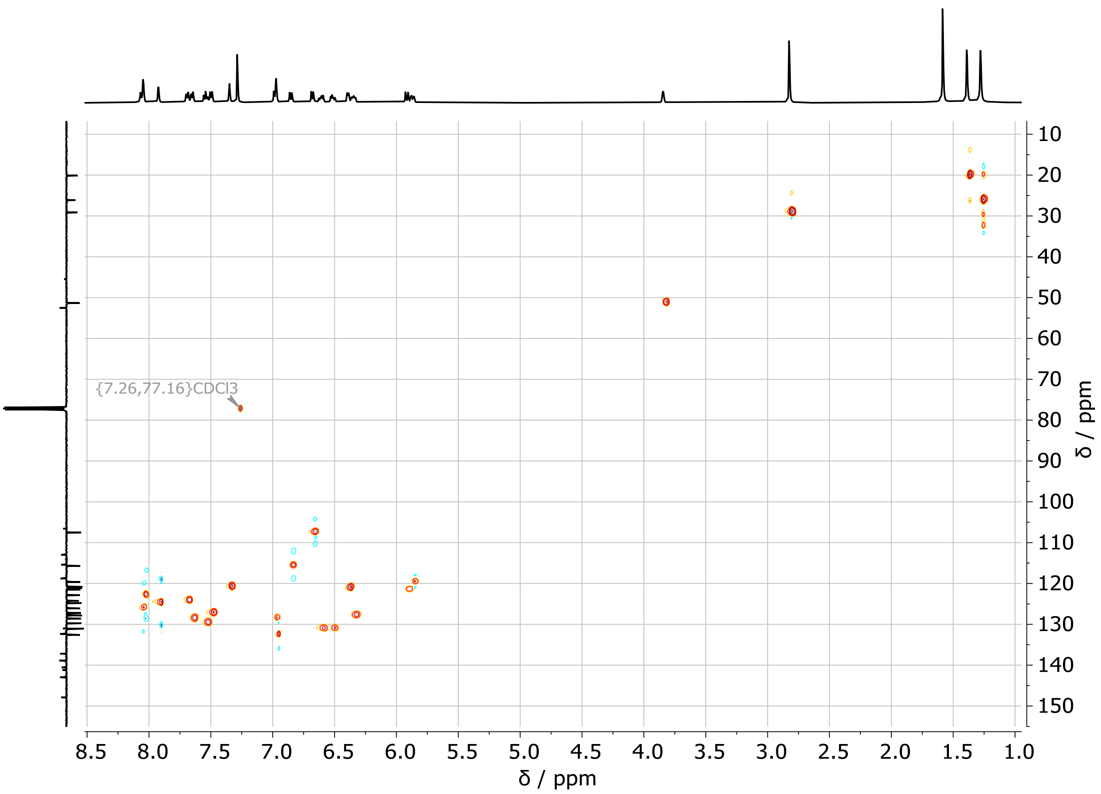

Fig. S22. HSQC spectrum (500/126 MHz, $CDCl_3$) of compound **2**-*meta*.

- Compound **11**.

2-(3-(1',3',3'-trimethyl-6-nitrospiro[chromene-2,2'-indoline]-5'-yl)phenyl)azulene-1-carbonitrile

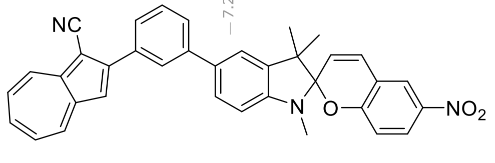

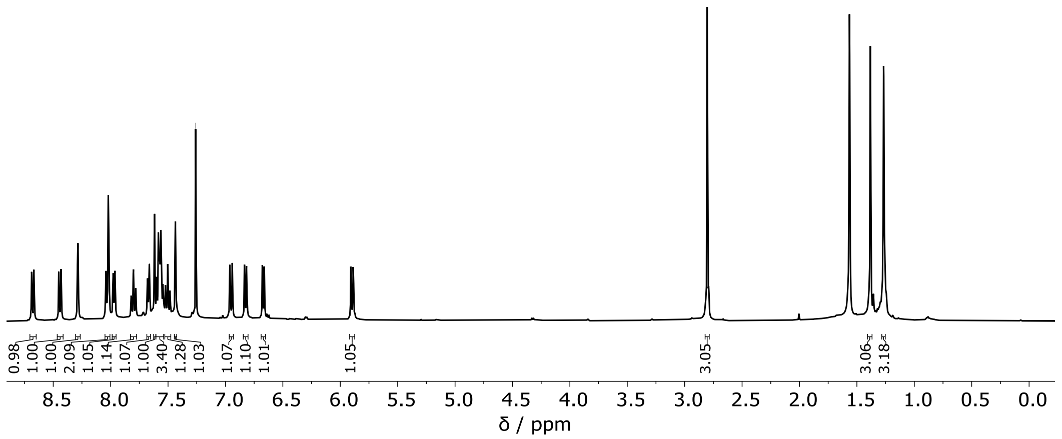

Fig. S23. ^{1}H NMR spectrum (500 MHz, $CDCl_3$) of compound **11**.

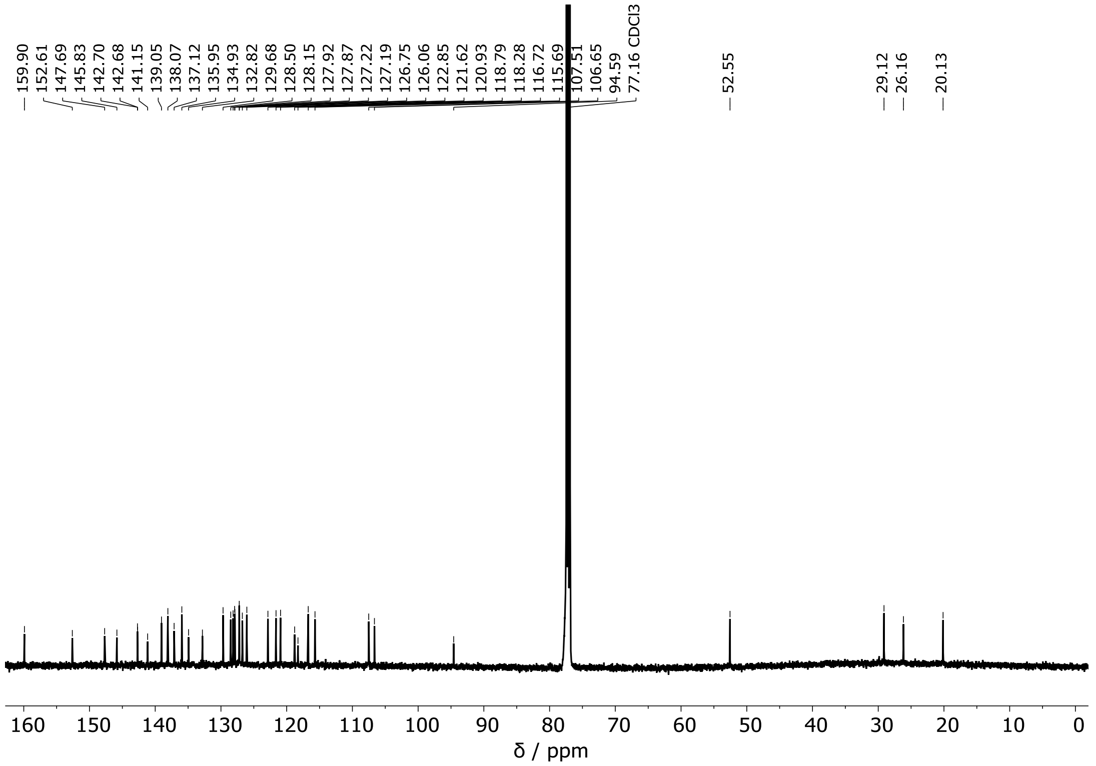

Fig. S24. ^{13}C NMR spectrum (126 MHz, $CDCl_3$) of compound **11**.

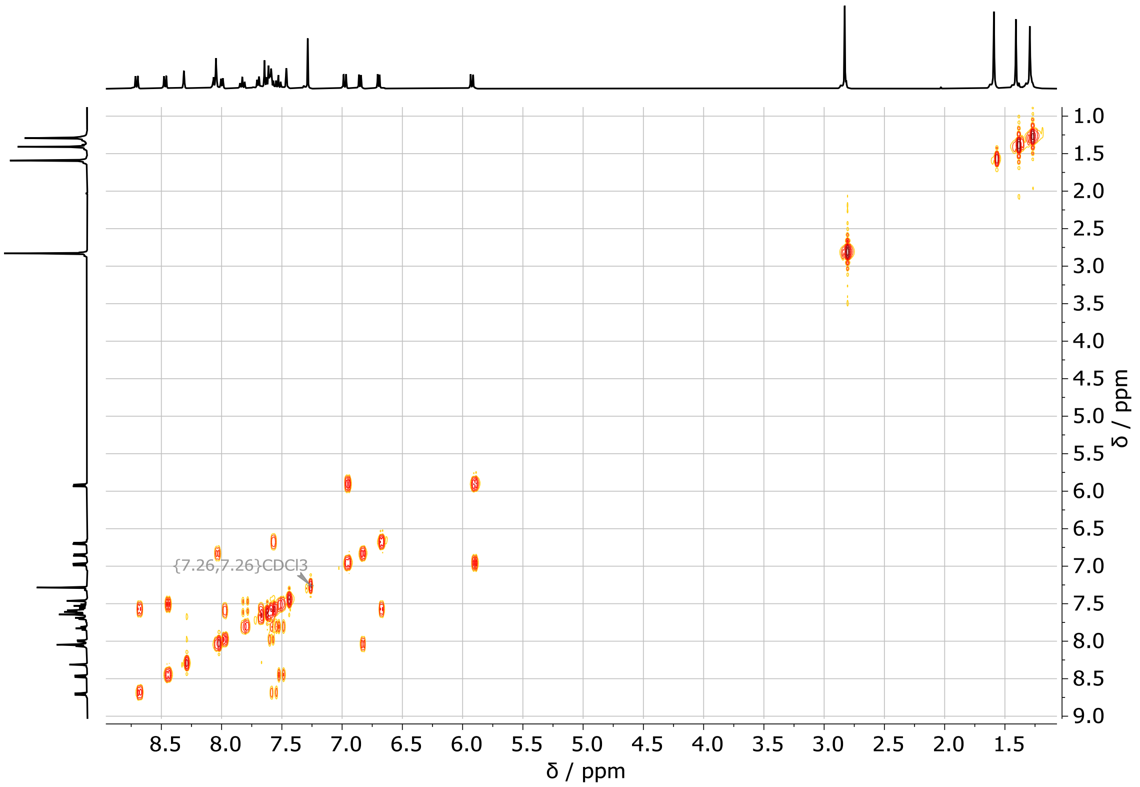

Fig. S25. COSY spectrum (500 MHz, $CDCl_3$) of compound **11**.

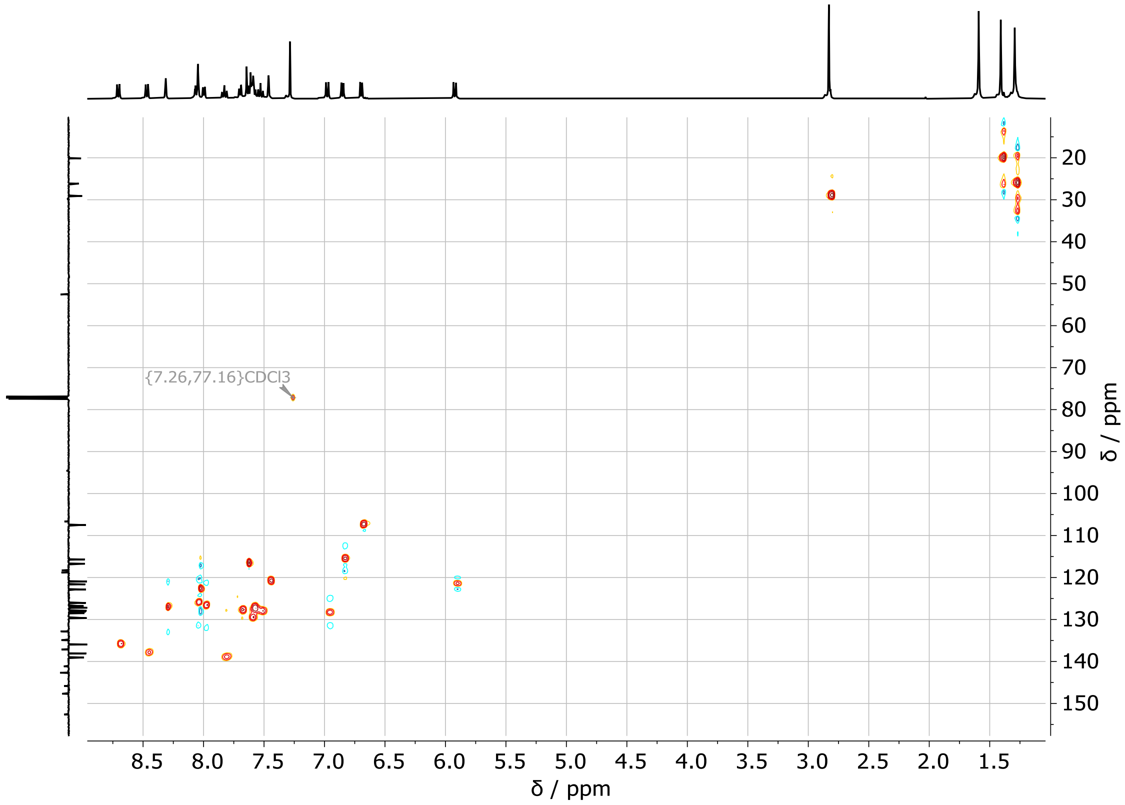

Fig. S26. HSQC spectrum (500/126 MHz, $CDCl_3$) of compound **11**.

• Compound **2**-*para*

2-(4-(1',3',3'-trimethyl-6-nitrospiro[chromene-2,2'-indoline]-5'-yl)phenyl)azulene-1,1(8a*H*)-dicarbonitrile

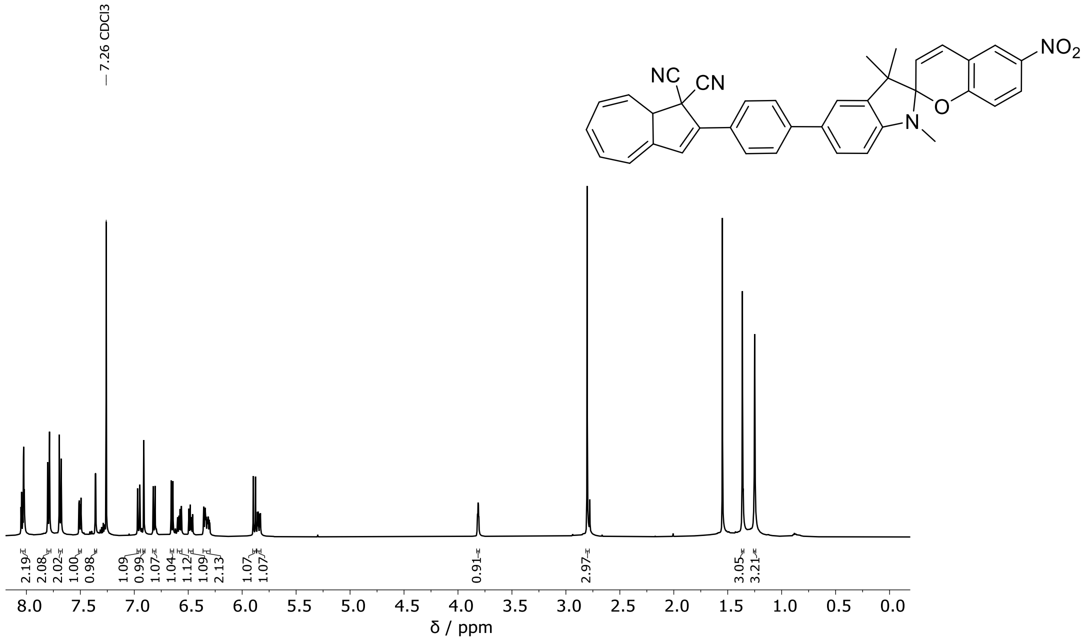

Fig. S27. ^{1}H NMR spectrum (500 MHz, $CDCl_3$) of compound **2**-*para*.

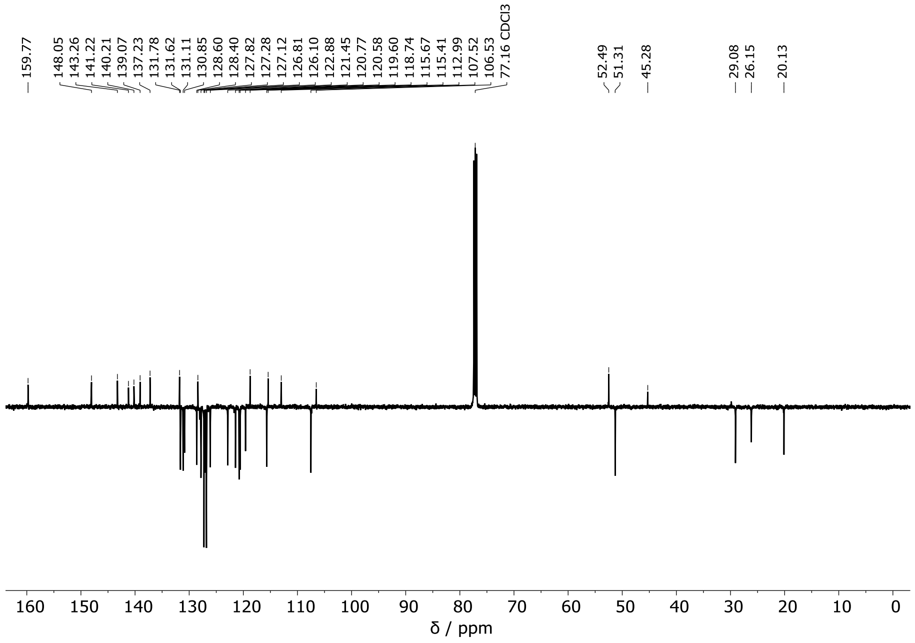

Fig. S28. ^{13}C APT spectrum (126 MHz, $CDCl_3$) of compound **2**-*para*.

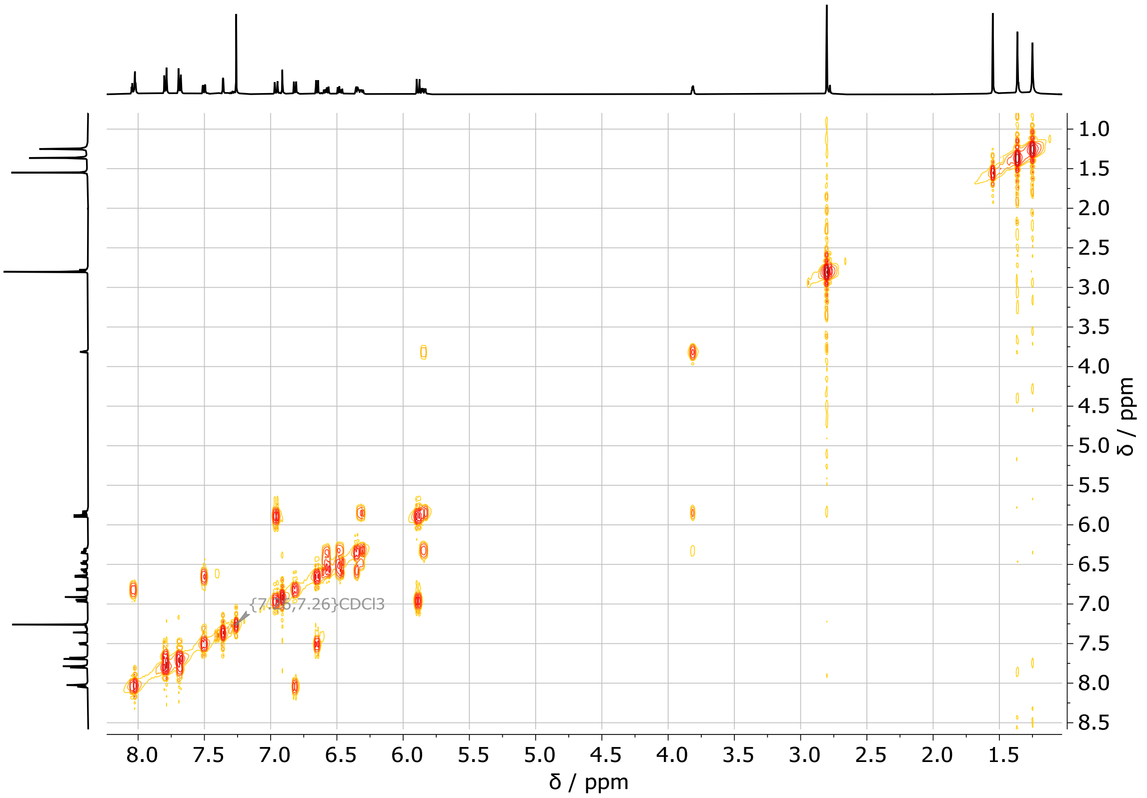

Fig. S29. COSY spectrum (500 MHz, $CDCl_3$) of compound **2**-*para*.

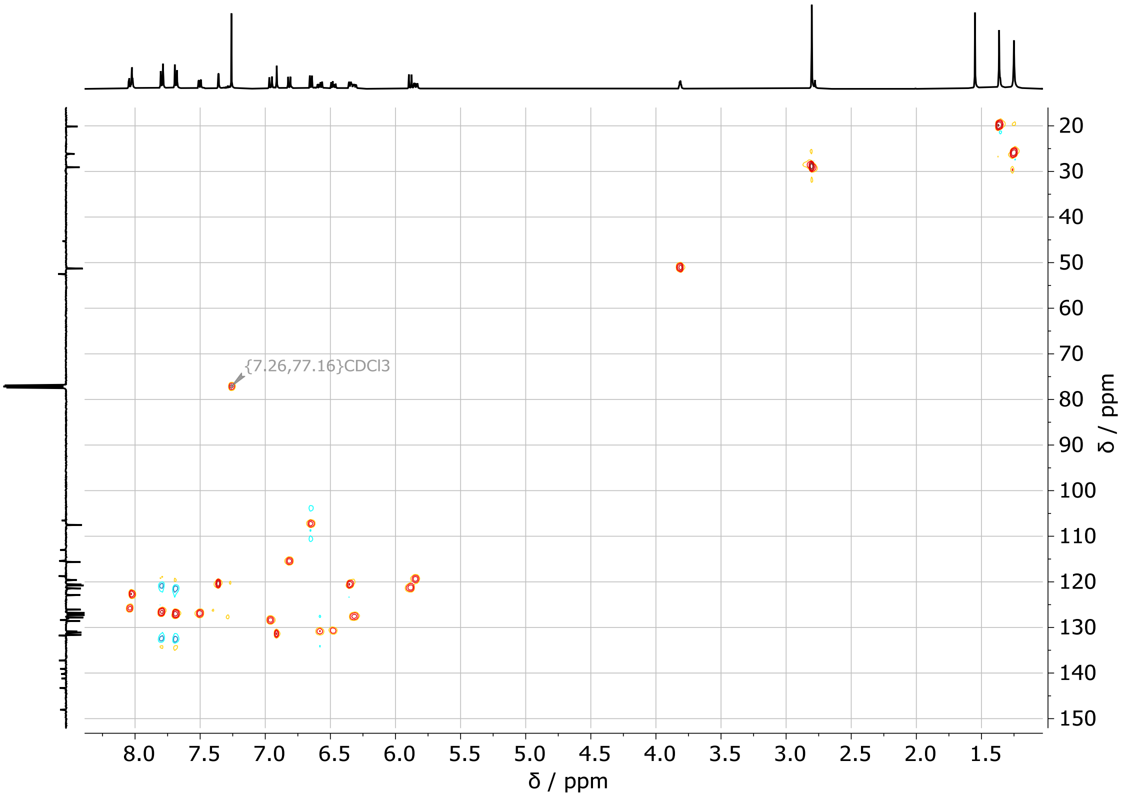

Fig. S30. HSQC spectrum (500/126 MHz, $CDCl_3$) of compound **2**-*para*.

2-(4-(1',3',3'-trimethyl-6-nitrospiro[chromene-2,2'-indoline]-5'-yl)phenyl)azulene-1-carbonitrile (**10**)

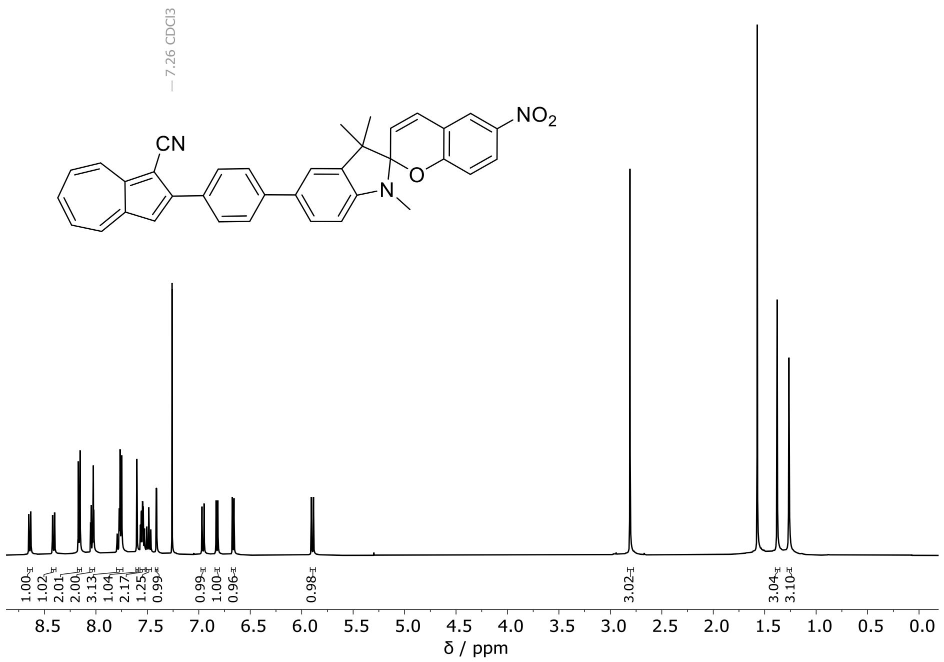

Fig. S31. ^{1}H NMR spectrum (500 MHz, $CDCl_3$) of compound **10**.

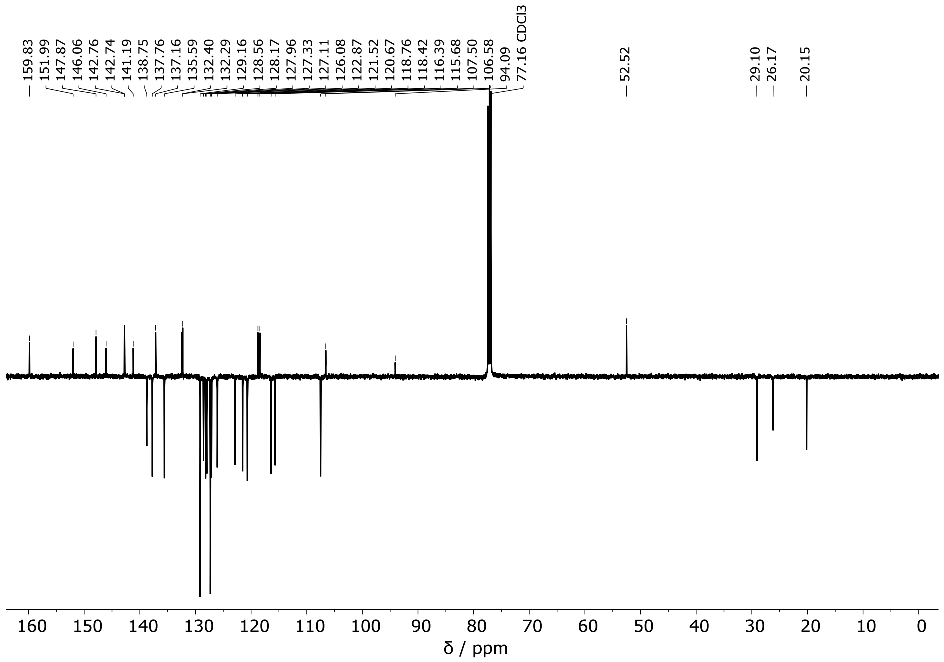

Fig. S32. ^{13}C APT spectrum (126 MHz, $CDCl_3$) of compound **10**.

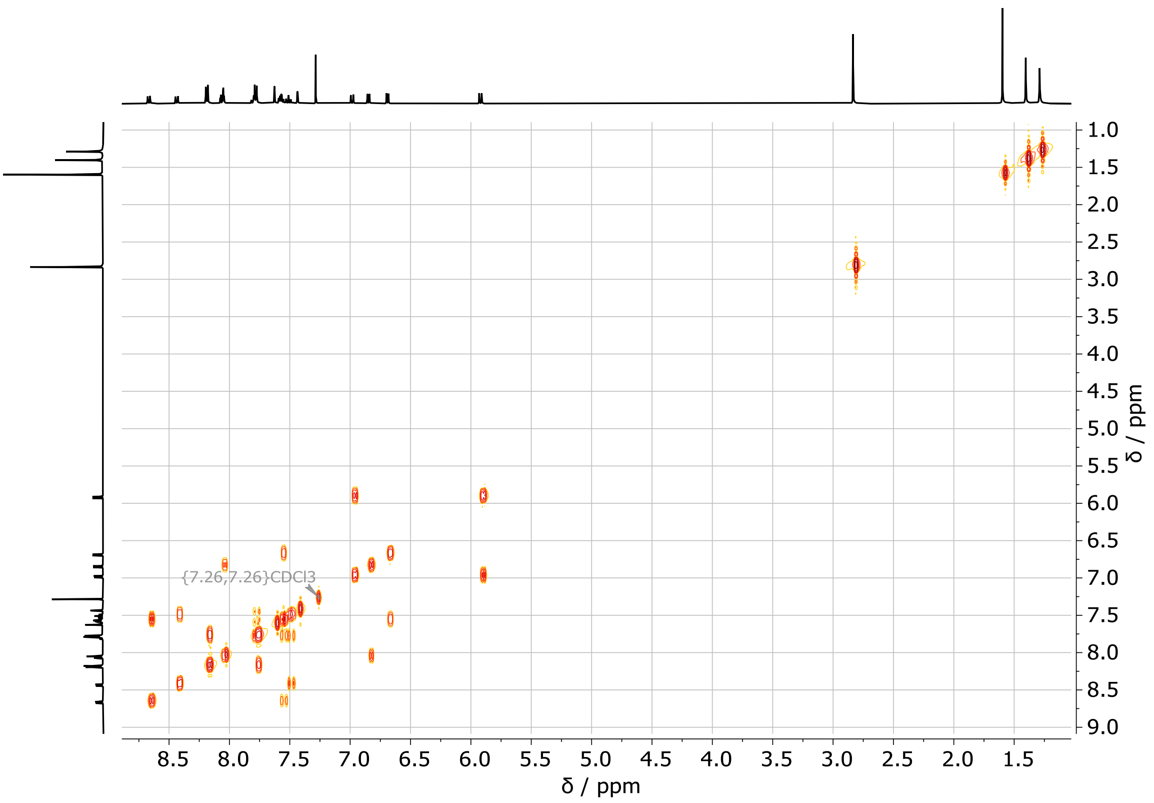

Fig. S33. COSY spectrum (500 MHz, $CDCl_3$) of compound **10**.

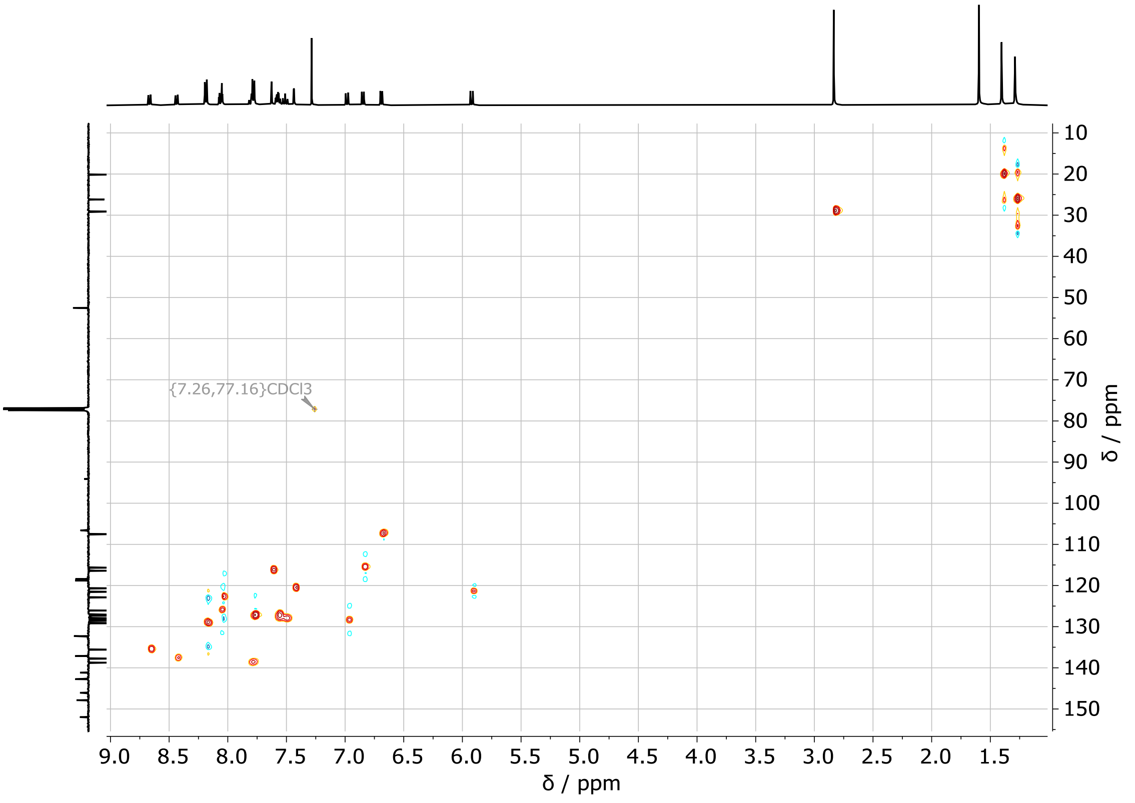

Fig. S34. HSQC spectrum (500/126 MHz, $CDCl_3$) of compound **10**.

NMR spectra after single or multiple stimuli

For NMR switching experiments **2**-*para* and **2**-*meta* were dissolved in CD_3CN (concentration: ~ 3 mM), and cyclohexane was used as an internal standard (δ = 1.44 ppm). Portions of 0.6 mL were used for each NMR sample. Irradiations of NMR samples were carried out in 500 MHz Quartz NMR tubes at 415 nm with a Thorlabs M415L4 with a current at 700 mA. For the protonation of SP, TFA (35 μL) was added to the samples unless stated otherwise.

Compound 2-*meta*

DHA-*cis*MCH form of 2-*meta*

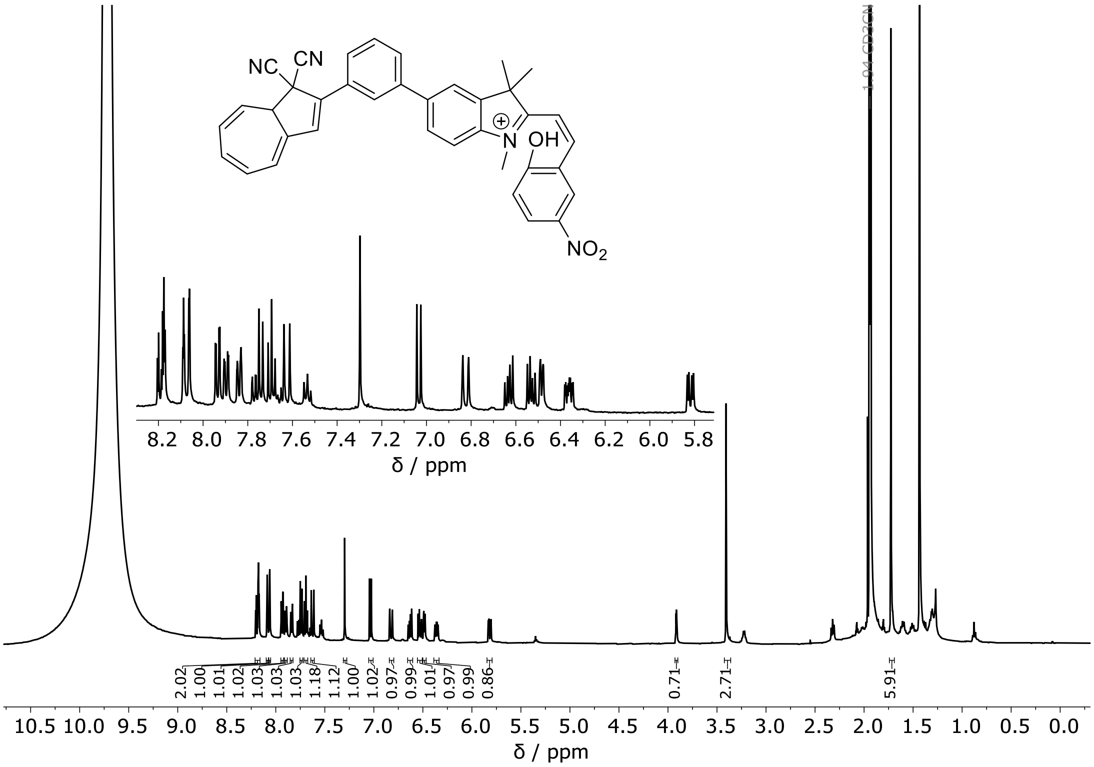

Fig. S35. ^{1}H NMR spectrum (500 MHz, CD_3CN) of **2**-*meta* after addition of TFA (DHA-*cis*MCH form).

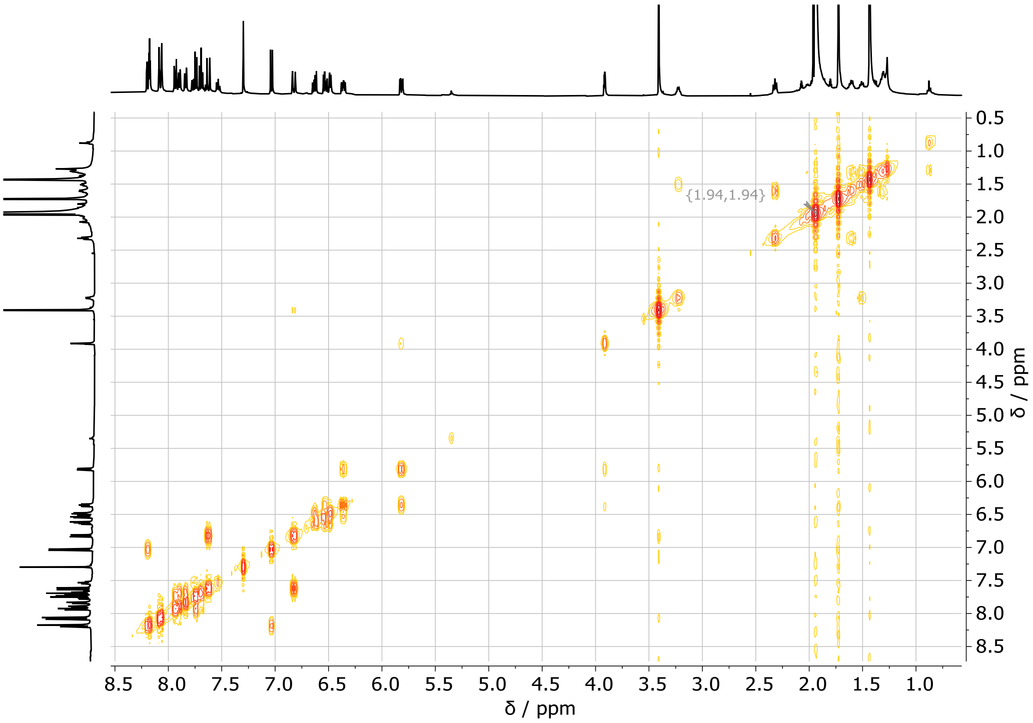

Fig. S36. COSY spectrum (500 MHz, CD_3CN) of **2**-*meta* after addition of TFA (DHA-*cis*MCH form).

DHA-*trans*MCH form of 2-*meta*

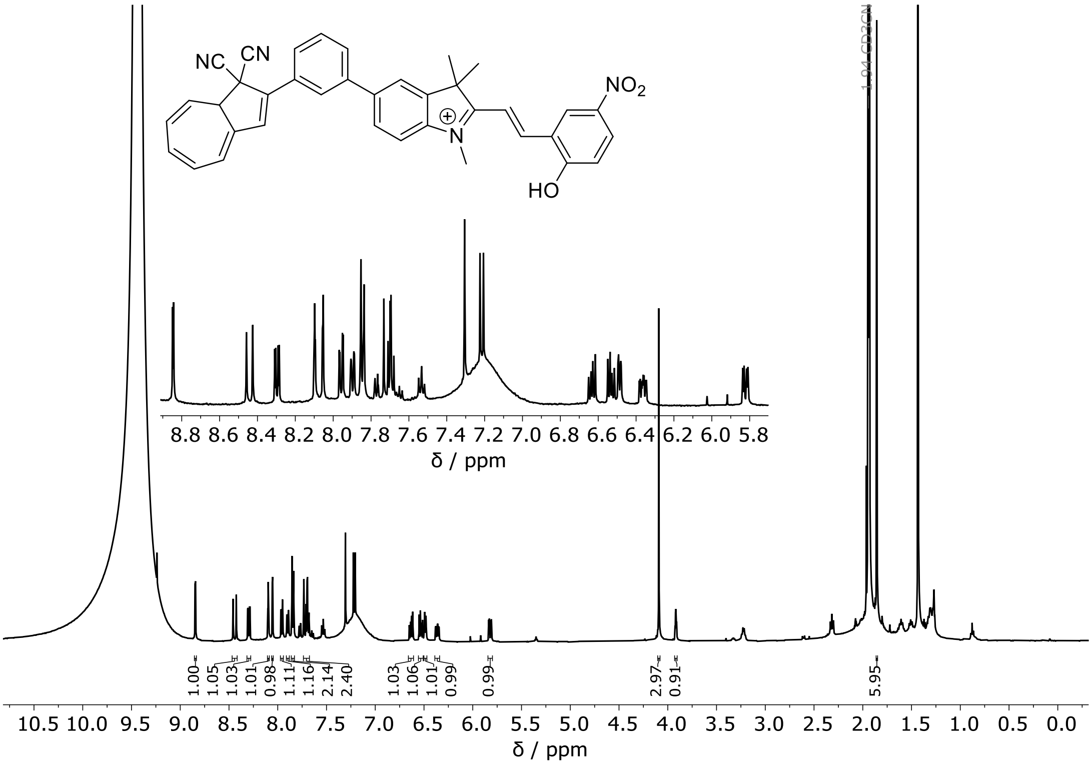

Fig. S37. ^{1}H NMR spectrum (500 MHz, CD_3CN) of **2**-*meta* after addition of TFA and heating the NMR tube at 50 °C for 42 h (DHA-*trans*MCH form).

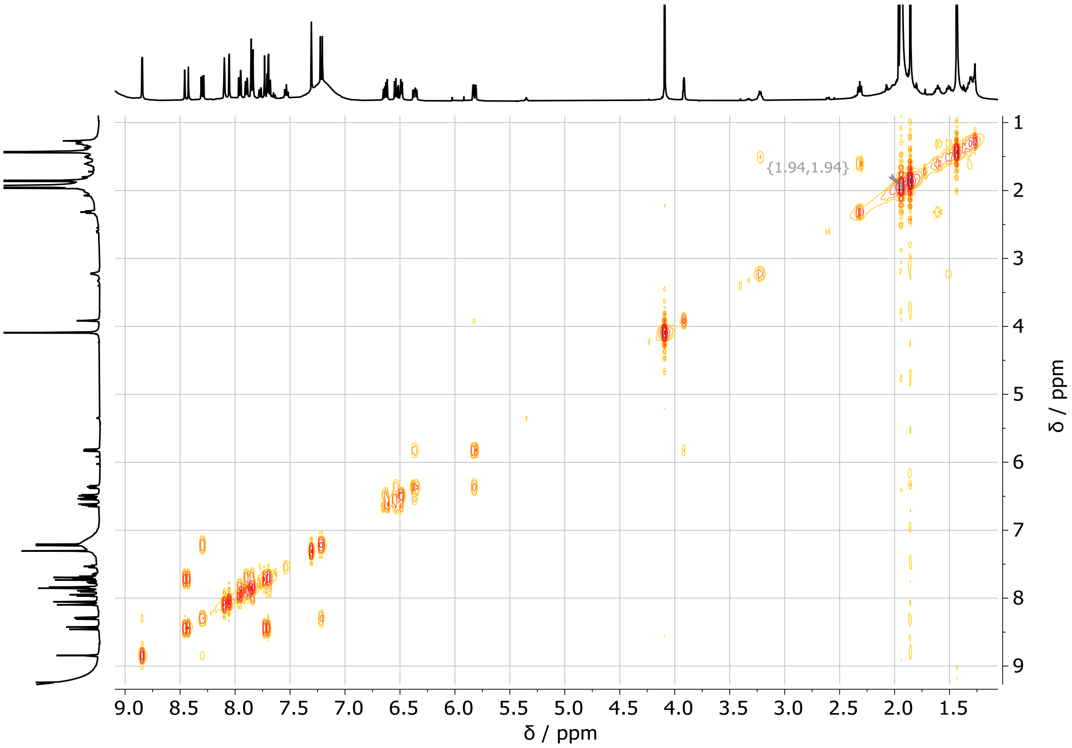

Fig. S38. COSY spectrum (500 MHz, CD_3CN) of **2**-*meta* after addition of TFA and heating the NMR tube at 50 °C for 42 h (DHA-*trans*MCH form).

VHF-SP form of 2-*meta*

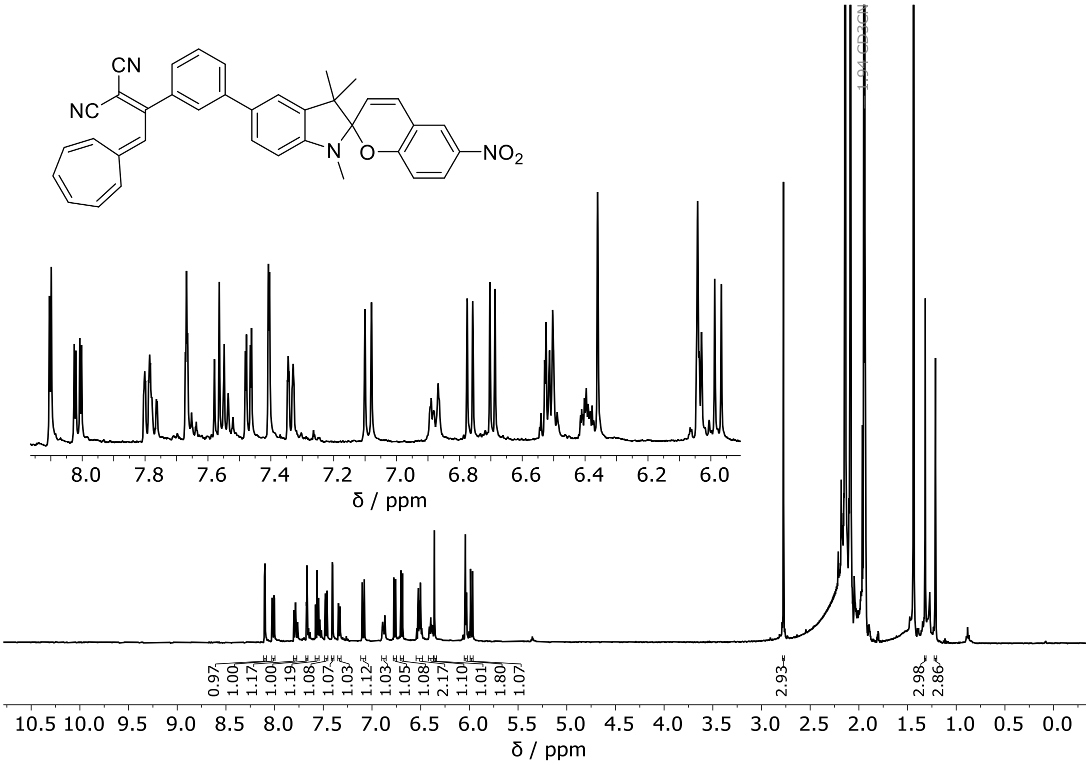

Fig. S39. ^{1}H NMR spectrum (500 MHz, CD3CN) of **2**-*meta* after irradiation at 415 nm for 15 min (VHF-SP form).

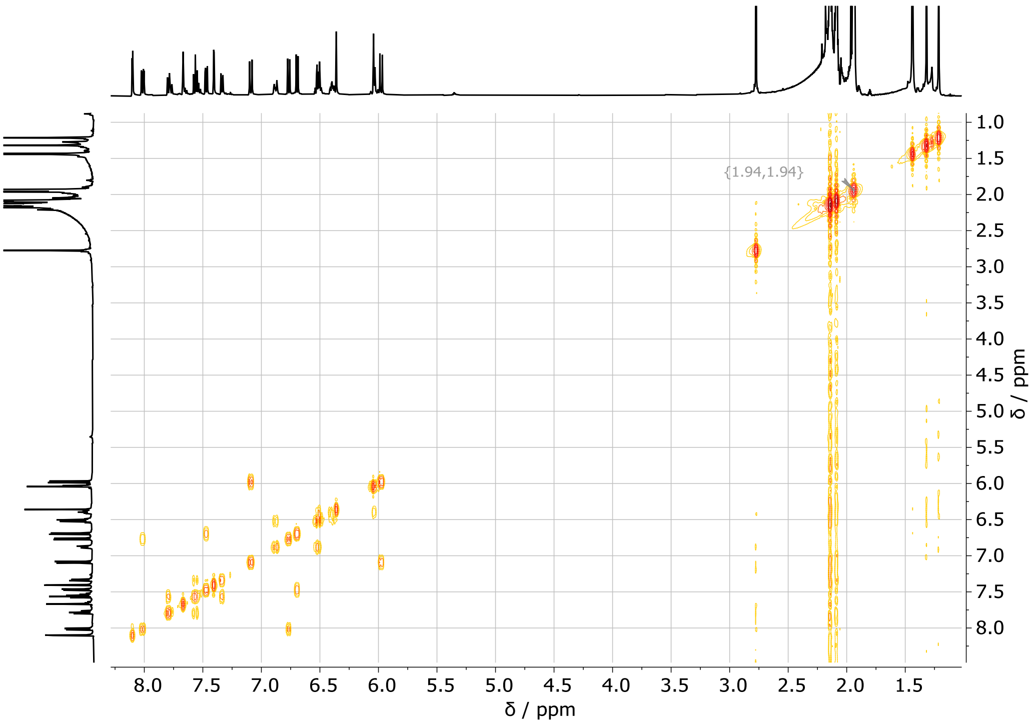

Fig. S40. COSY spectrum (500 MHz, CD3CN) of **2**-*meta* after irradiation at 415 nm for 15 min (VHF-SP form).

VHF-*cis*MCH form of 2-*meta*

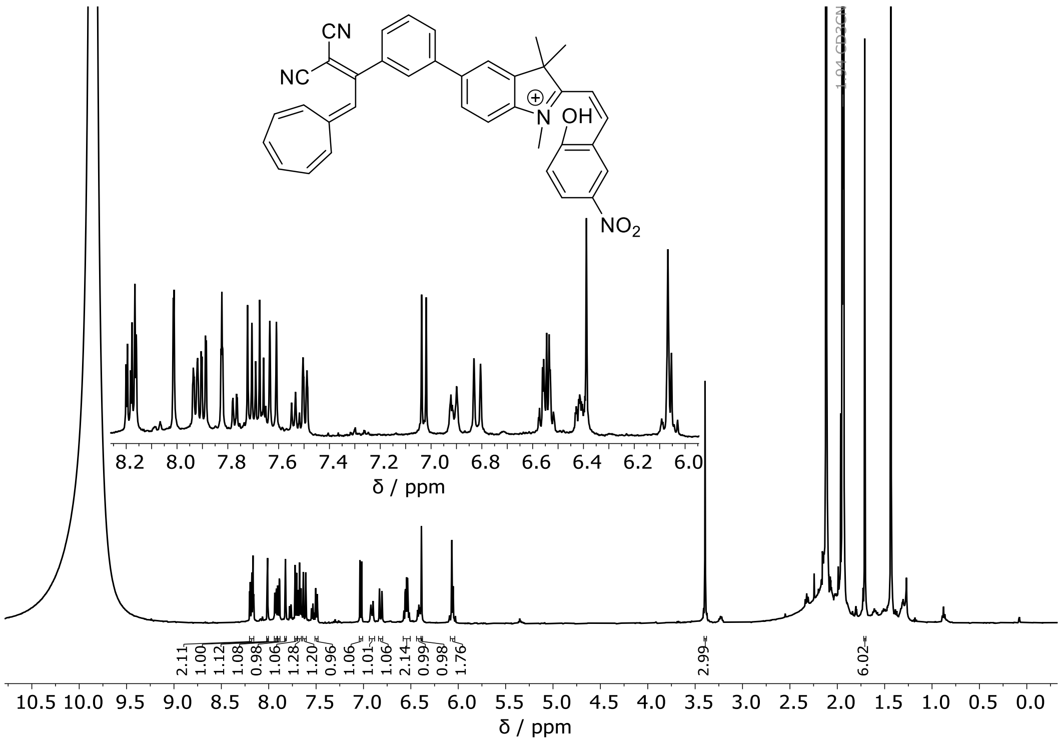

Fig. S41. ^{1}H NMR spectrum (500 MHz, CD_3CN) of **2**-*meta* after irradiation at 415 nm for 21 min and addition of TFA (VHF-*cis*MCH form).

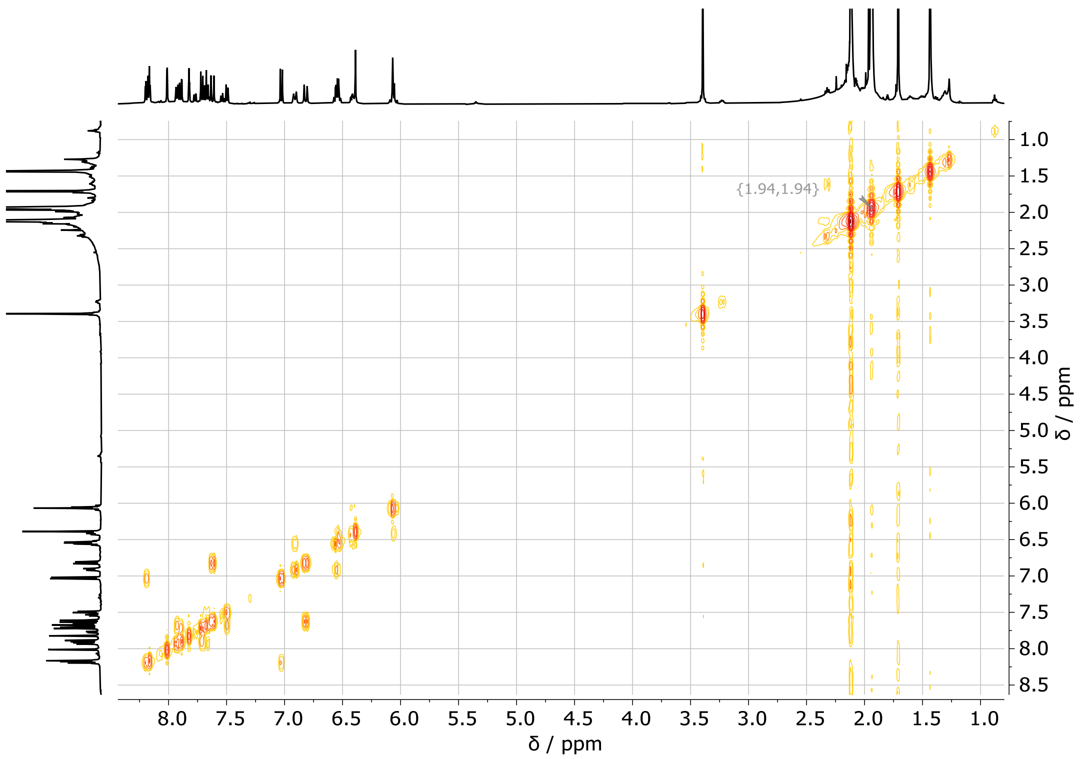

Fig. S42. COSY spectrum (500 MHz, CD_3CN) of **2**-*meta* after irradiation at 415 nm for 21 min and addition of TFA (VHF-*cis*MCH form).

VHF-*trans*MCH form of 2-*meta*

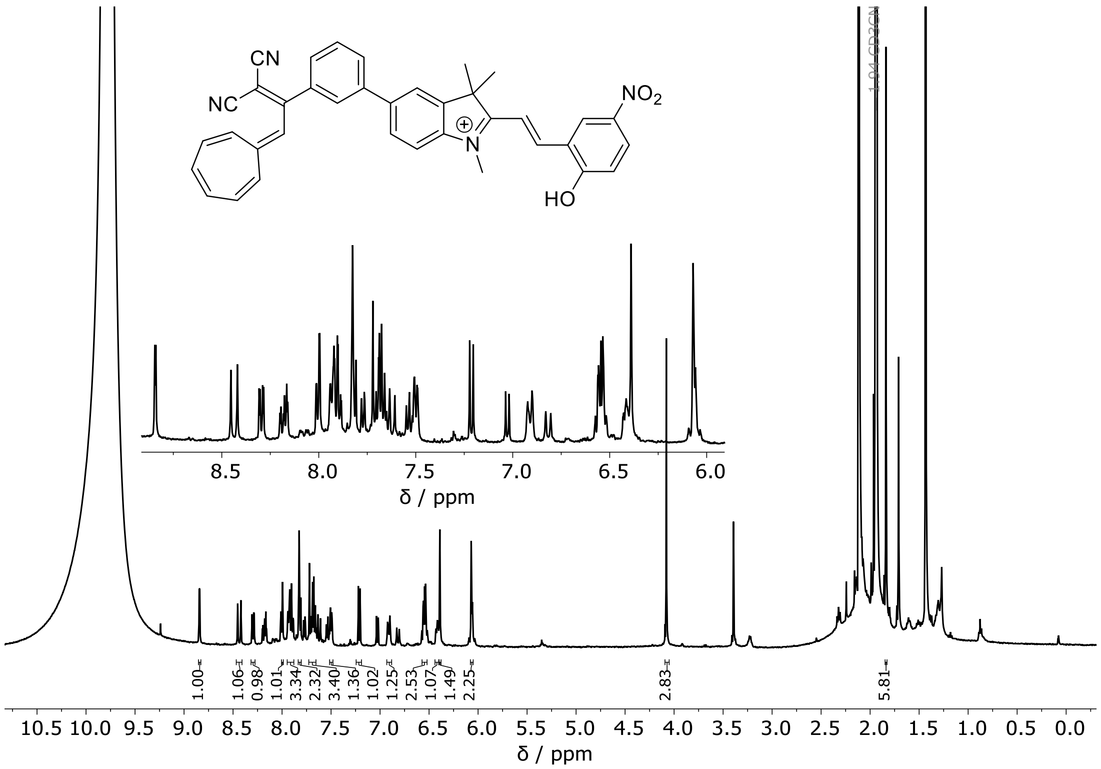

Fig. S43. ^{1}H NMR spectrum (500 MHz, CD_3CN) of **2**-*meta* after irradiation at 415 nm for 21 min, addition of TFA and irradiation again at 415 nm for 15 min (VHF-*trans*MCH form).

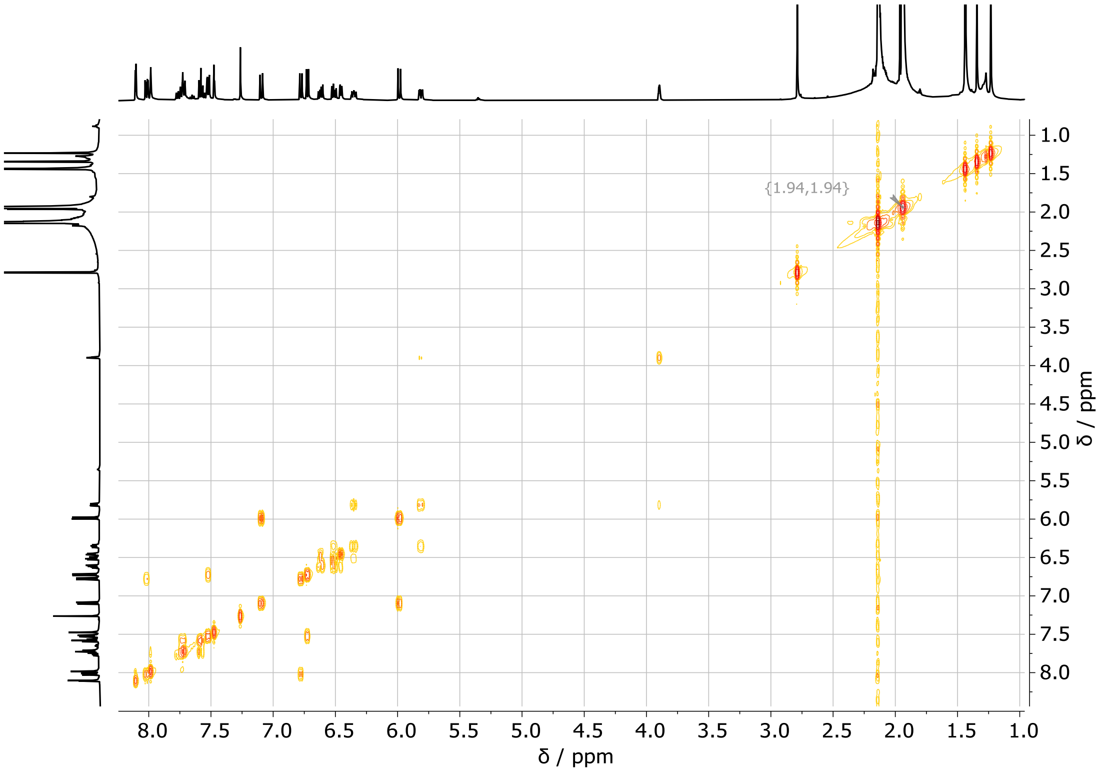

Fig. S44. COSY spectrum (500 MHz, CD_3CN) of **2**-*meta* after irradiation at 415 nm for 21 min, addition of TFA and irradiation again at 415 nm for 15 min (VHF-*trans*MCH form)..

Compound 2-*para*

DHA-*cis*MCH form of 2-*para*

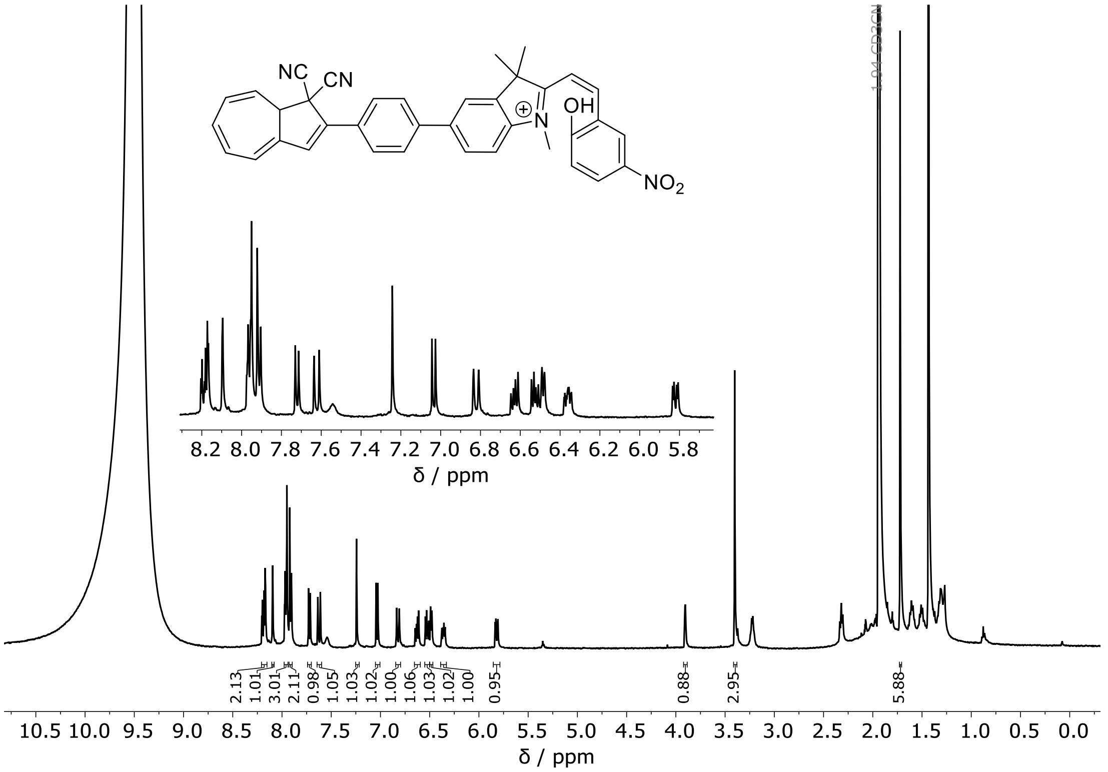

Fig. S45. ^{1}H NMR spectrum (500 MHz, CD_3CN) of **2**-*para* after addition of TFA (DHA-*cis*MCH form).

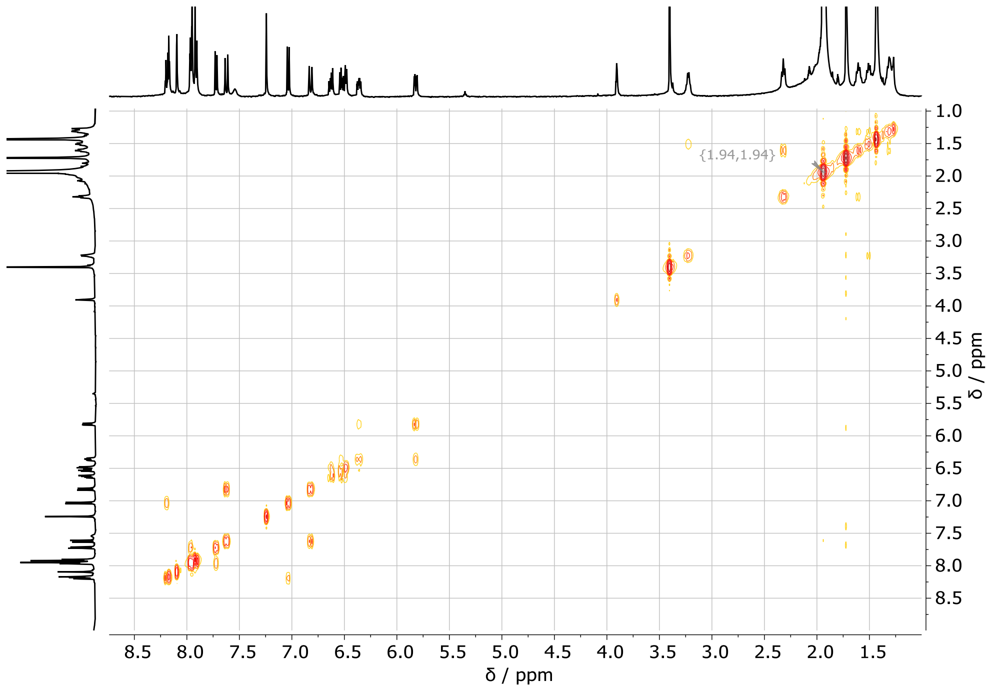

Fig. S46. COSY spectrum (500 MHz, CD_3CN) of **2**-*para* after addition of TFA (DHA-*cis*MCH form).

DHA-*trans*MCH form of 2-*para*

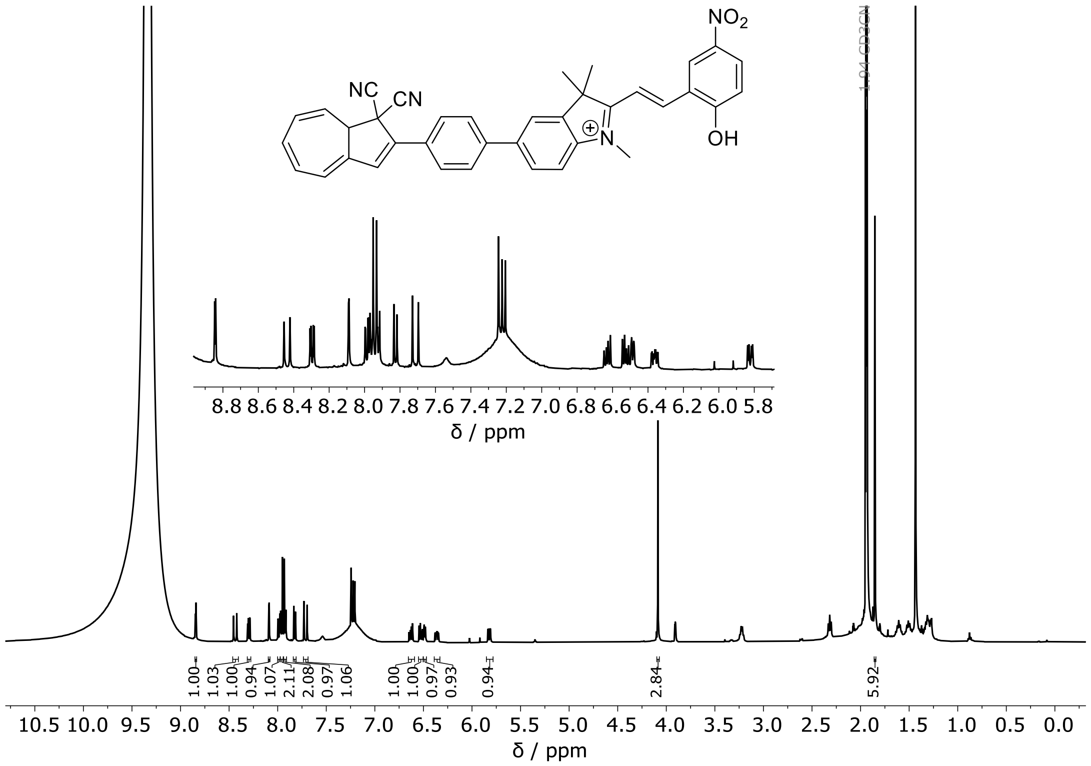

Fig. S47. ^{1}H NMR spectrum (500 MHz, CD_3CN) of **2**-*para* after addition of TFA and heating the NMR tube at 50 °C for 42 h (DHA-*trans*MCH form).

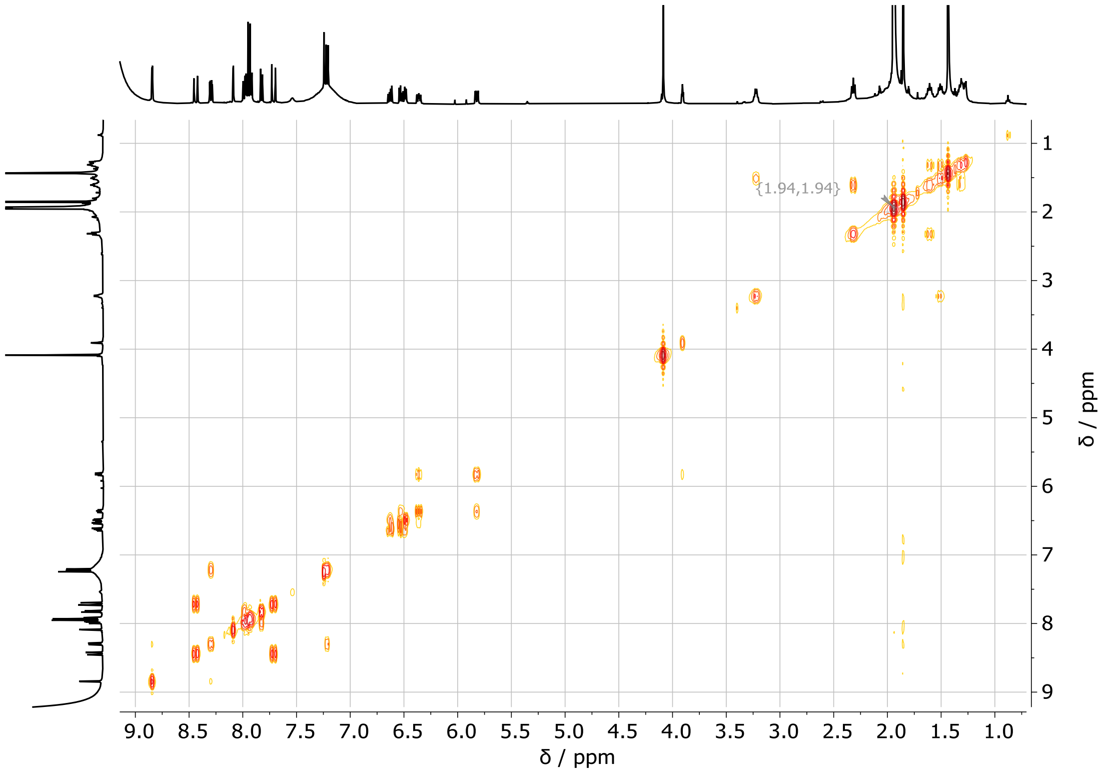

Fig. S48. COSY spectrum (500 MHz, CD_3CN) of **2**-*para* after addition of TFA and heating the NMR tube at 50 °C for 42 h (DHA-*trans*MCH form).

VHF-SP form of 2-*para*

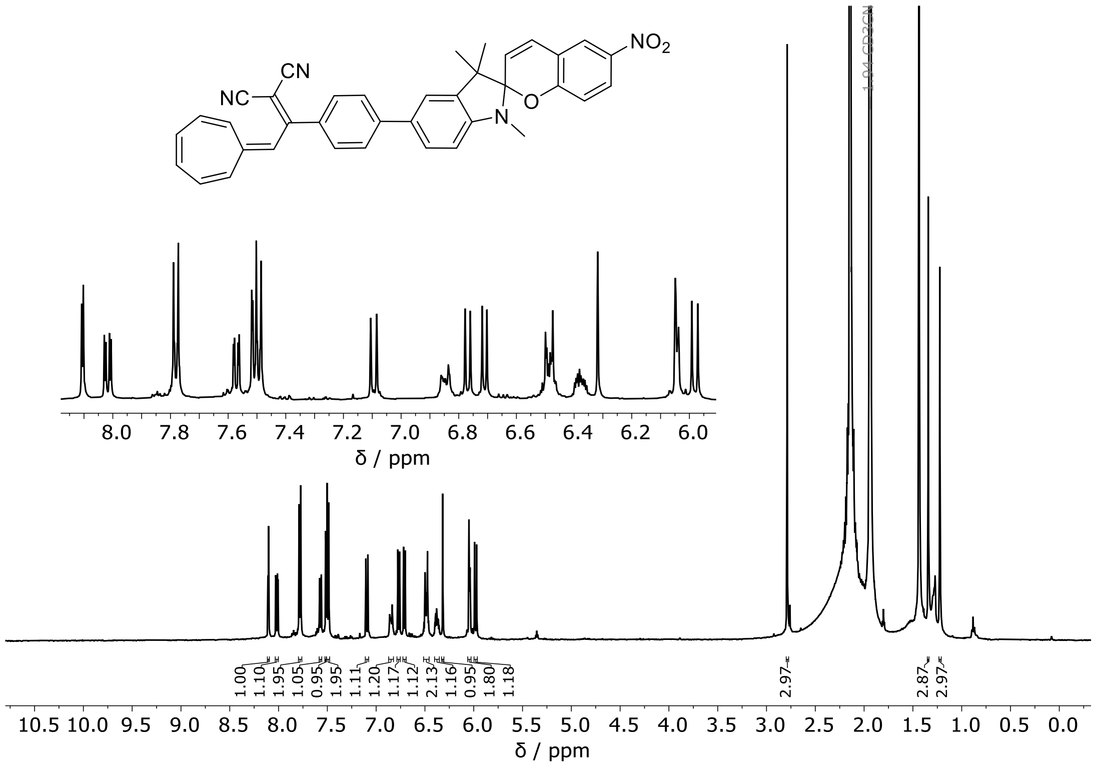

Fig. S49. ^{1}H NMR spectrum (500 MHz, CD_3CN) of **2**-*para* after irradiation at 415 nm for 20 min (VHF-SP form).

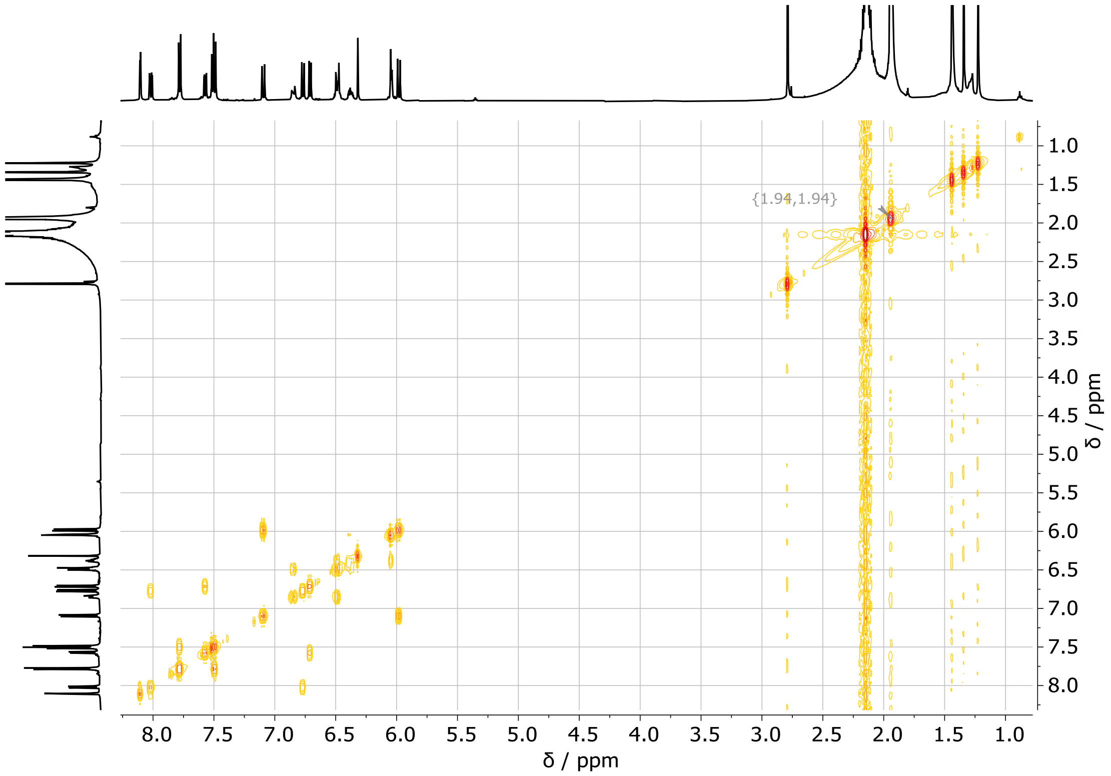

Fig. S50. COSY spectrum (500 MHz, CD_3CN) of **2**-*para* after irradiation at 415 nm for 20 min (VHF-SP form).

VHF-*cis*MCH form of 2-*para*

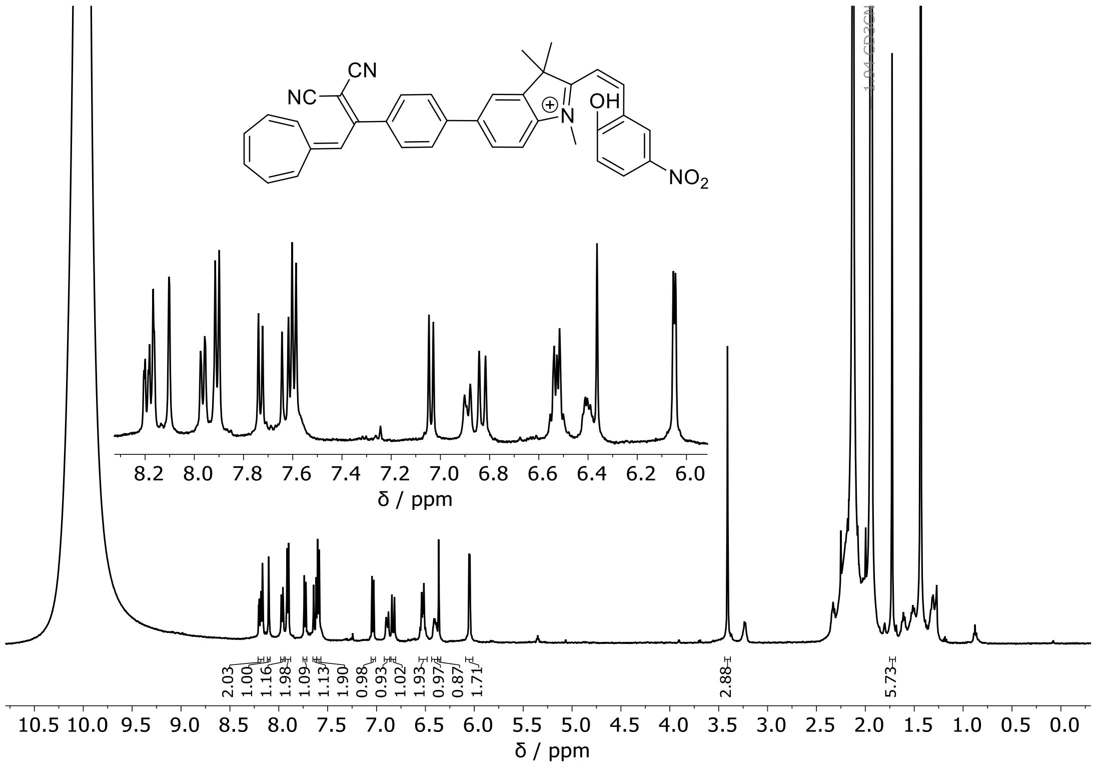

Fig. S51. ^{1}H NMR spectrum (500 MHz, CD_3CN) of **2**-*para* after irradiation at 415 nm for 17 min and addition of TFA (VHF-*cis*MCH form).

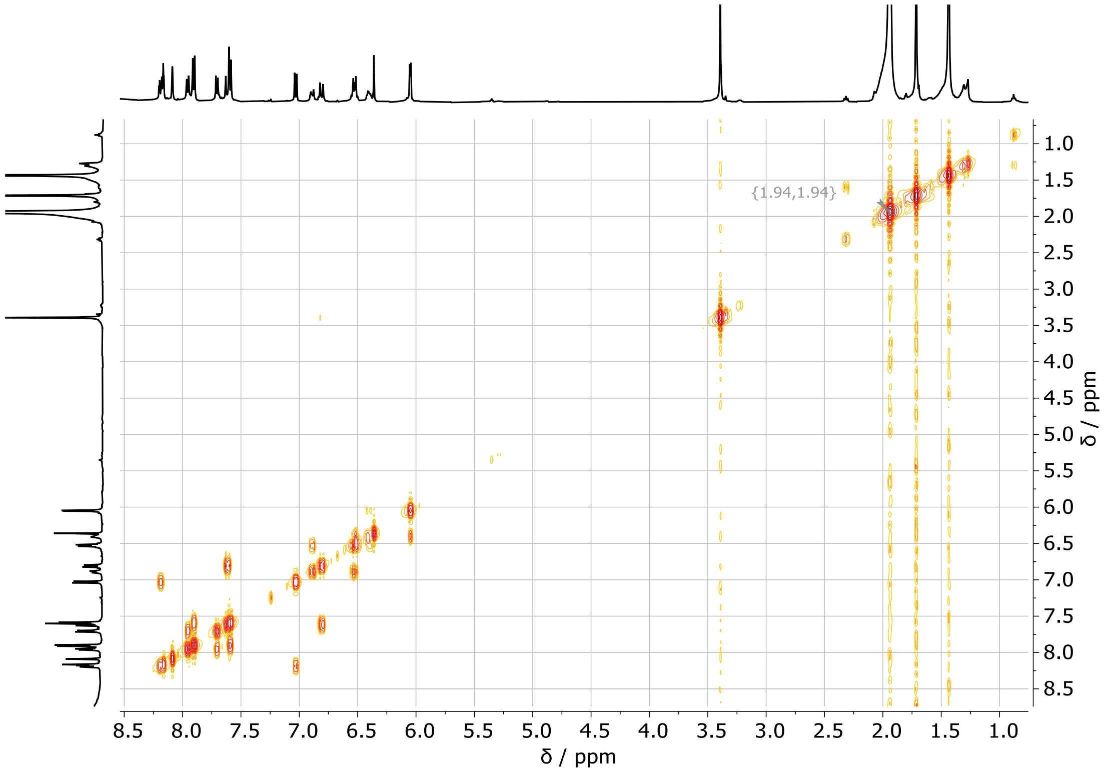

Fig. S52. COSY spectrum (500 MHz, CD_3CN) **2**-*para* after irradiation at 415 nm for 17 min and addition of TFA (VHF-*cis*MCH form).

VHF-*trans*MCH form of 2-*para*

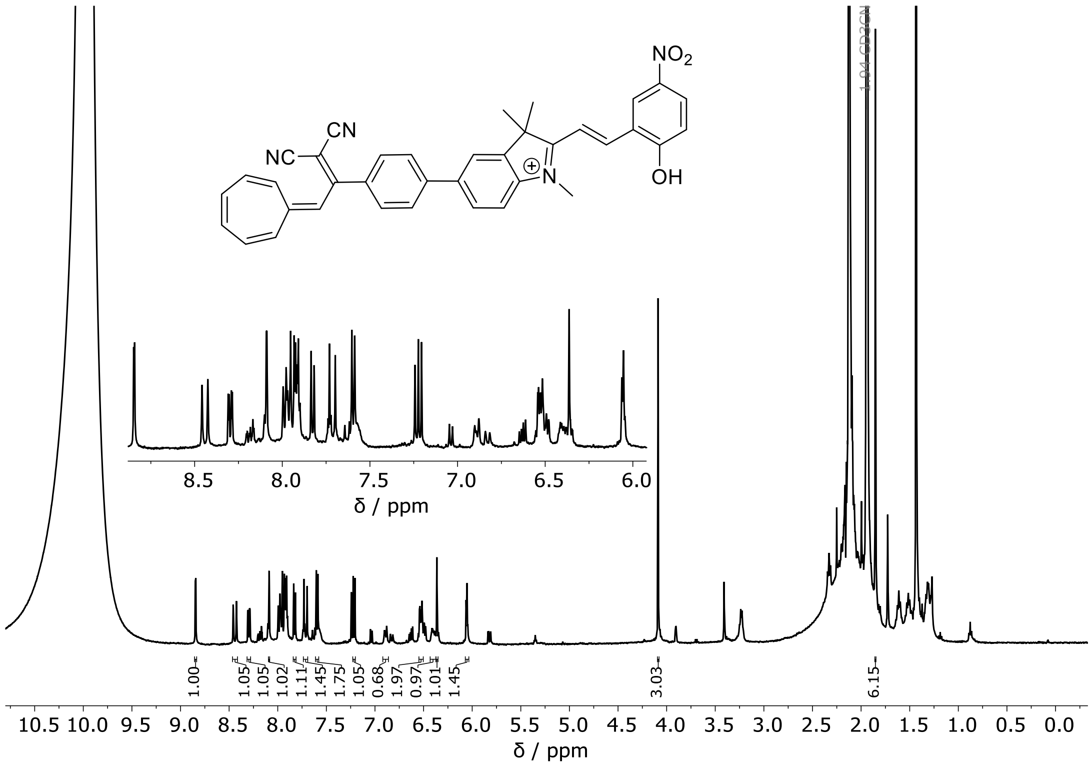

Fig. S53. ^{1}H NMR spectrum (500 MHz, CD_3CN) of **2**-*para* after irradiation at 415 nm for 17 min, addition of TFA and irradiation again at 415 nm for 42 min (VHF-*trans*MCH form).

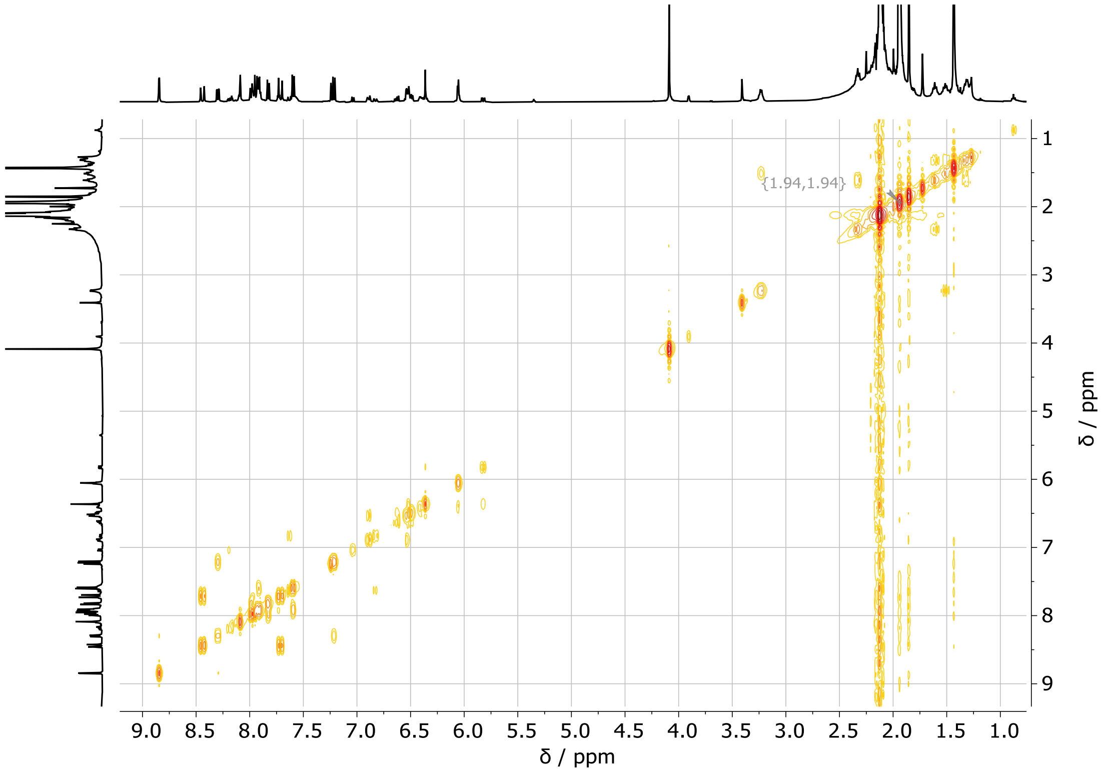

Fig. S54. COSY spectrum (500 MHz, CD_3CN) of **2**-*para* after irradiation at 415 nm for 17 min, addition of TFA and irradiation again at 415 nm for 42 min (VHF-*trans*MCH form).

Chiral HPLC analysis

Compound 2-*meta*

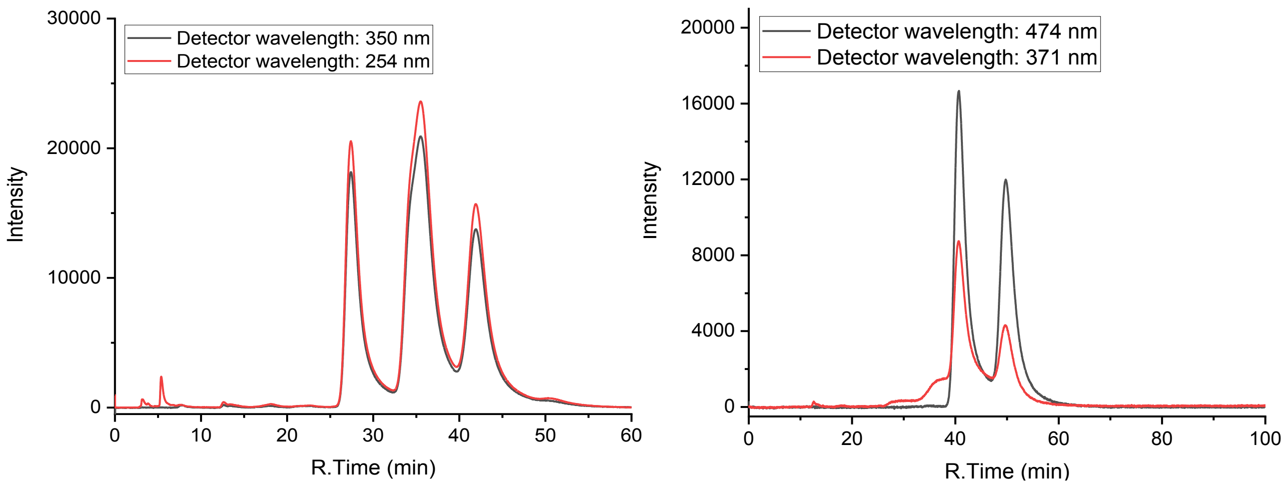

Peak	R. time (min)	Area (350 nm)
1	27	$3.8 \cdot 10^4$
2	36	$7.3 \cdot 10^4$
3	42	$4.0 \cdot 10^4$

Peak	R. time (min)
1	41
2	50

Fig. S55. Left: Chromatogram of compound **2**-*meta* (DHA-SP forms) from chiral HPLC detected at wavelengths 350 (black line) and 254 nm (red line). Right: Chromatogram of compound **2**-*meta* after irradiation at 415 nm for 24 min (200 mA) (VHF-SP forms) detected at wavelengths 474 (black line) and 371 nm (red line). Column: 5-AmyCoat 4.6x250 mm. Eluent: 10% *i*PrOH/heptane. Flowrate: 1 mL/min.

Compound 2-*para*

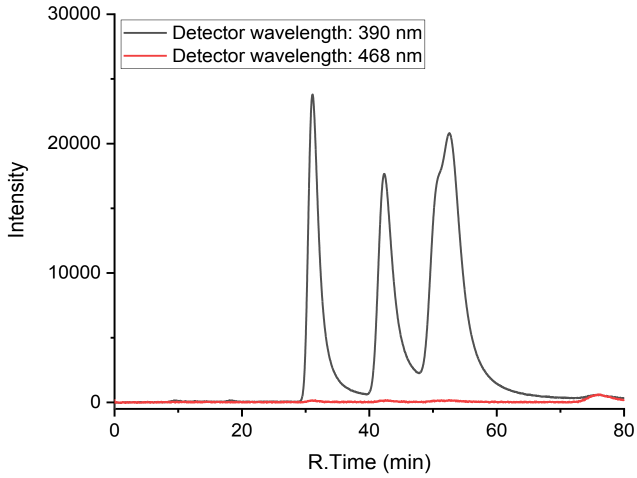

Peak	R. time (min)	Area (390 nm)
1	31	$5.7 \cdot 10^4$
2	42	$5.7 \cdot 10^4$
3	53	$11.6 \cdot 10^4$

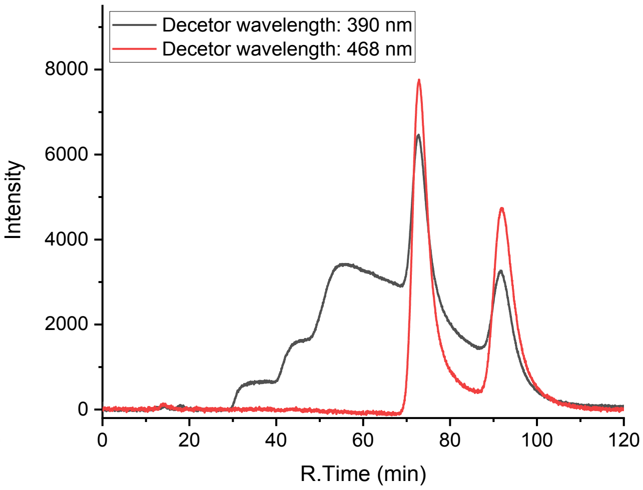

Peak	R. time (min)
1	73
2	92

Fig. S56. Left: Chromatogram of compound **2**-*para* (DHA-SP forms) from chiral HPLC detected at wavelengths 390 (black line) and 468 nm (red line). Right: Chromatogram after irradiation of compound **2**-*para* at 415 nm for 20 min (10 min at 100 mA, 10 min at 200 mA) (VHF-SP forms) detected at wavelengths 390 (black line) and 468 nm (red line). Column: 5-AmyCoat 4.6x250 mm. Eluent: 10% *i*PrOH/heptane. Flowrate: 1 mL/min.

Compound 1-*ortho*

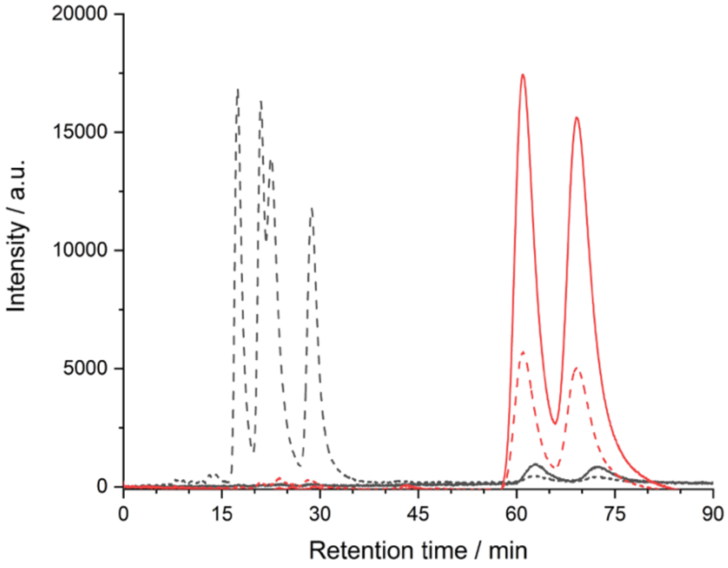

Fig. S57. Superimposed Chromatograms of compound **1**-*ortho* before irradiation (DHA-SP forms, black) and after irradiation at 415 nm (VHF-SP forms, red) from chiral HPLC detected at wavelengths 317 nm (dashed line) and 477 nm (full line). Column: 5-AmyCoat 4.6x250 mm. Eluent: 10% *i*PrOH/heptane. Flowrate: 1 mL/min.

UV-Vis absorption spectra and switching studies

UV-Vis absorption spectra were recorded on a Varian Cary 50 Bio UV-vis spectrophotometer, equipped with a Varian Cary Single Cell Peltier Accessory for temperature control, scanning between 800 and 200 nm. UV-Vis absorption spectra of compound **1**-*ortho* were recorded on a Cary 60 UV-vis Spectrophotometer. All spectra were recorded in MeCN (HPLC grade) at 25 °C in quartz cuvettes with a 10-mm light path. The irradiation experiments were carried out using mounted LEDs from Thorlabs controlled by a Thorlabs DC4100 4-channel LED driver equipped with a DC4100-HUB connector. The samples were irradiated at three different wavelengths: at 340 nm with a Thorlabs M340L4 (optical power: 53 mW, maximal current: 700 mA, bandwidth 11 nm) with a current at 500 mA unless stated otherwise; at 365 nm with a Thorlabs M365L3 (optical power: 880 mW, maximal current: 1000 mA, bandwidth 9 nm) with a current at 250 mA unless stated otherwise; and at 415 nm with a Thorlabs M415L4 (optical power: 1550 mW, maximal current: 1500 mA, bandwidth 14 nm) with a current at 50 mA unless stated otherwise.

Stock solutions were prepared in MeCN, previously degassed with nitrogen. For the protonation of SP, TFA (100 µL) was added to 2.9 mL of the sample solutions. Deprotonation was performed by adding Et_3N (500 µL) to 2.5 mL of the sample solutions. The absorption and switching experiments were performed in a dark room to minimize interference from external light.

Absorption characteristics

	**λ_{max} (nm)**	**ε (10^3 $M^{-1}cm^{-1}$)**
**1**-*meta*	267	34.7 ± 1.2
	339	40.8 ± 1.4
**1**-*ortho*	267	29.4 ± 0.6
	327	36.9 ± 0.4
Spiropyran **6**	270	35.2 ± 0.2
	338	10.3 ± 0.1
**2**-*meta*	270	38.3 ± 0.1
	309	26.7 ± 0.03
	349	28.6 ± 0.08
meta-azulene **11**	223	45.3 ± 0.2
	273	38.6 ± 0.2
	318	78.8 ± 0.4
**2**-*para*	268	31.0 ± 0.3
	387	40.5 ± 0.3
para-azulene **10**	306	62.9 ± 0.2
	347	35.7 ± 0.2
	414	23.6 ± 0.07

Table S1. Longest wavelength absorption maxima and the corresponding extinction coefficients of **1**-*meta,* **1**-*ortho,* spiropyran **6**, **2**-*meta*, *meta*-azulene **11**, **2**-*para*, *para*-azulene **10**.

Compound **1**-*meta*

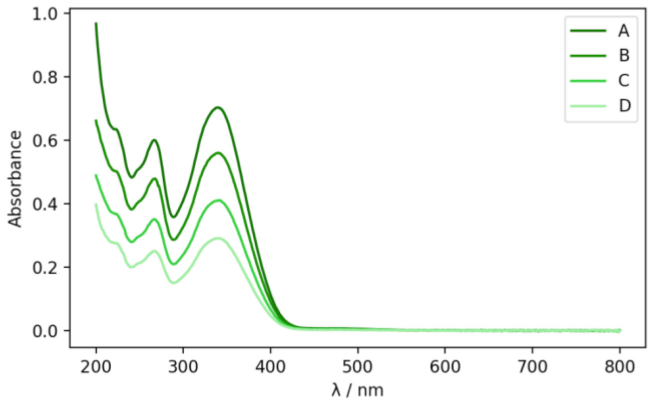

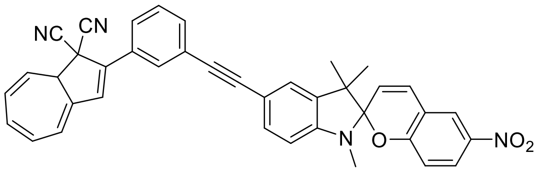

Fig. S58. Dilution row of **1**-*meta* in MeCN at 25 °C. Concentrations: $1.70 \cdot 10^{-5}$ M, $1.36 \cdot 10^{-5}$ M, $1.02 \cdot 10^{-5}$ M, and $0.68 \cdot 10^{-5}$ M.

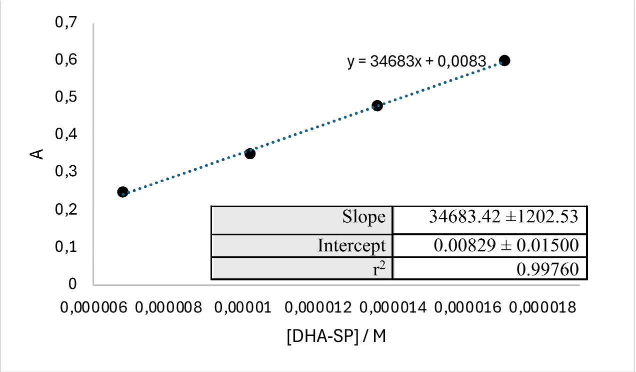

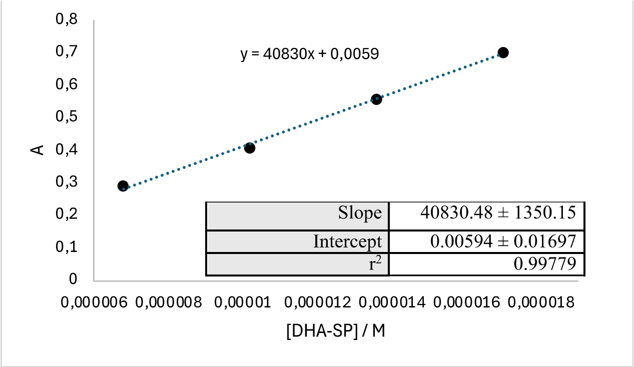

Fig. S59. Left: Absorption of **1**-*meta* at the maximum at 267 nm plotted vs. the concentration and fitting of the data to a linear function. ε at 267 nm = $(34.7 \pm 1.2) \cdot 10^3$ $M^{-1}cm^{-1}$. Right: Absorption of **1**-*meta* at the maximum at 339 nm plotted vs. the concentration and fitting of the data to a linear function. ε at 339 nm = $(40.8 \pm 1.4) \cdot 10^3$ $M^{-1}cm^{-1}$.

Compound **1**-*ortho*

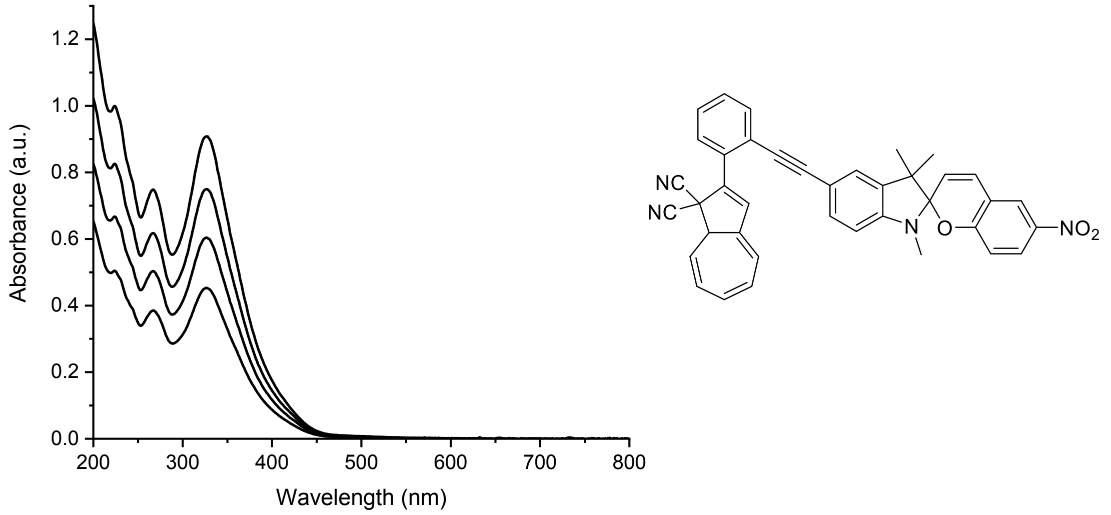

Fig. S60. Dilution row of **1**-*ortho* in MeCN at 25 °C. Concentrations: $2.46\cdot10^{-5}$ M, $2.05\cdot10^{-5}$ M, $1.64\cdot10^{-5}$ M, and $1.23\cdot10^{-5}$ M.

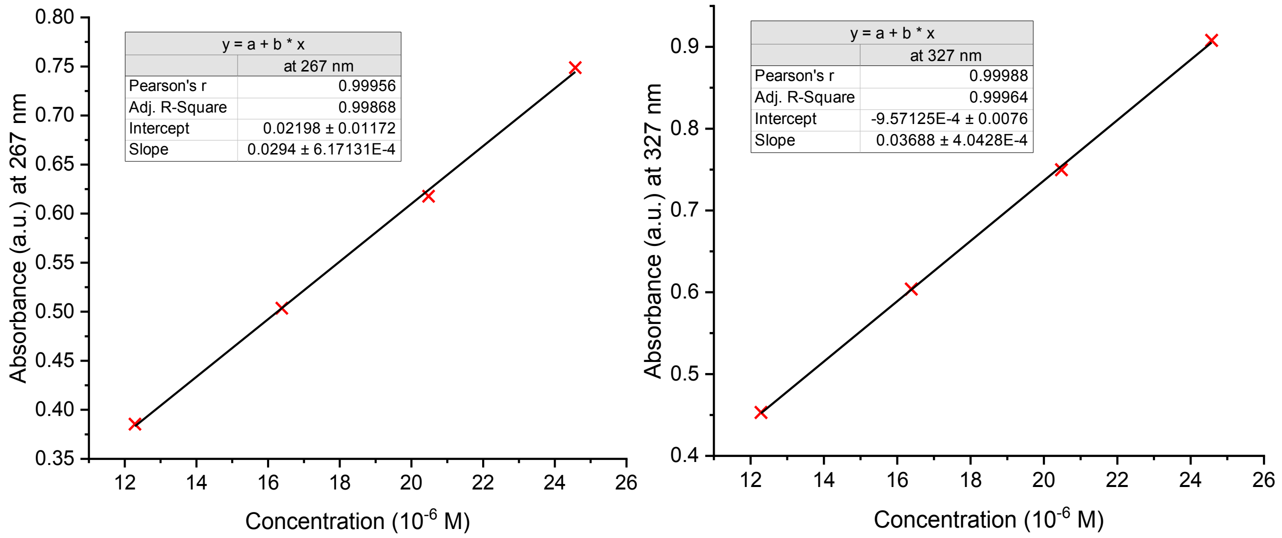

Fig. S61. Left: Absorption of **1**-*ortho* at the maximum at 267 nm (red crosses) plotted vs. the concentration and fitting of the data to a linear function (black line). ε at 267 nm = $(29.4 \pm 0.6)\cdot10^{3}$ $M^{-1}cm^{-1}$. Right: Absorption of **1**-*ortho* at the maximum at 327 nm (red crosses) plotted vs. the concentration and fitting of the data to a linear function (black line). ε at 327 nm = $(36.9 \pm 0.4)\cdot10^{3}$ $M^{-1}cm^{-1}$.

Spiropyran compound **6**

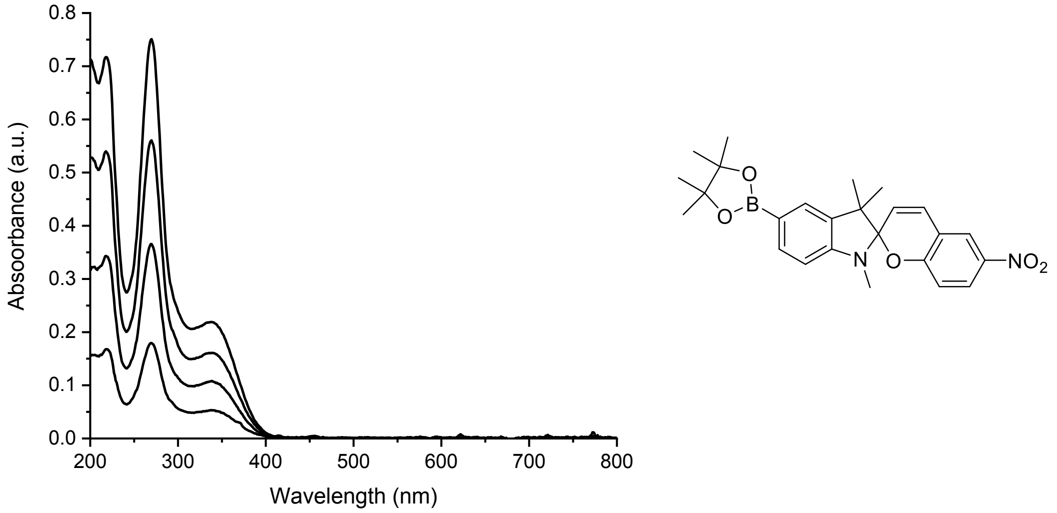

Fig. S62. Dilution row of **6** in MeCN at 25 °C. Concentrations $2.14{\cdot}10^{-5}$ M, $1.61{\cdot}10^{-5}$ M, $1.07{\cdot}10^{-5}$ M, and $5.35{\cdot}10^{-6}$ M.

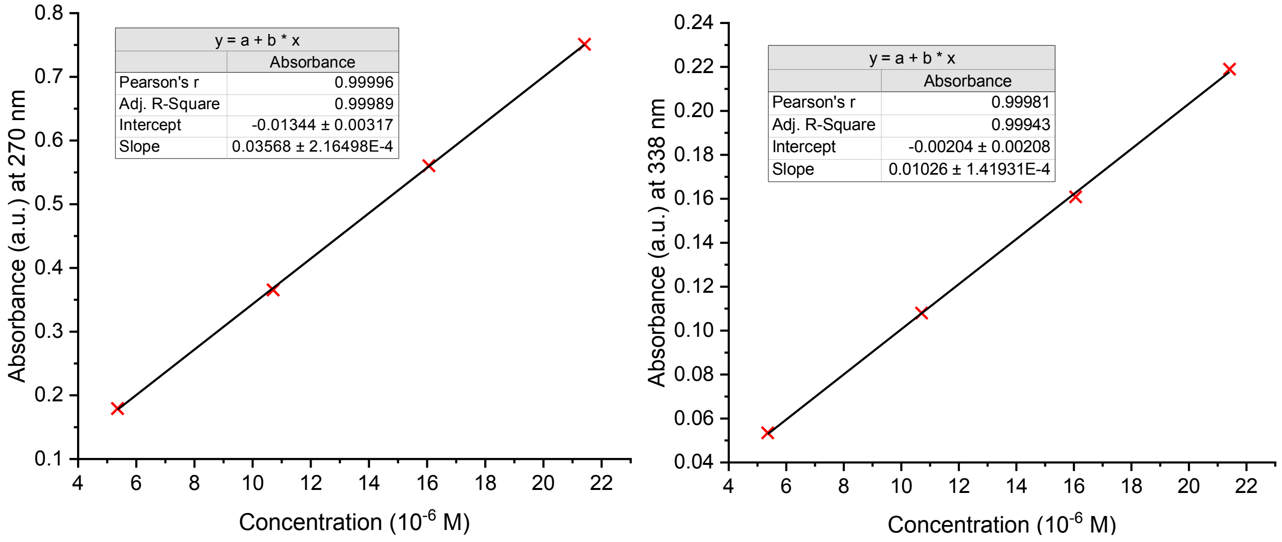

Fig. S63. Left: Absorption of **6** at the maximum at 270 nm (red crosses) plotted vs. the concentration and fitting of the data to a linear function (black line). ε at 270 nm = $(35.7 \pm 0.2){\cdot}10^3$ $M^{-1}cm^{-1}$. Right: Absorption of **6** at the maximum at 338 nm (red crosses) plotted vs. the concentration and fitting of the data to a linear function (black line). ε at 338 nm = $(10.3 \pm 0.1){\cdot}10^3$ $M^{-1}cm^{-1}$.

Compound **2**-*meta*

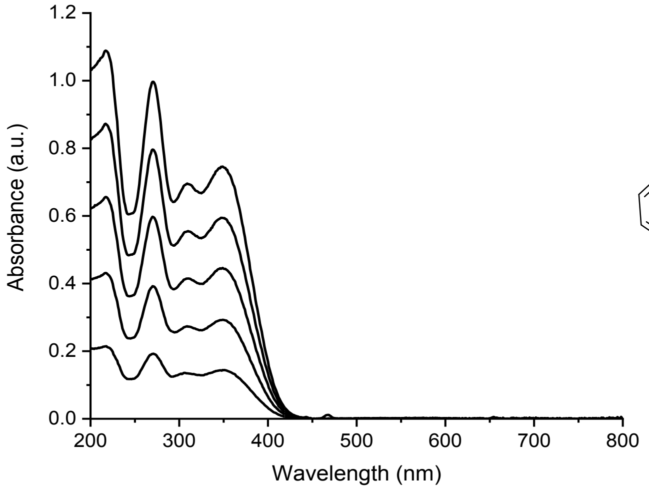

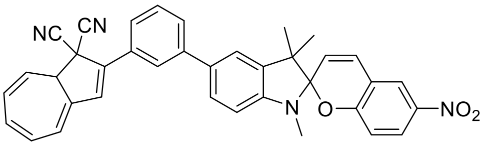

Fig. S64. Dilution row of **2**-*meta* in MeCN at 25 °C. Concentrations: $2.63\cdot10^{-5}$ M, $2.10\cdot10^{-5}$ M, $1.58\cdot10^{-5}$ M, $1.05\cdot10^{-5}$ M, and $5.26\cdot10^{-6}$ M.

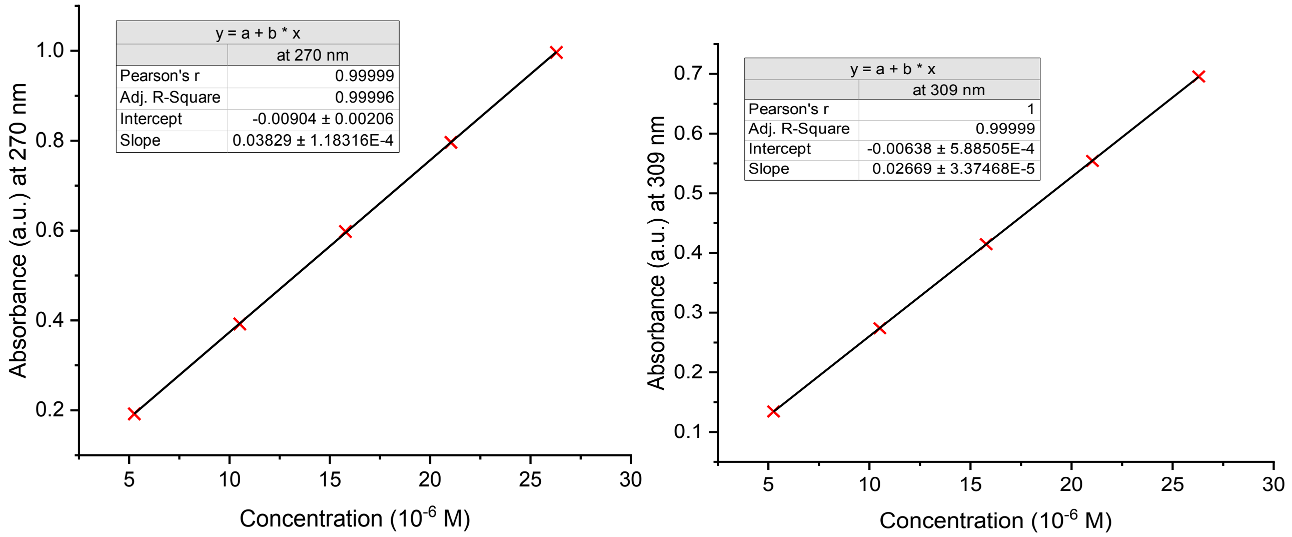

Fig. S65. Left: Absorption of **2**-*meta* at the maximum at 270 nm (red crosses) plotted vs. the concentration and fitting of the data to a linear function (black line). ε at 270 nm = $(38.3 \pm 0.1)\cdot10^3$ $M^{-1}cm^{-1}$. Right: Absorption of **2**-*meta* at the maximum at 309 nm (red crosses) plotted vs. the concentration and fitting of the data to a linear function (black line). ε at 309 nm = $(26.7 \pm 0.03)\cdot10^3$ $M^{-1}cm^{-1}$.

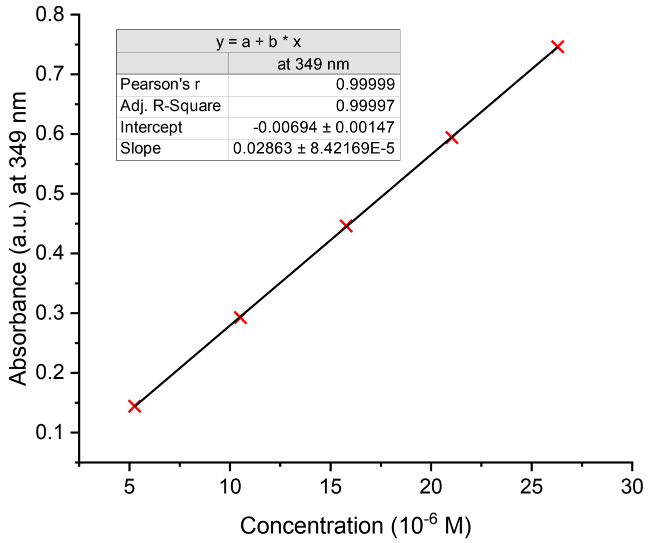

Fig. S66. Absorption of **2**-*meta* at the maximum at 349 nm (red crosses) plotted vs. the concentration and fitting of the data to a linear function (black line). ε at 349 nm = (28.6 ± 0.08)·10^3 $M^{-1}cm^{-1}$.

Compound *meta*-azulene **11**

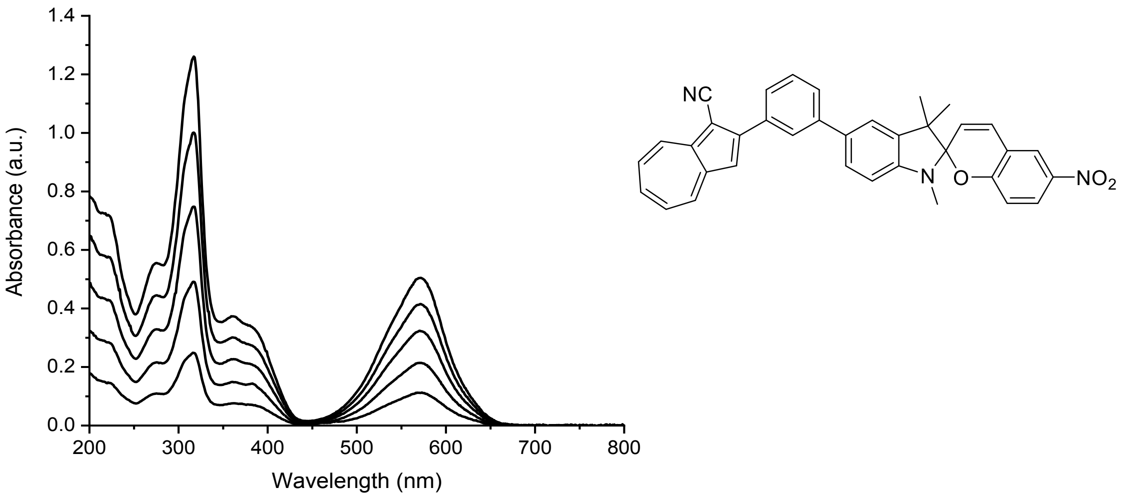

Fig. S67. Dilution row of *meta*-azulene **11** in MeCN at 25 °C. Concentrations: 1.77·10^{-5} M, 1.41·10^{-5} M, 1.06·10^{-5} M, 7.07·10^{-6} M, and 3.54·10^{-6} M.

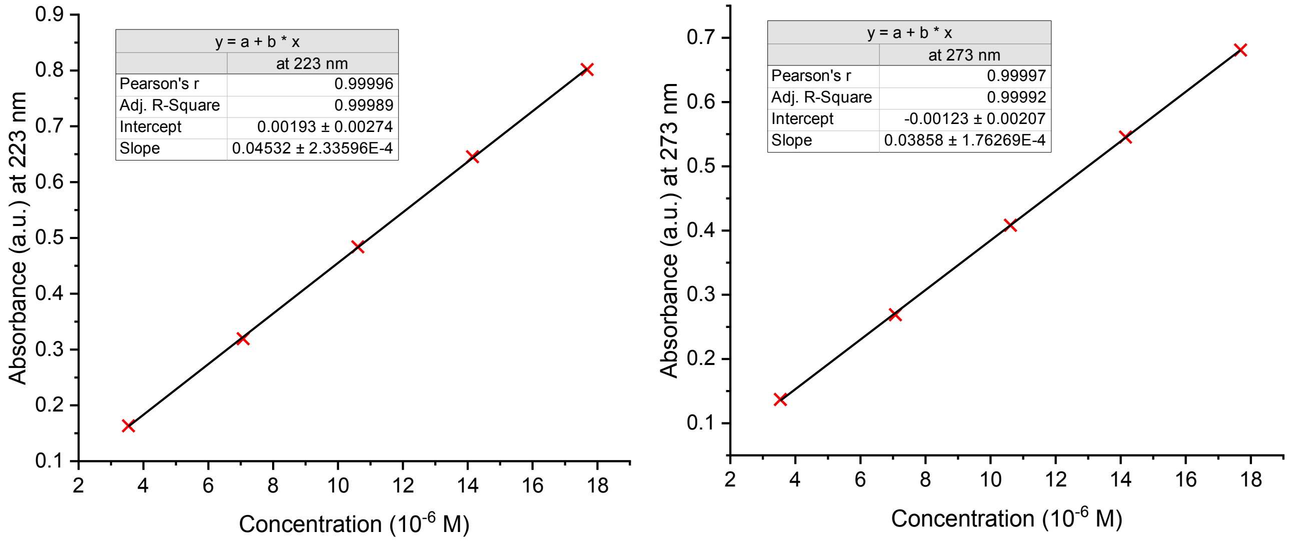

Fig. S68. Left: Absorption of *meta*-azulene **11** at the maximum at 223 nm (red crosses) plotted vs. the concentration and fitting of the data to a linear function (black line). ε at 223 nm = (45.3 ± 0.2)·10^3 $M^{-1}cm^{-1}$. Right: Absorption of *meta*-azulene **11** at the maximum at 273 nm (red crosses) plotted vs. the concentration and fitting of the data to a linear function (black line). ε at 273 nm = (38.6 ± 0.2)·10^3 $M^{-1}cm^{-1}$.

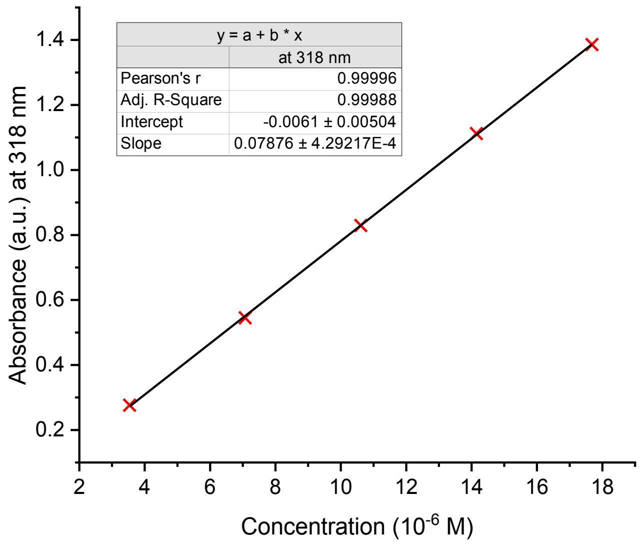

Fig. S69. Absorption of *meta*-azulene **11** at the maximum at 318 nm (red crosses) plotted vs. the concentration and fitting of the data to a linear function (black line). ε at 318 nm = (78.8 ± 0.4)·10^3 $M^{-1}cm^{-1}$.

Compound **2**-*para*

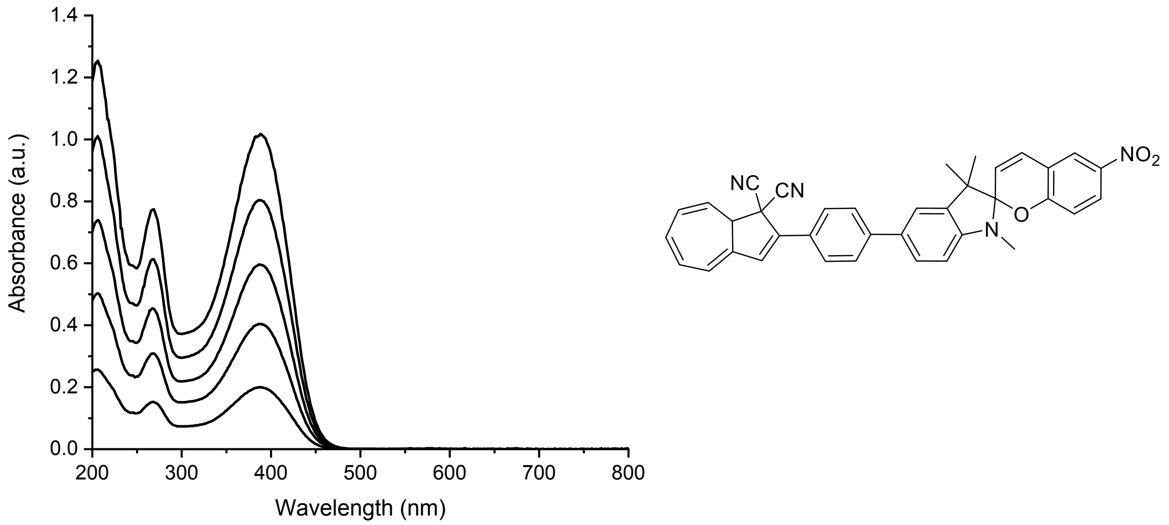

Fig. S70. Dilution row of **2**-*para* in MeCN at 25 °C. Concentrations: $2.50 \cdot 10^{-5}$ M, $2.00 \cdot 10^{-5}$ M, $1.50 \cdot 10^{-5}$ M, $9.99 \cdot 10^{-6}$ M, and $4.99 \cdot 10^{-6}$ M.

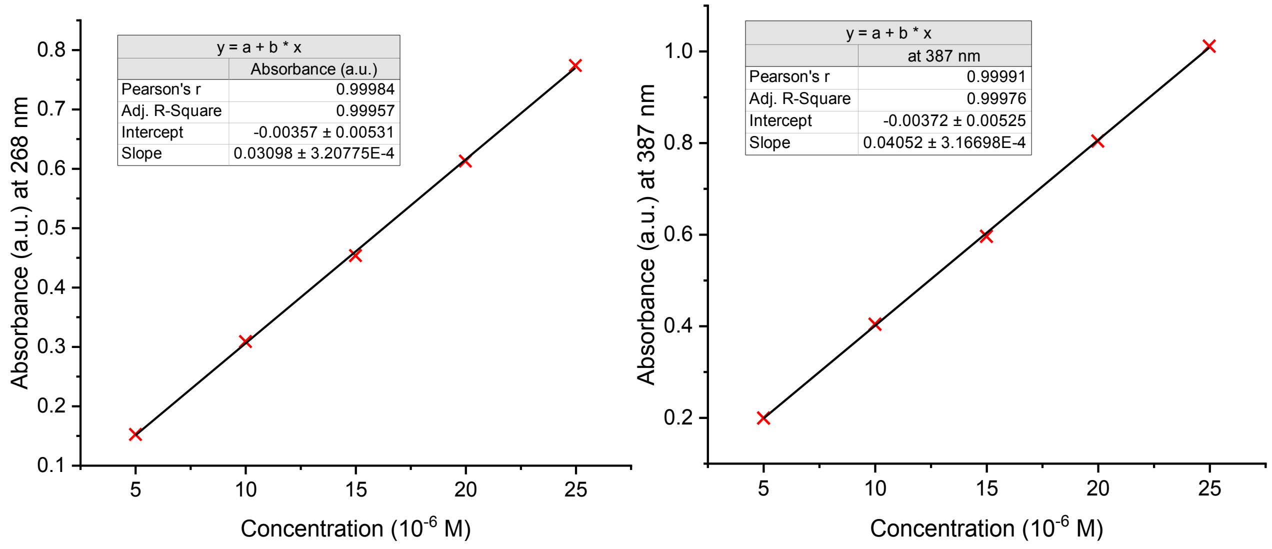

Fig. S71. Left: Absorption of **2**-*para* at the maximum at 268 nm (red crosses) plotted vs. the concentration and fitting of the data to a linear function (black line). ε at 268 nm = $(31.0 \pm 0.3) \cdot 10^3$ $M^{-1}cm^{-1}$. Right: Absorption of **2**-*para* at the maximum at 387 nm (red crosses) plotted vs. the concentration and fitting of the data to a linear function (black line). ε at 387 nm = $(40.5 \pm 0.3) \cdot 10^3$ $M^{-1}cm^{-1}$.

Compound *para*-azulene **10**

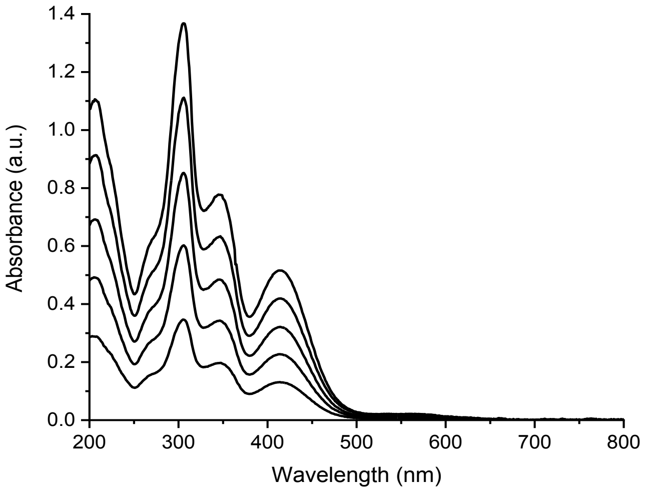

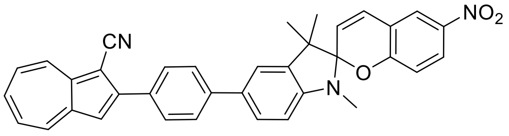

Fig. S72. Dilution row of *para*-azulene **10** in MeCN at 25 °C. Concentrations: $2.18\cdot10^{-5}$ M, $1.78\cdot10^{-5}$ M, $1.37\cdot10^{-5}$ M, $9.64\cdot10^{-6}$ M, and $5.58\cdot10^{-6}$ M .

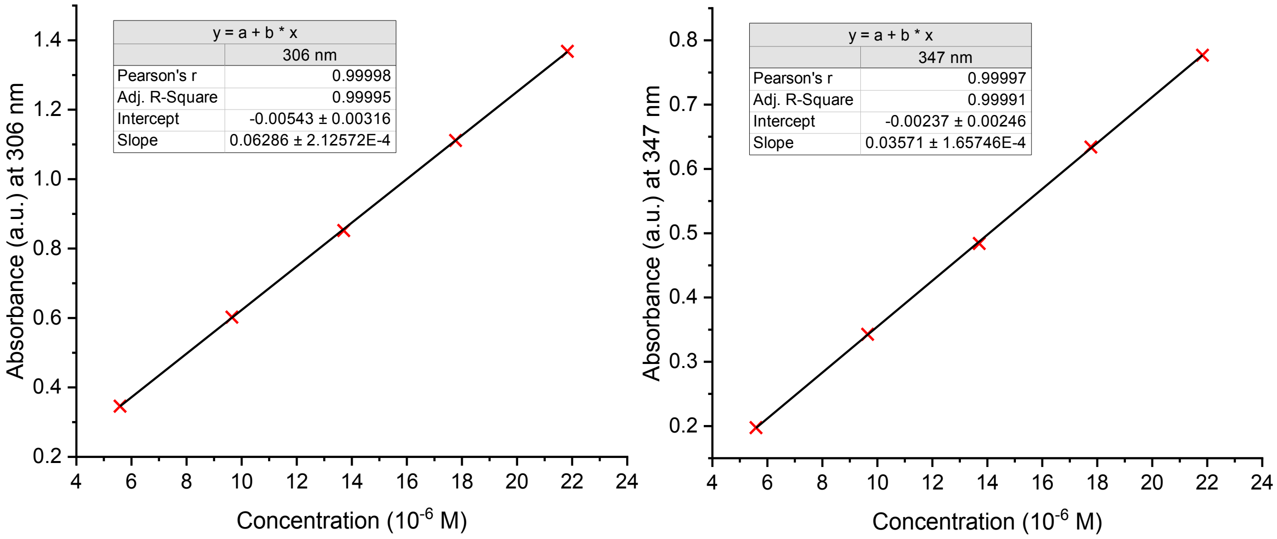

Fig. S73. Left: Absorption of *para*-azulene **10** at the maximum at 306 nm (red crosses) plotted vs. the concentration and fitting of the data to a linear function (black line). ε at 306 nm = $(62.9 \pm 0.2)\cdot10^{3}$ $M^{-1}cm^{-1}$. Right: Absorption of *para*-azulene **10** at the maximum at 347 nm (red crosses) plotted vs. the concentration and fitting of the data to a linear function (black line). ε at 347 nm = $(35.7 \pm 0.2)\cdot10^{3}$ $M^{-1}cm^{-1}$.

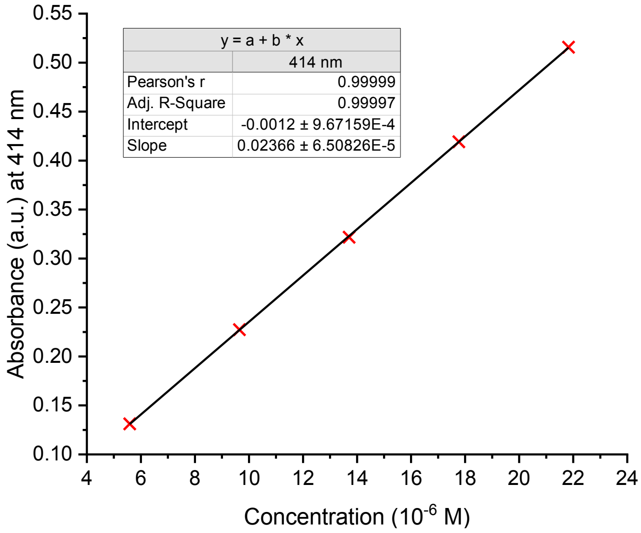

Fig. S74. Absorption of *para*-azulene **10** at the maximum at 414 nm (red crosses) plotted vs. the concentration and fitting of the data to a linear function (black line). ε at 414 nm = (23.6 ± 0.07)·10^3 $M^{-1}cm^{-1}$.

Thermal back-reaction analysis

Compound **1**-*meta*: VHF-MC form → VHF-SP form → DHA-SP form

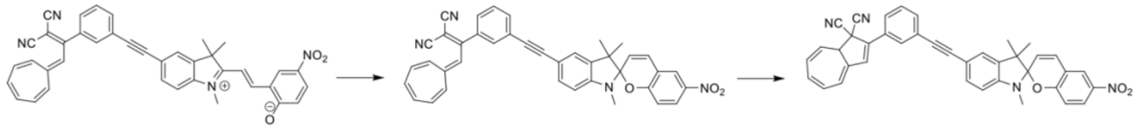

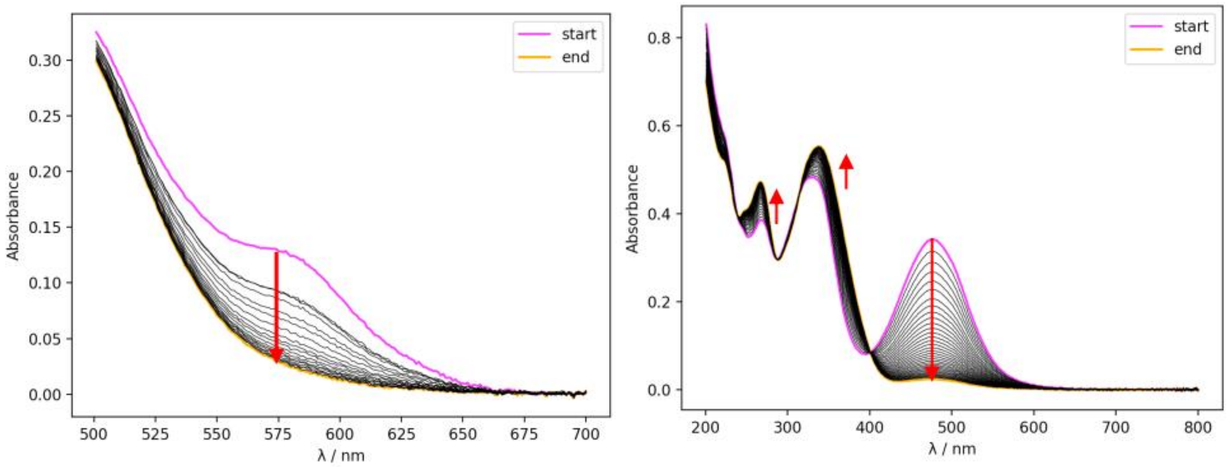

Fig. S75. Left: UV-Vis absorption spectra changes over 3 min, during the thermal back reaction from VHF-MC form to VHF-SP in MeCN at 25 °C. Right: UV-Vis absorption spectra changes during the thermal back reaction from VHF-SP form to DHA-SP in MeCN at 25 °C.

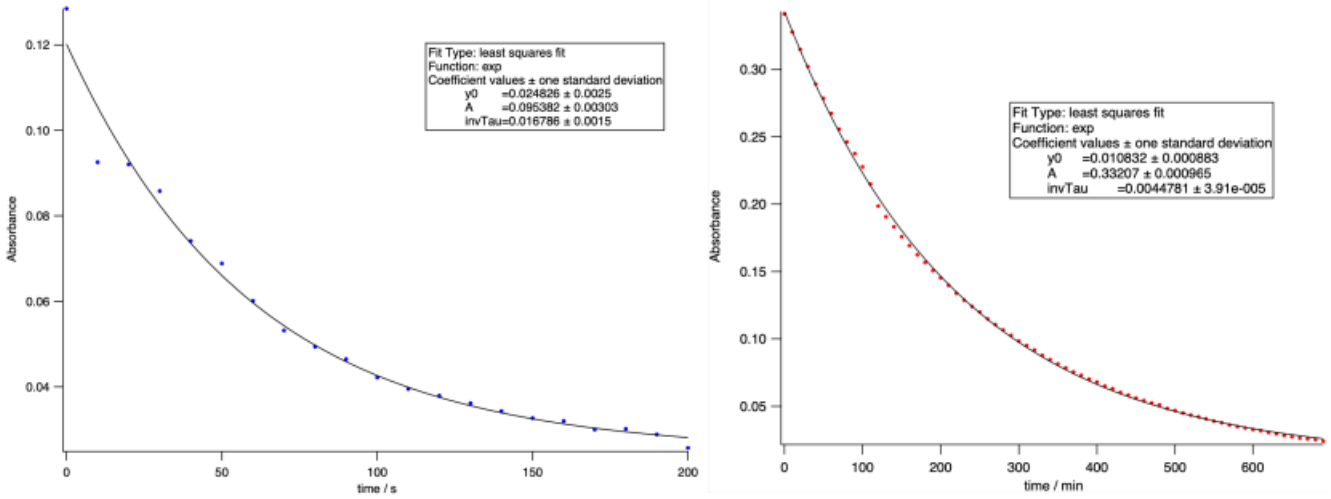

Fig. S76. Left: Decay of the absorbance at 575 nm for the thermal back reaction from VHF-MC form to VHF-SP form in MeCN at 25 °C (blue points) and fitting of the data to an exponential function (black line). InvTau here gives the rate constant. $k = 1.68{\cdot}10^{-2}$ s^{-1}, $t_{1/2} = 41$ sec, 0.68 min. Right: Decay of the absorbance at 478 nm for the thermal back reaction from VHF-SP form to DHA-SP form in MeCN at 25 °C (red points) and fitting of the data to an exponential function (black line). InvTau gives the rate constant in min^{-1}. $k = 7.46{\cdot}10^{-5}$ s^{-1}, $t_{1/2} = 155$ min.

Compound **1**-*ortho*: VHF-MC form → VHF-SP form → DHA-SP form

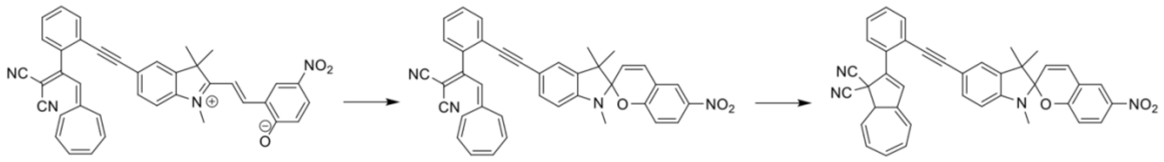

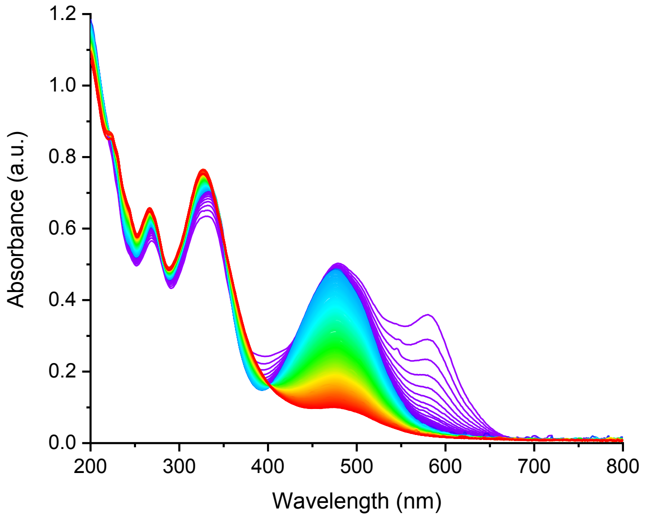

Fig. S77. UV-Vis absorption spectra changes during the thermal back reaction from VHF-MC form to VHF-SP form and then to DHA-SP form in MeCN at 25 °C.

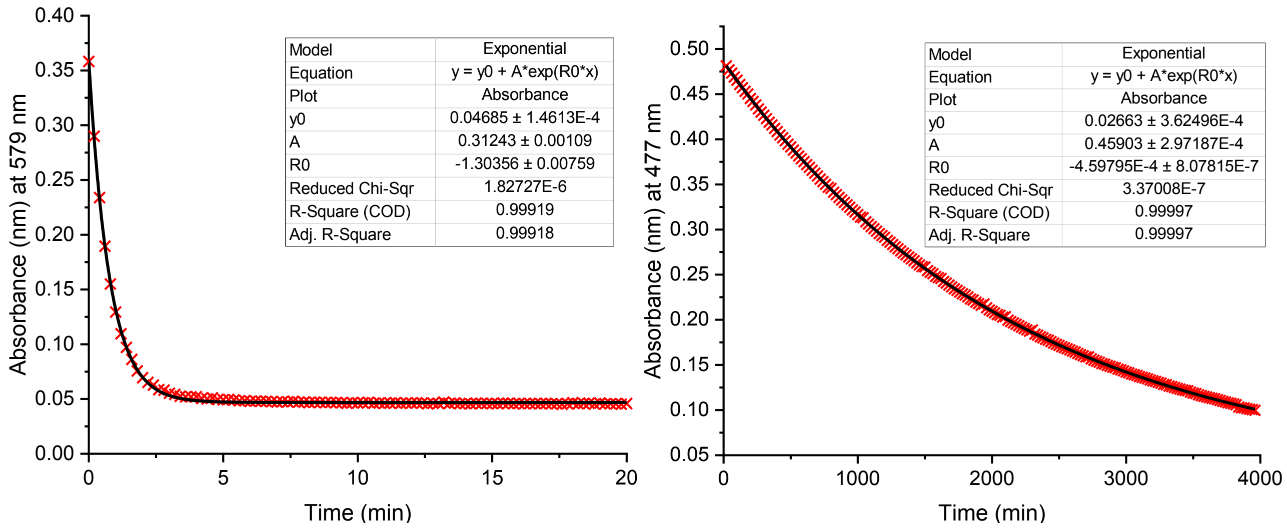

Fig. S78. Left: Decay of the absorbance at 579 nm for the thermal back reaction from VHF-MC form to VHF-SP form in MeCN at 25 °C (red crosses) and fitting of the data to an exponential function (black line). -R0 gives the rate constant in min^{-1}. $k = 2.17 \cdot 10^{-2}$ s^{-1}, $t_{1/2} = 0.53$ min. Right: Decay of the absorbance at 477 nm for the thermal back reaction from VHF-SP form to DHA-SP form in MeCN at 25 °C (red crosses, thermal back reaction not completed) and fitting of the data to an exponential function (black line). -R0 gives the rate constant in min^{-1}. $k = 7.66 \cdot 10^{-6}$ s^{-1}, $t_{1/2} = 1508$ min.

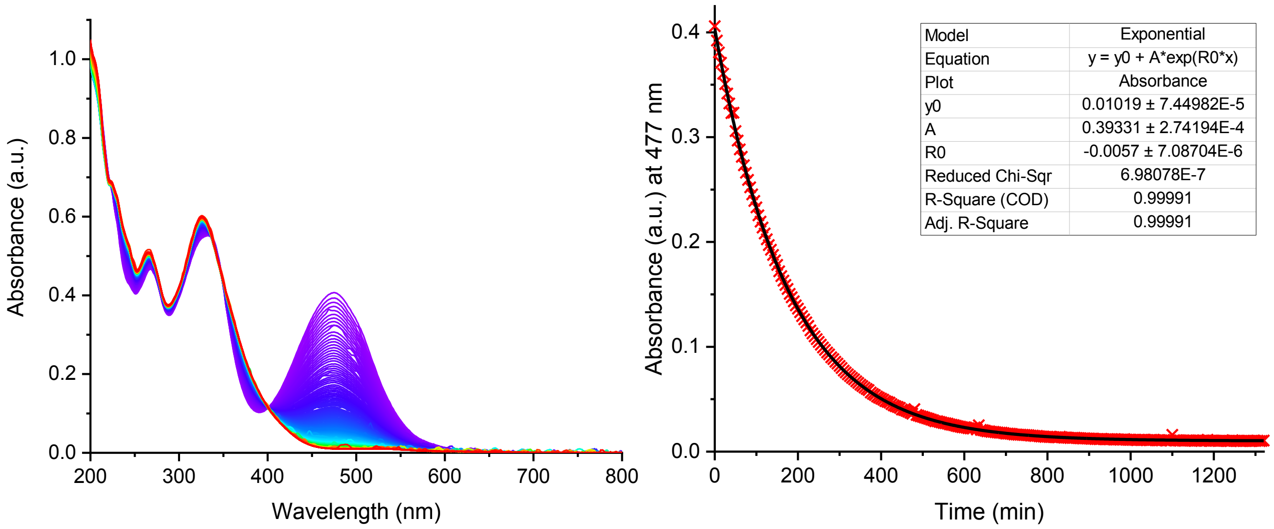

Fig. S79. Left: UV-Vis absorption spectra changes during the thermal back reaction from VHF-SP form to DHA-SP form in MeCN at 45 °C. Right: Decay of the absorbance at 477 nm for the thermal back reaction from VHF-SP form to DHA-SP form in MeCN at 45 °C (red crosses) and fitting of the data to an exponential function (black line). -R0 gives the rate constant in min^{-1}. $k = 9.49 \cdot 10^{-5}$ s^{-1}, $t_{1/2}$ = 122 min.

Compound **6**: MC form → SP form

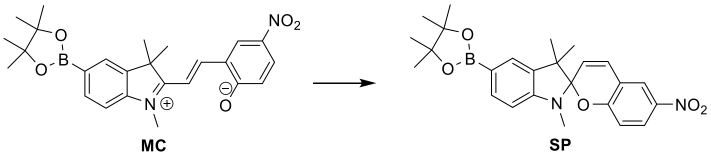

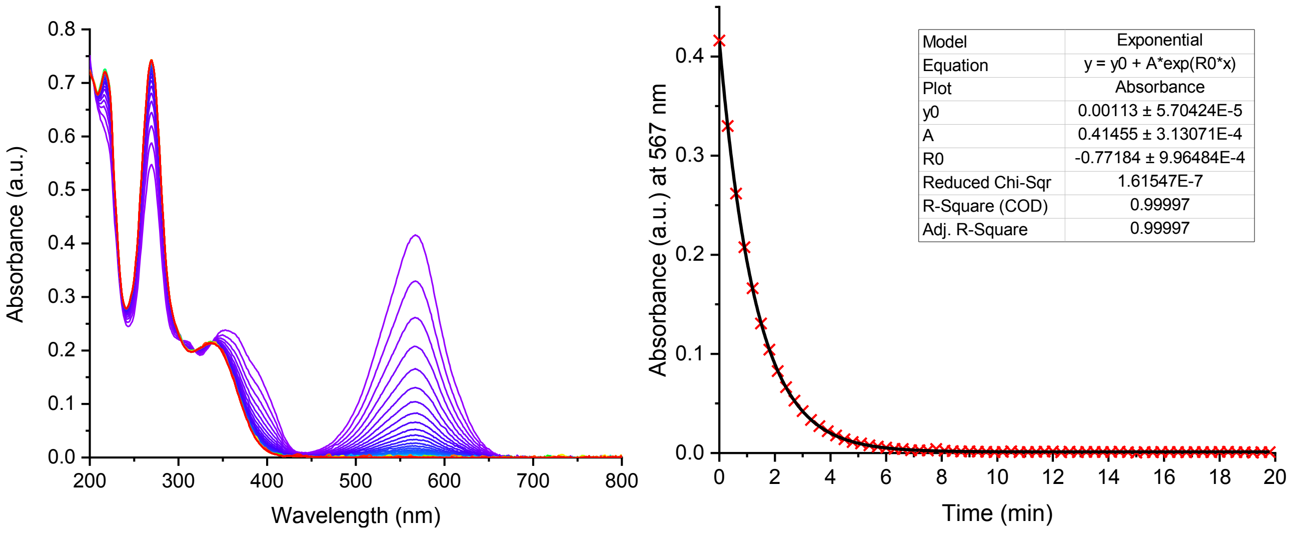

Fig. S80. Left: UV-Vis absorption spectra changes during the thermal back reaction from MC to SP in MeCN at 25 °C. Right: Decay of the absorbance at 567 nm for the thermal back reaction from MC to SP in MeCN at 25 °C (red crosses) and fitting of the data to an exponential function (black line). -R0 gives the rate constant in min^{-1}. $k = 1.29 \cdot 10^{-2}$ s^{-1}, $t_{1/2} = 0.90$ min.

Compound **2**-*meta* (365nm): VHF-MC form → VHF-SP form → DHA-SP form

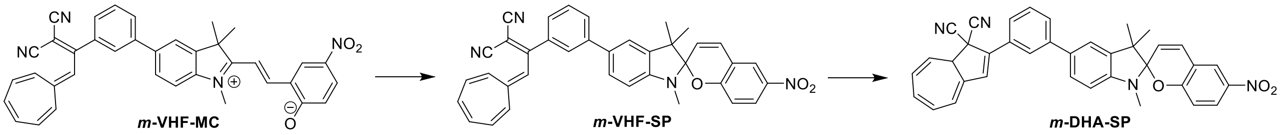

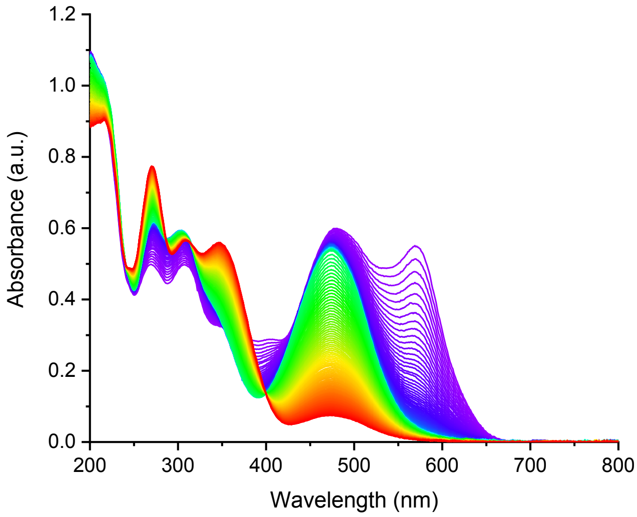

Fig. S81. UV-Vis absorption spectra changes during the thermal back reaction from VHF-MC to VHF-SP and then to DHA-SP in MeCN at 25 °C.

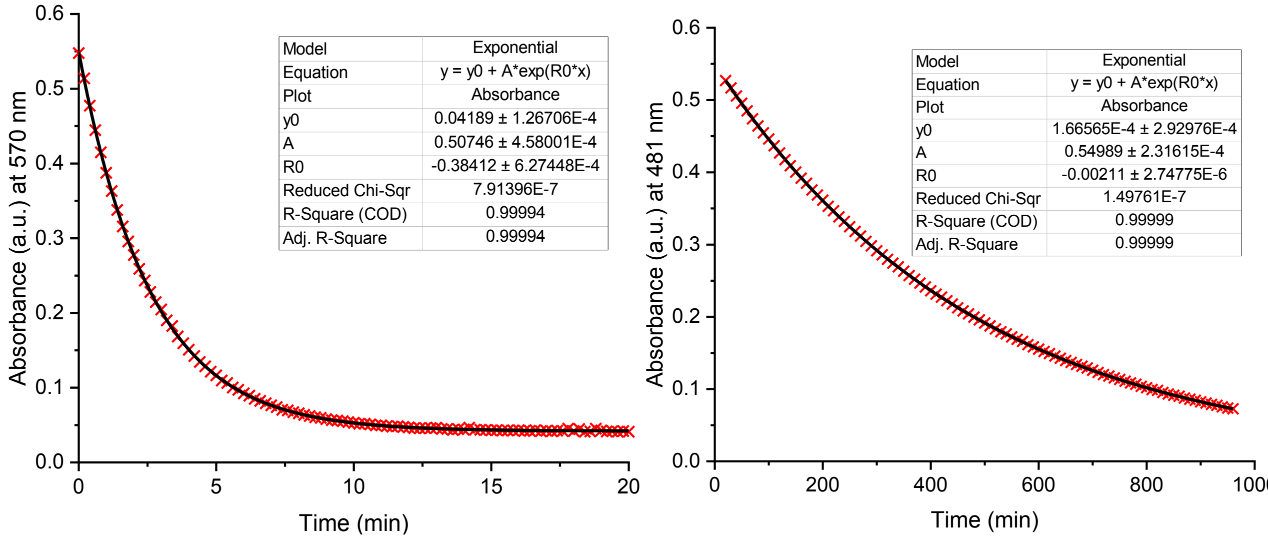

Fig. S82. Left: Decay of the absorbance at 570 nm for the thermal back reaction from VHF-MC to VHF-SP in MeCN at 25 °C (red crosses) and fitting of the data to an exponential function (black line). -R0 gives the rate constant in min^{-1}. $k = 6.40 \cdot 10^{-3}$ s^{-1}, $t_{1/2}$ = 1.80 min. Right: Decay of the absorbance at 481 nm for the thermal back reaction from VHF-SP to DHA-SP in MeCN at 25 °C (red crosses) and fitting of the data to an exponential function (black line). -R0 gives the rate constant in min^{-1}. $k = 3.52 \cdot 10^{-5}$ s^{-1}, $t_{1/2}$ = 328 min.

Compound **2**-*meta* (415nm+TFA): VHF-*cis*MCH form → DHA-*cis*MCH form

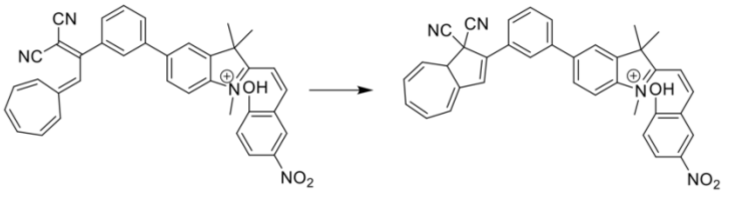

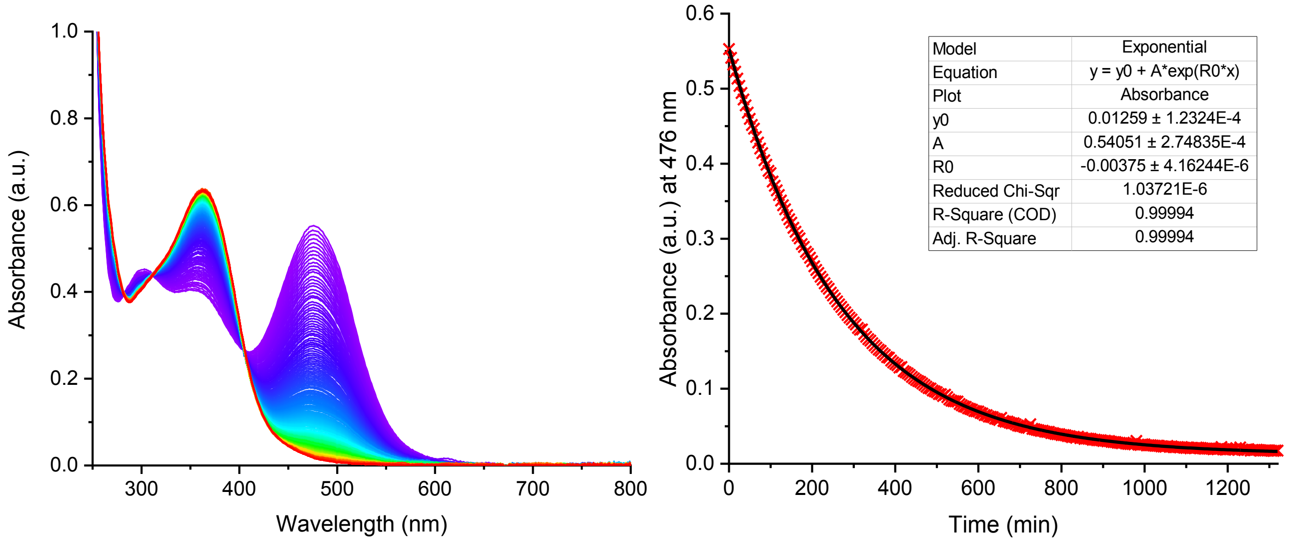

Fig. S83. Left: UV-Vis absorption spectra changes during the thermal back reaction from VHF-*cis*MCH to DHA-*cis*MCH in TFA/MeCN (1:29) at 25 °C. Right: Decay of the absorbance at 476 nm for the thermal back reaction from VHF-*cis*MCH to DHA-*cis*MCH in TFA/MeCN (1:29) at 25 °C (red crosses) and fitting of the data to an exponential function (black line). -R0 gives the rate constant in min^{-1}. $k = 6.25 \cdot 10^{-5}$ s^{-1}, $t_{1/2}$ = 185 min.

Compound **2**-*meta* (415nm+TFA+415nm): VHF-*trans*MCH form → DHA-*trans*MCH form

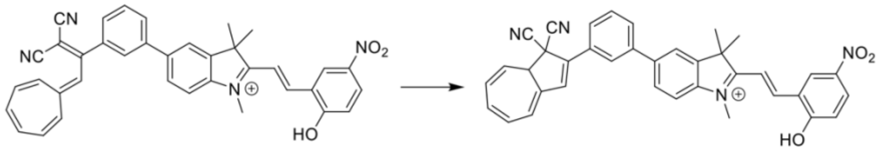

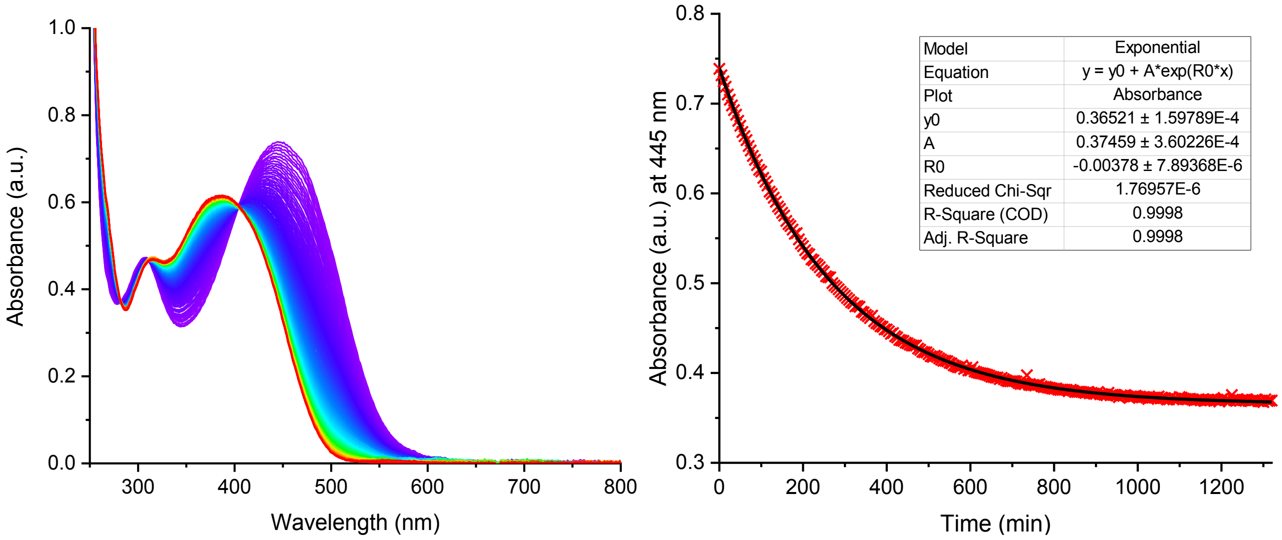

Fig. S84. Left: UV-Vis absorption spectra changes during the thermal back reaction from VHF-*trans*MCH to DHA-*trans*MCH in TFA/MeCN (1:29) at 25 °C. Right: Decay of the absorbance at 445 nm for the thermal back reaction from VHF-*trans*MCH to DHA-*trans*MCH in TFA/MeCN (1:29) at 25 °C (red crosses) and fitting of the data to an exponential function (black line). -R0 gives the rate constant in min^{-1}. $k = 6.30 \cdot 10^{-5}\ s^{-1}$, $t_{1/2}$ = 183 min.

Compound **2**-*meta*: DHA-MC form → DHA-SP form

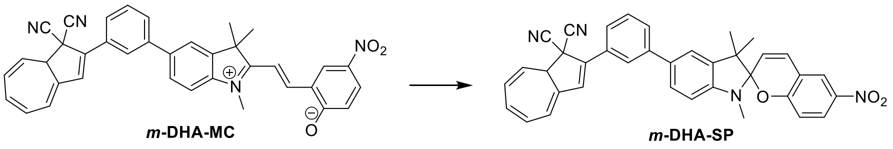

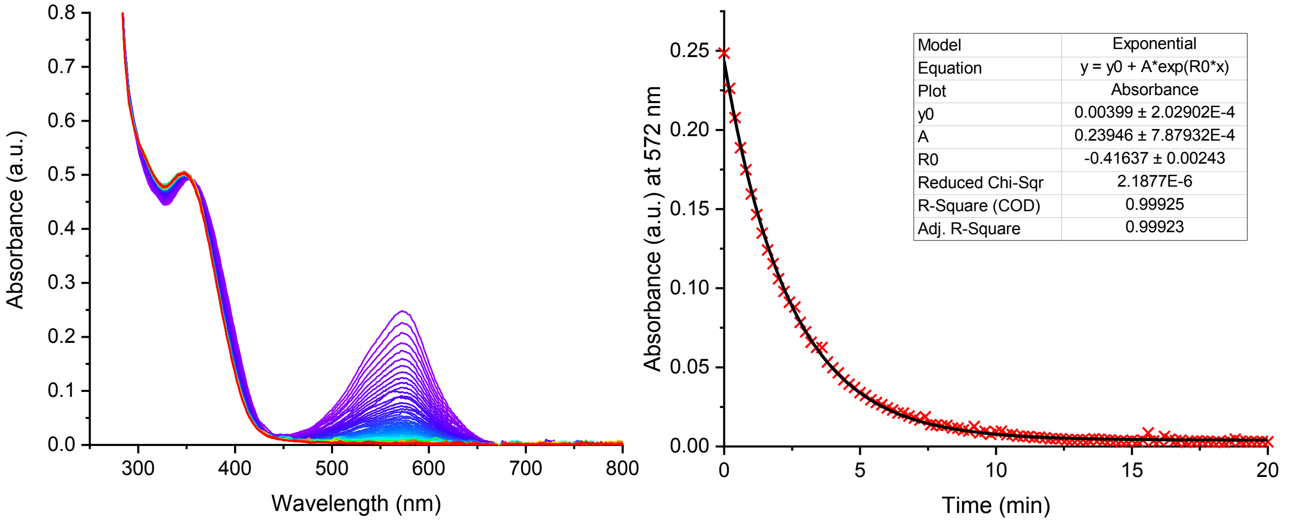

Fig. S85. Left: UV-Vis absorption spectra changes during the thermal back reaction from DHA-MC to DHA-SP in TFA/Et_3N/MeCN (1:6:29) Right: Decay of the absorbance at 572 nm for the thermal back reaction from DHA-MC to DHA-SP in TFA/Et_3N/MeCN (1:6:29) (red crosses) and fitting of the data to an exponential function (black line). -R0 gives the rate constant in min^{-1}. $k = 6.94 \cdot 10^{-3}$ s^{-1}, $t_{1/2}$ = 1.66 min.

Compound **2**-*meta* (TFA+415nm+NEt3): VHF-MC form → VHF-SP form

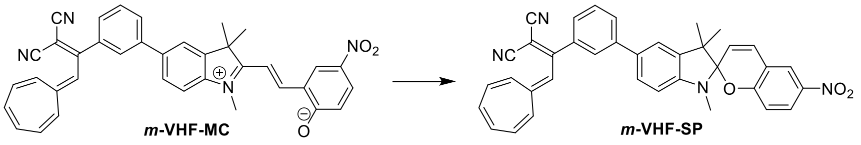

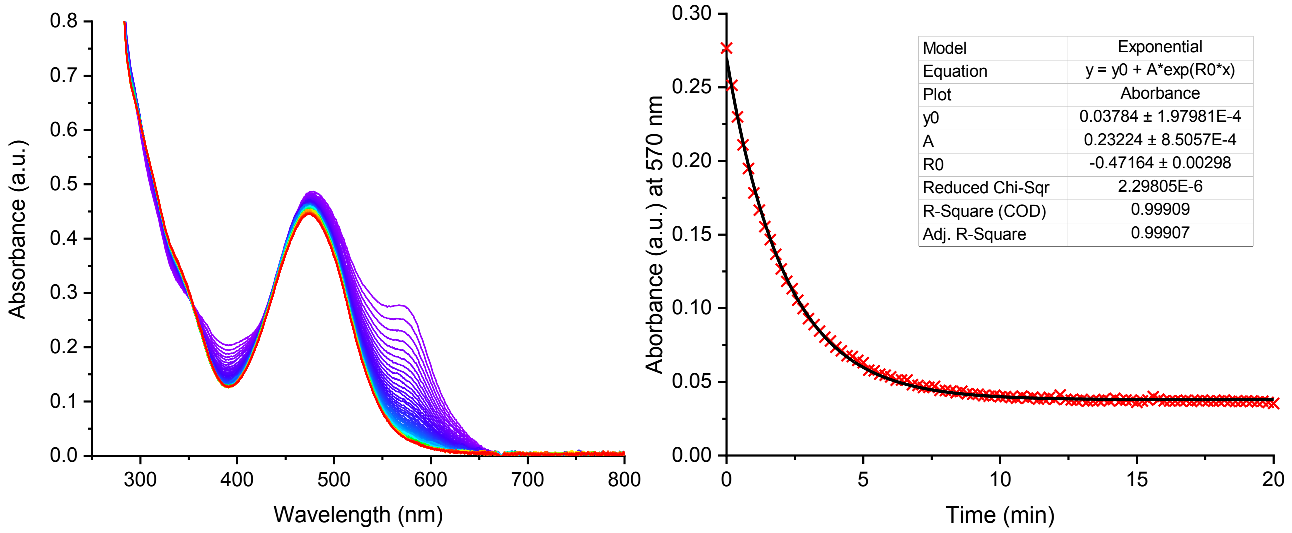

Fig. S86. Left: UV-Vis absorption spectra changes during the thermal back reaction from VHF-MC to VHF-SP in TFA/Et_3N/MeCN (1:6:29). Right: Decay of the absorbance at 570 nm for the thermal back reaction from VHF-MC to VHF-SP in TFA/Et_3N/MeCN (1:6:29) (red crosses) and fitting of the data to an exponential function (black line). -R0 gives the rate constant in min^{-1}. $k = 7.86 \cdot 10^{-3}$ s^{-1}, $t_{1/2}$ = 1.47 min.

Compound *meta*-azulene **11**: MC form → SP form

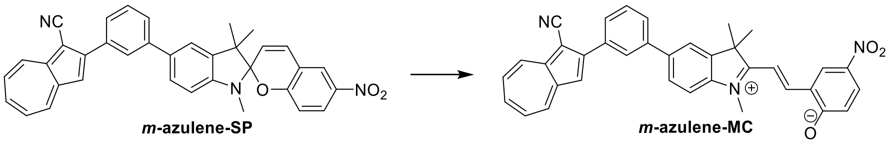

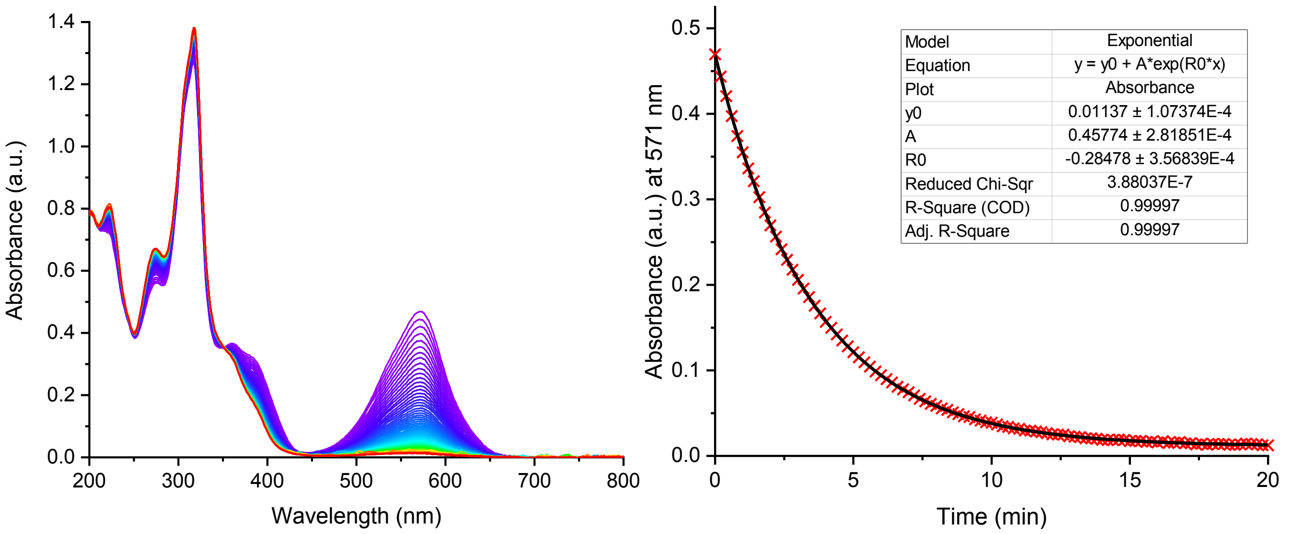

Fig. S87. Left: UV-Vis absorption spectra changes during the thermal back reaction from MC to SP form in MeCN at 25 °C, measured 5 times per min for 20 min. *m*-Azulene-MC was obtained by irradiation of *m*-azulene-SP at 365 nm (250 mA) for 10 min. Right: Decay of the absorbance at 571 nm for the thermal back reaction from MC form to SP form in MeCN at 25 °C (red crosses) and fitting of the data to an exponential function (black line). -R0 gives the rate constant in min^{-1}. $k = 4.75 \cdot 10^{-3}$ s^{-1}, $t_{1/2} = 2.43$ min.

Compound **2**-*para* (365nm): VHF-MC form → VHF-SP form → DHA-SP form

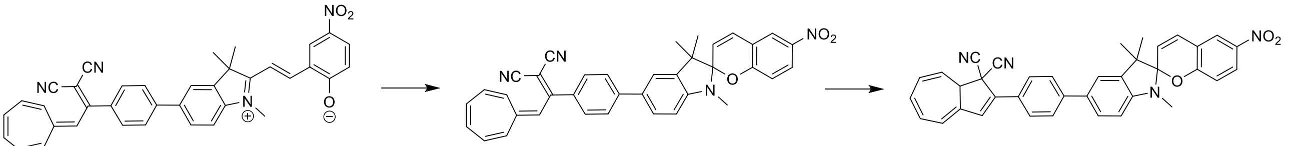

p-VHF-MC **p-VHF-SP** **p-DHA-SP**

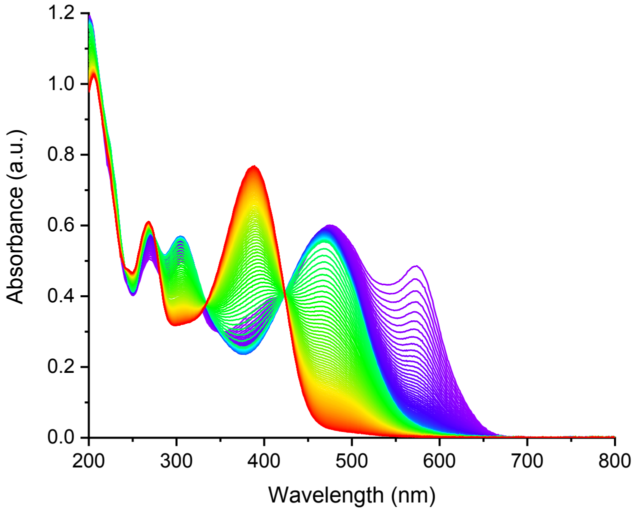

Fig. S88. UV-Vis absorption spectra changes during the thermal back reaction from VHF-MC to VHF-SP then to DHA-SP in MeCN at 25 °C.

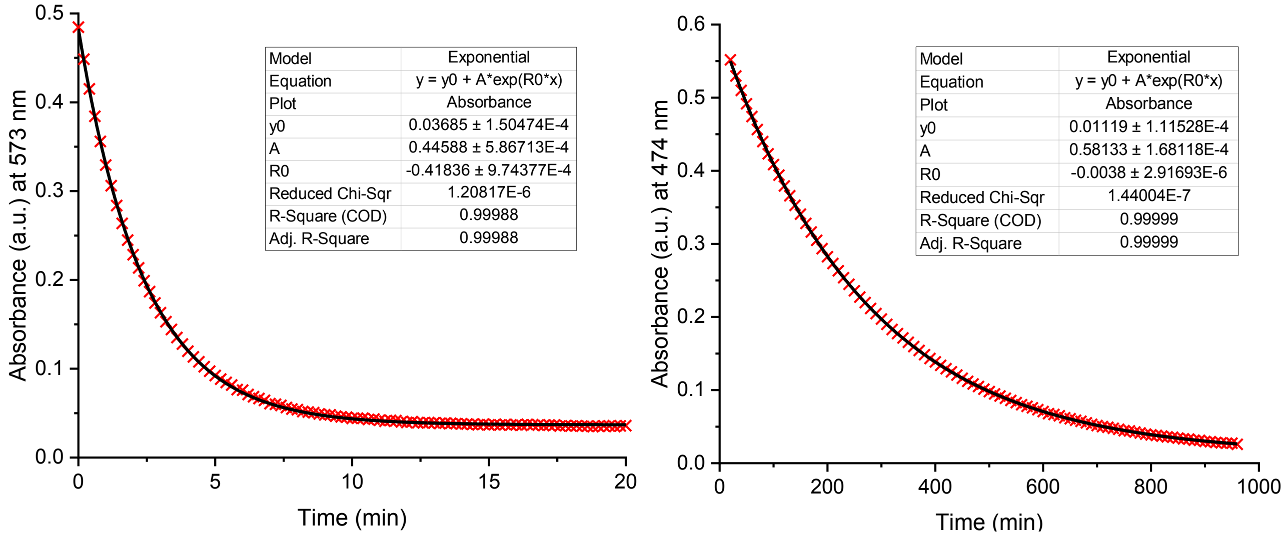

Fig. S89. Left: Decay of the absorbance at 573 nm for the thermal back reaction from VHF-MC to VHF-SP in MeCN at 25 °C (red crosses) and fitting of the data to an exponential function (black line). -R0 gives the rate constant in min^{-1}. $k = 6.97 \cdot 10^{-3}\ s^{-1}$, $t_{1/2} = 1.66$ min. Right: Decay of the absorbance at 474 nm for the thermal back reaction from VHF-SP to DHA-SP in MeCN at 25 °C (red crosses) and fitting of the data to an exponential function (black line). -R0 gives the rate constant in min^{-1}. $k = 6.33 \cdot 10^{-5}\ s^{-1}$, $t_{1/2} = 182$ min.

Compound **2**-*para* (415nm+TFA): VHF-*cis*MCH form → DHA-*cis*MCH form

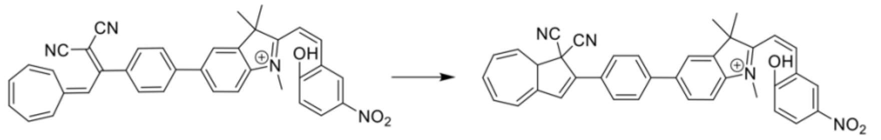

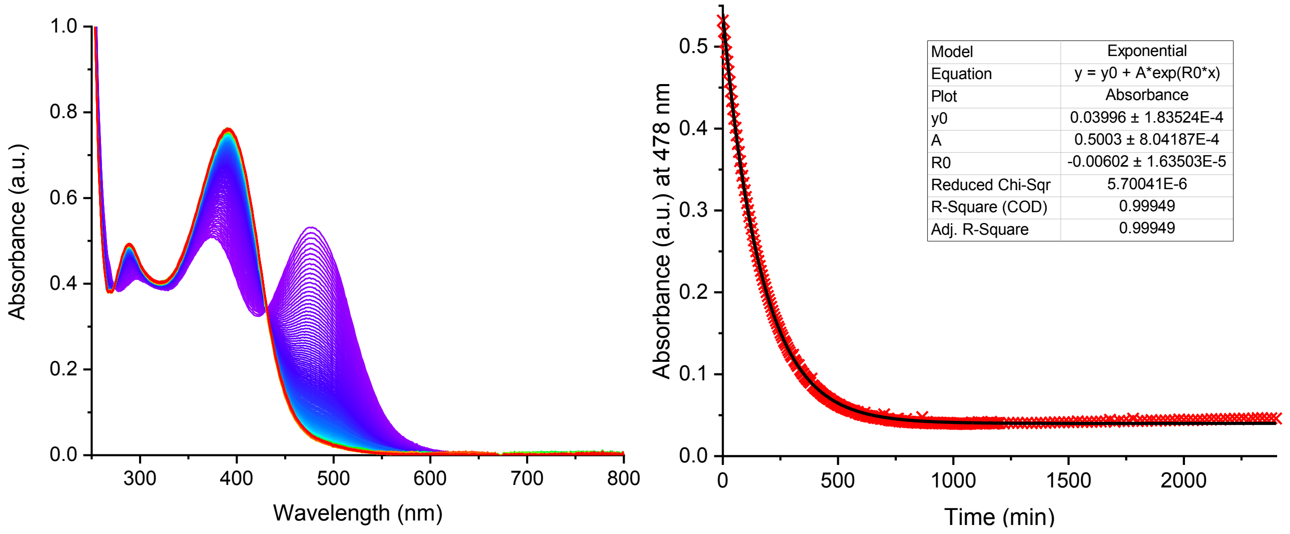

Fig. S90. Left: UV-Vis absorption spectra changes during the thermal back reaction from VHF-*cis*MCH to DHA-*cis*MCH in TFA/MeCN (1:29) at 25 °C. Right: Decay of the absorbance at 478 nm for the thermal back reaction from VHF-*cis*MCH to DHA-*cis*MCH in TFA/MeCN (1:29) at 25 °C (red crosses) and fitting of the data to an exponential function (black line). -R0 gives the rate constant in min^{-1}. $k = 1.00 \cdot 10^{-4}\ s^{-1}$, $t_{1/2}$ = 115 min.

Compound **2**-*para* (415nm+TFA+415nm): VHF-*trans*MCH form → DHA-*trans*MCH form

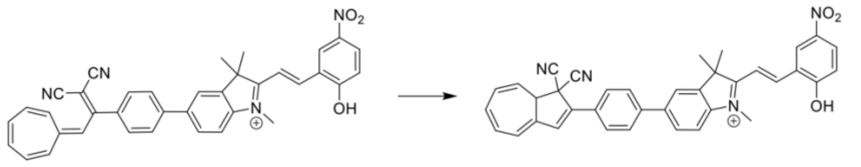

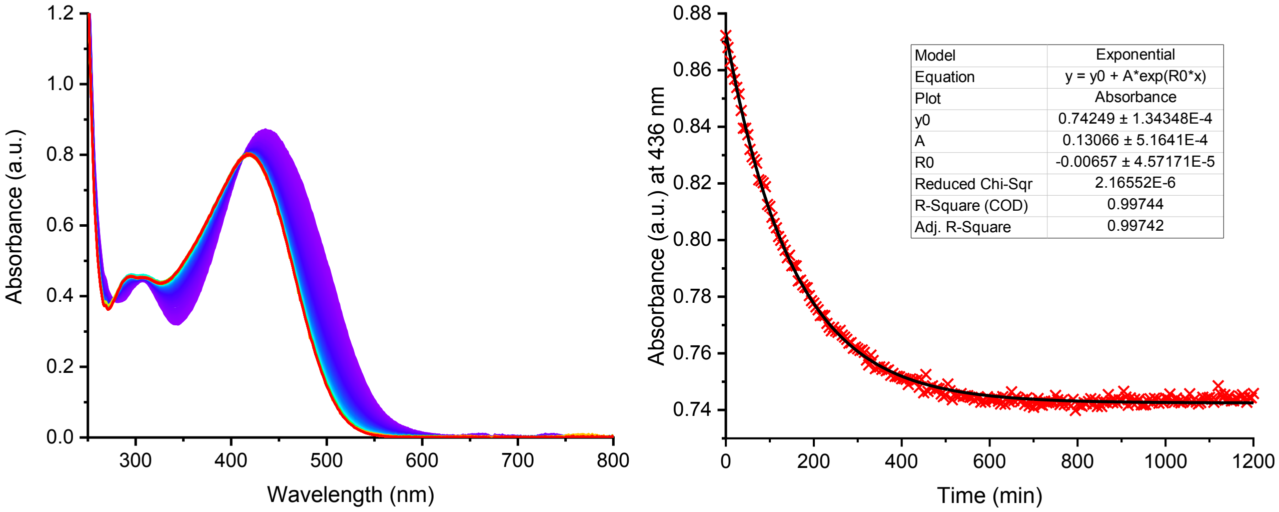

Fig. S91. Left: UV-Vis absorption spectra changes during the thermal back reaction from VHF-*trans*MCH to DHA-*trans*MCH in TFA/MeCN (1:29) at 25 °C. Right: Decay of the absorbance at 436 nm for the thermal back reaction from VHF-*trans*MCH to DHA-*trans*MCH in TFA/MeCN (1:29) at 25 °C (red crosses) and fitting of the data to an exponential function (black line). -R0 gives the rate constant in min^{-1}. $k = 1.09 \cdot 10^{-4}$ s^{-1}, $t_{1/2}$ = 106 min.

Compound **2**-*para* (TFA+415nm+NEt_3): DHA-MC form → DHA-SP form

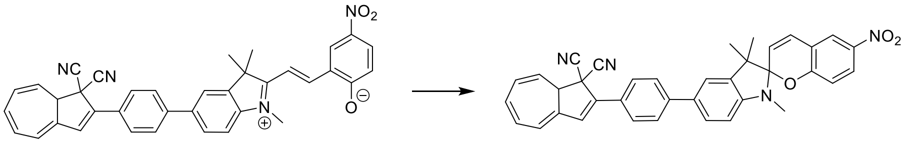

***p*-DHA-MC** ***p*-DHA-SP**

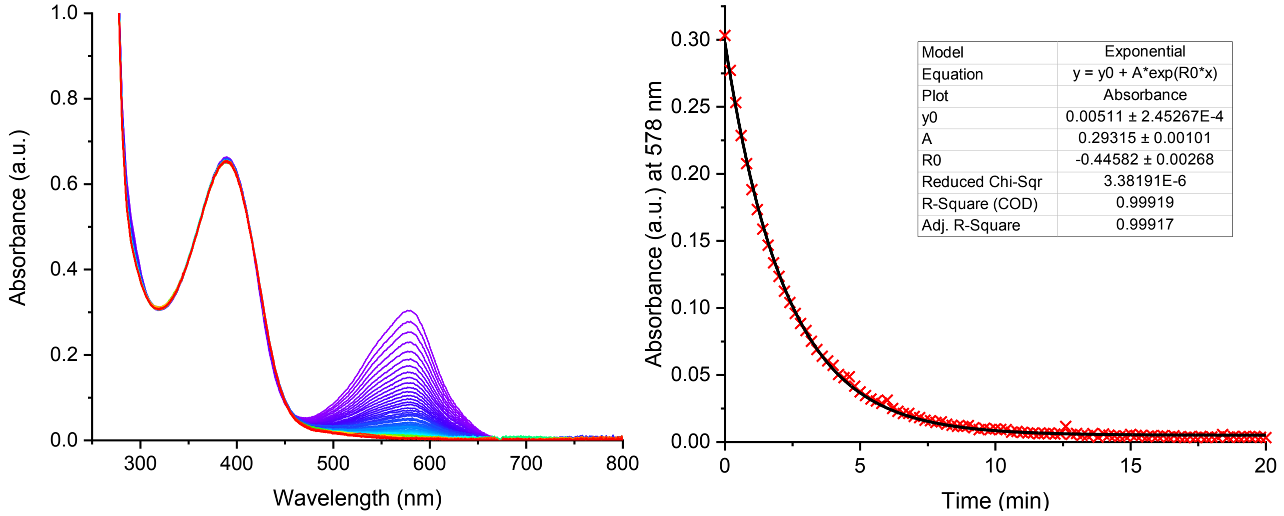

Fig. S92. Left: UV-Vis absorption spectra changes during the thermal back reaction from DHA-MC to DHA-SP in TFA/Et_3N/MeCN (1:6:29). Right: Decay of the absorbance at 578 nm for the thermal back reaction from DHA-MC to DHA-SP in TFA/Et_3N/MeCN (1:6:29) (red crosses) and fitting of the data to an exponential function (black line). -R0 gives the rate constant in min^{-1}. $k = 7.43 \cdot 10^{-3}$ s^{-1}, $t_{1/2}$ = 1.55 min.

Compound **2**-*para* (415nm+TFA+415nm+NEt_3): VHF-MC form → VHF-SP form

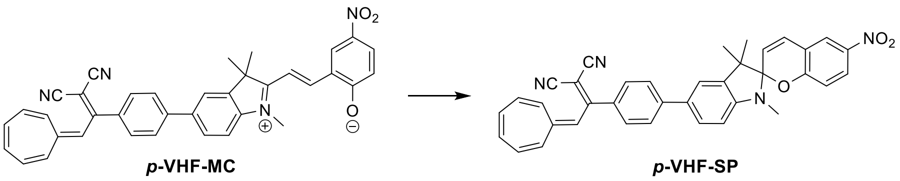

***p*-VHF-MC** ***p*-VHF-SP**

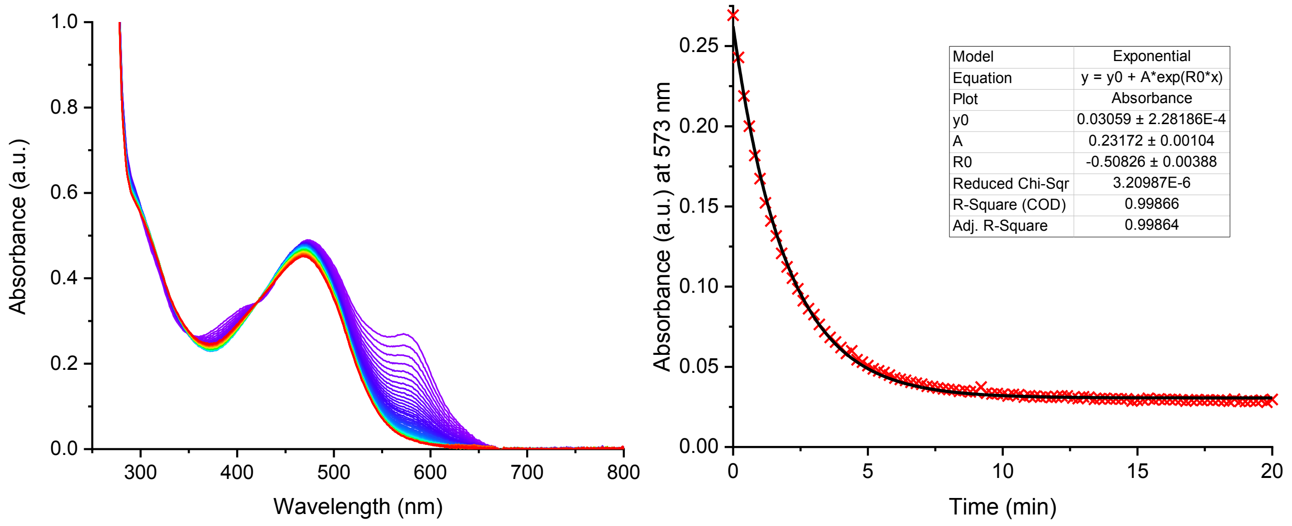

Fig. S93. Left: UV-Vis absorption spectra changes during the thermal back reaction from VHF-MC to VHF-SP in TFA/Et_3N/MeCN (1:6:29). Right: Decay of the absorbance at 573 nm for the thermal back reaction from VHF-MC to VHF-SP in TFA/Et_3N/MeCN (1:6:29) (red crosses) and fitting of the data to an exponential function (black line). -R0 gives the rate constant in min^{-1}. $k = 8.47 \cdot 10^{-3}$ s^{-1}, $t_{1/2}$ = 1.36 min.

Compound *para*-azulene **10**: MC form → SP form

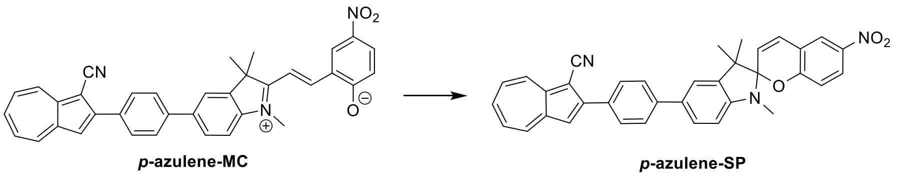

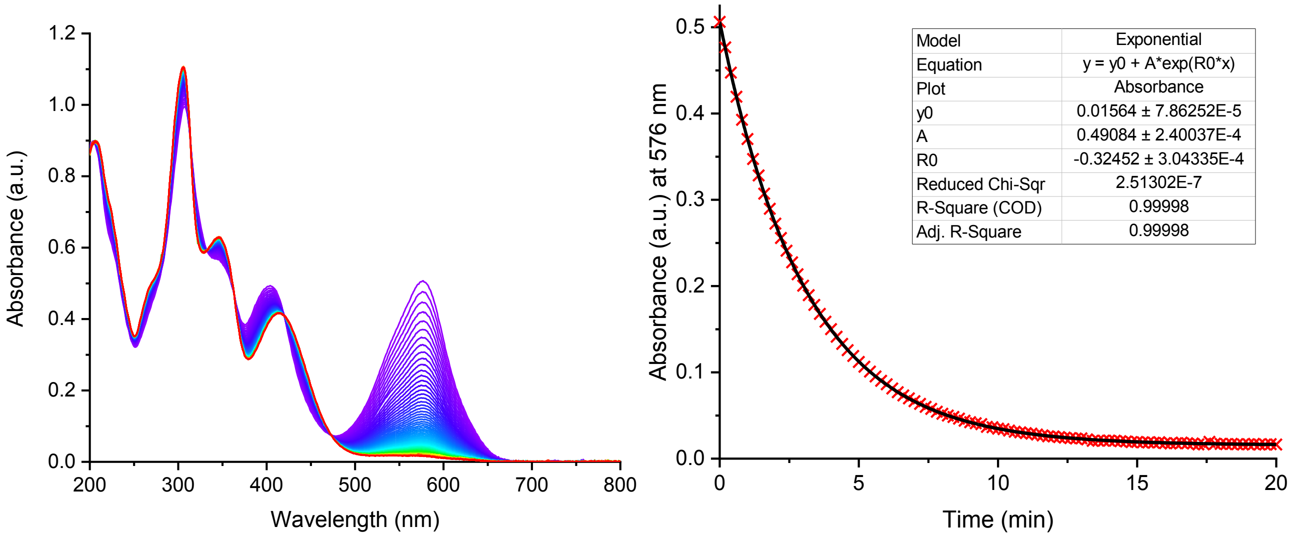

Fig. S94. Left: UV-Vis absorption spectra changes during the thermal back reaction from MC to SP in MeCN at 25 °C. *p*-Azulene-MC was obtained by irradiation of *p*-azulene-SP at 365 nm (250 mA) for 10 min. Right: Decay of the absorbance at 576 nm for the thermal back reaction from MC to SP in MeCN at 25 °C (red crosses) and fitting of the data to an exponential function (black line). -R0 gives the rate constant in min^{-1}. $k = 5.41 \cdot 10^{-3}$ s^{-1}, $t_{1/2} = 2.14$ min.

Influence of the status of one photoswitch on the thermal back reaction of the other

Table S2. Half-life (min) of MC to SP, in MeCN at 25 °C unless otherwise stated.

Substituent R	R-MC --> R-SP
Boronic ester **6**	0.90
Azulene **10** (*para*)	2.14
Azulene **11** (*meta*)	2.43
DHA (**2**-*para*)	1.55
DHA (**2**-*meta*)	1.66
VHF (**2**-*para*)	1.66
VHF (**2**-*meta*)	1.80
VHF (**2**-*para*)	1.36 (3% TFA in MeCN + NEt_3)
VHF (**2**-*meta*)	1.47 (3% TFA in MeCN + NEt_3)

Table S3. Half-life (min) of VHF to DHA, in MeCN at 25 °C unless otherwise stated.

Substituent R	R-VHF → R-DHA
H (reference system)	210[16]
SP (**2**-*para*)	182
SP (**2**-*meta*)	328
*cis*MCH (**2**-*para*)	115 (3% TFA in MeCN)
*cis*MCH (**2**-*meta*)	185 (3% TFA in MeCN)
*trans*MCH (**2**-*para*)	106 (3% TFA in MeCN)
*trans*MCH (**2**-*meta*)	183 (3% TFA in MeCN)

[16] S.L. Broman, S.L. Brand, C.R. Parker, M.Å Petersen, C.G. Tortzen, A. Kadziola, K. Kilså, M. B. Nielsen, *Arkivoc* **2011**, *ix*, 51–67.

Fluorescence Spectra

Emission spectra were measured on a Fluotime 300 (PicoQuant GmBH).

Excitation spectra were measured on a Cary Eclipse (Agilent).

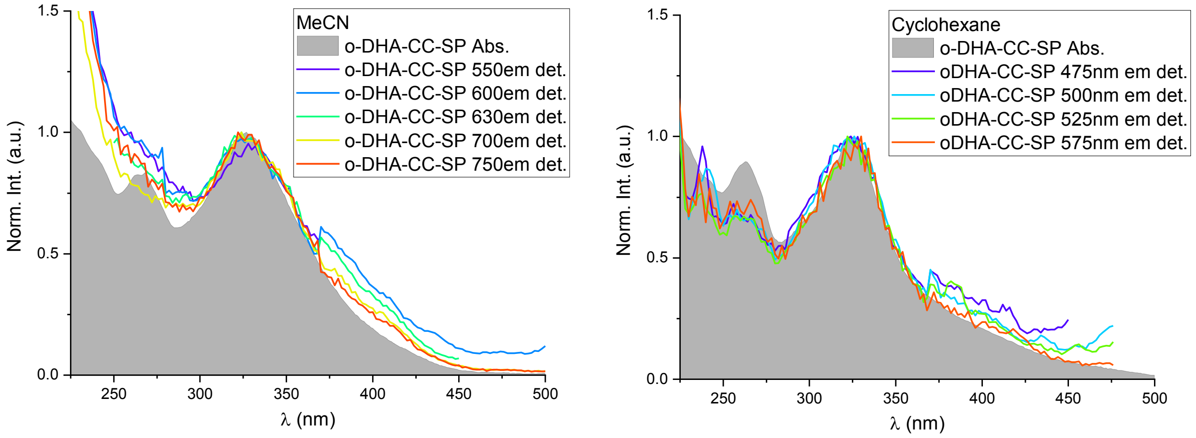

Fig. S95. Excitation and absorption spectra of o-DHA-CC-SP (**1**-*ortho*) in MeCN (left) and cyclohexane (right)

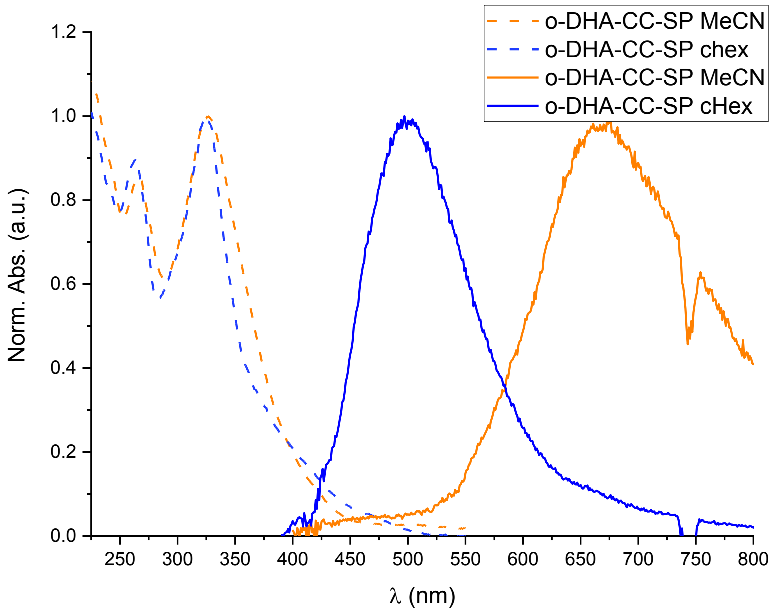

Fig. S96. Absorption (dashed lines) and emission (full lines) spectra of o-DHA-CC-SP (**1**-*ortho*) in MeCN (orange) and cyclohexane (blue).

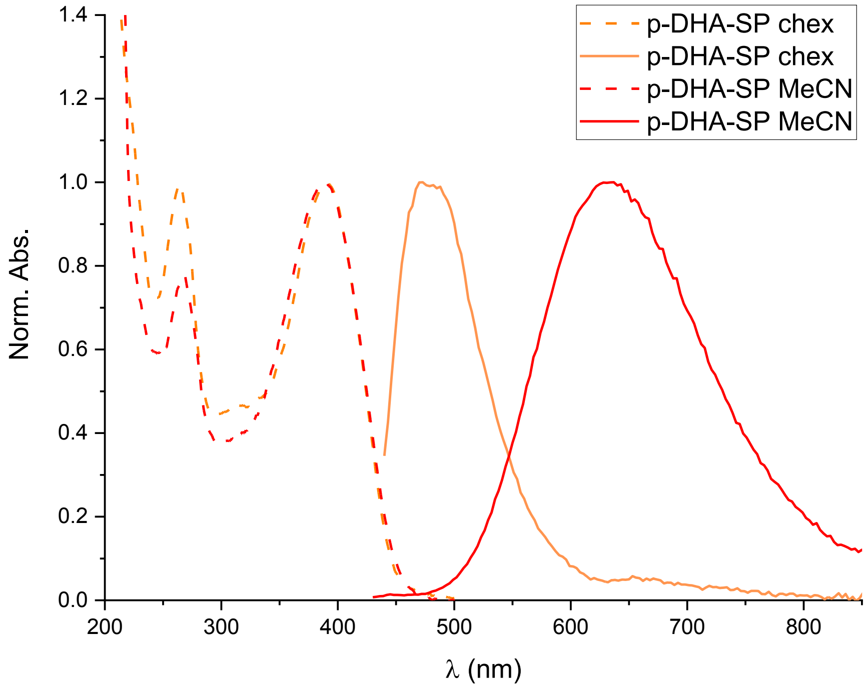

Fig. S97. Absorption (dashed lines) and emission (full lines) spectra of p-DHA-SP (**2**-*para*) in MeCN (red) and cyclohexane (orange).

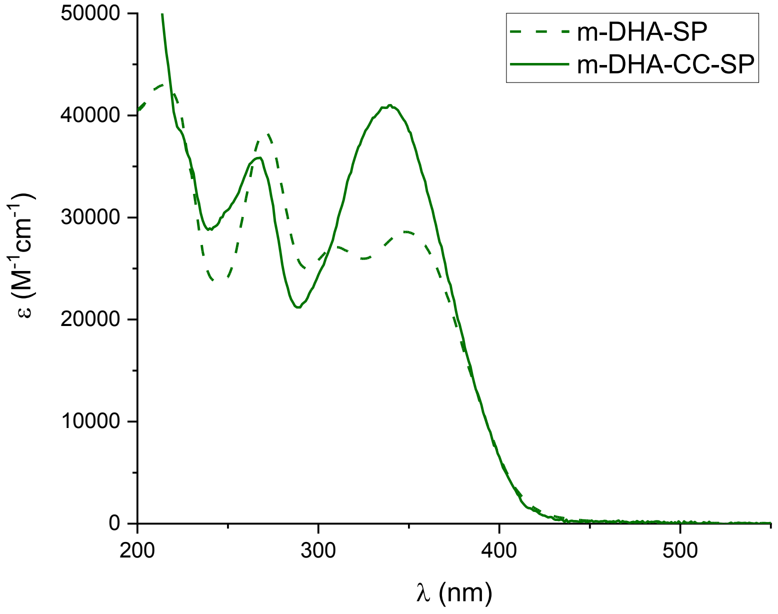

Fig. S98. Absorption coefficients of m-DHA-SP (**2**-*meta*) and m-DHA-CC-SP (**1**-*meta*) in MeCN.

Quantum yield determination.

Due to the photoswitching nature of the derivatives, multiple measurements at different concentrations were not possible, and the following results also contain some uncertainty.

The quantum yields (I_x) were therefore calculated relative to **1**-*para* in MeCN previously determined to 0.37 % (Φ_{ref}).[3]

$$\Phi = \Phi_{ref} \cdot \frac{I_x}{I_{ref}}$$

Where the intensities were corrected by the absorption at the excitation wavelength ($1\text{-}10^{-A}$) and integrated.

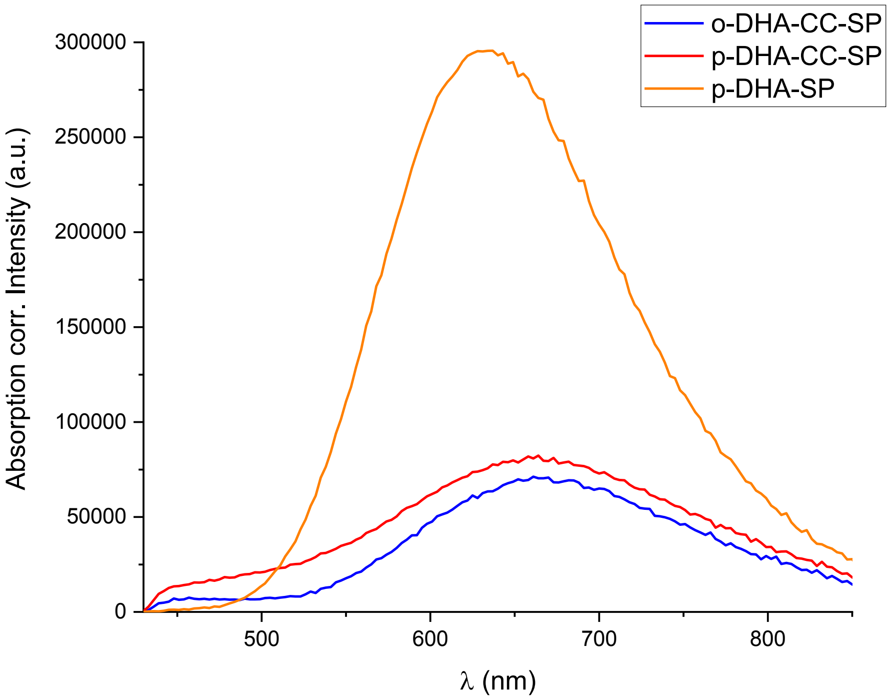

Fig. S99. Absorption corrected emission intensities of o-DHA-CC-SP (**1**-*ortho*, blue), p-DHA-CC-SP (red, **1**-*para*) and p-DHA-SP (orange, **2**-*para*) in MeCN.

Quantum Yields: **1**-*ortho* 0.28 % and **2**-*para* 0.99 % (relative to **1**-*para* 0.37%).

[3] M. Dowds, S. G. Stenspil, J. H. de Souza, B. W. Laursen, M. Cacciarini, M. B. Nielsen, *ChemPhotoChem* **2022**, *6*, e202200152

Ultrafast spectroscopic analysis of DHA switching

<u>**2**-*para* in MeCN and Cyclohexane</u>

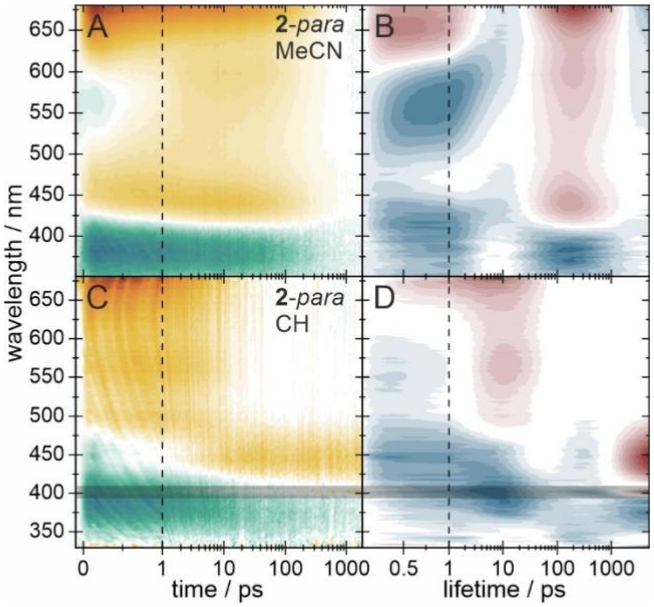

Fig. S100. Transient absorption spectra of **2**-*para* in MeCN (A) and cyclohexane (C) along the corresponding lifetime density maps (B, D) after excitation with 400 nm. Positive signals in A and C refer to absorption of excited states (ESA) or photoproducts (PA) whereas negative signals account for stimulated emission (SE) or ground state bleaching (GSB). In B and D positive components describe the rise of negative or decay of positive signals and negative components indicate decay of negative or rise of positive signals.

<u>**1**-*para* in DCM</u>

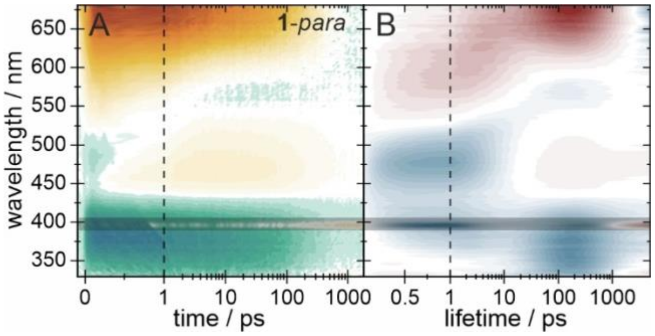

Fig. S101. Transient absorption spectrum of **1**-*para* in DCM (A) along the corresponding lifetime density map (B) after excitation with 400 nm. Positive signals in A refer to absorption of excited states (ESA) or photoproducts (PA) whereas negative signals account for stimulated emission (SE) or ground state bleaching (GSB). In B positive components describe the rise of negative or decay of positive signals and negative components indicate decay of negative or rise of positive signals.

2-*meta* in DCM, Photostationary State at 405 nm

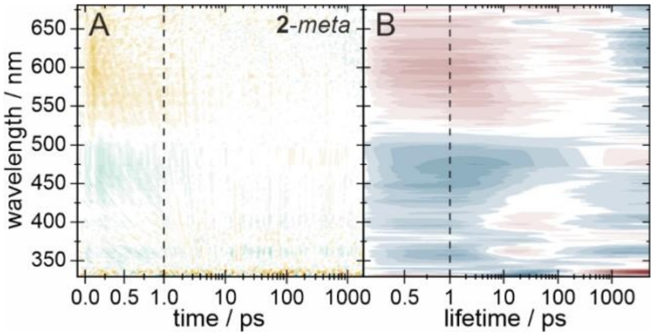

Fig. S102. Transient absorption spectrum of **2**-*meta* in DCM (A) along the corresponding lifetime density map (B) after excitation from the 405 nm photostationary state with 490 nm. Positive signals in A refer to absorption of excited states (ESA) or photoproducts (PA) whereas negative signals account for stimulated emission (SE) or ground state bleaching (GSB). In B positive components describe the rise of negative or decay of positive signals and negative components indicate decay of negative or rise of positive signals.

DHA-Ph in DCM

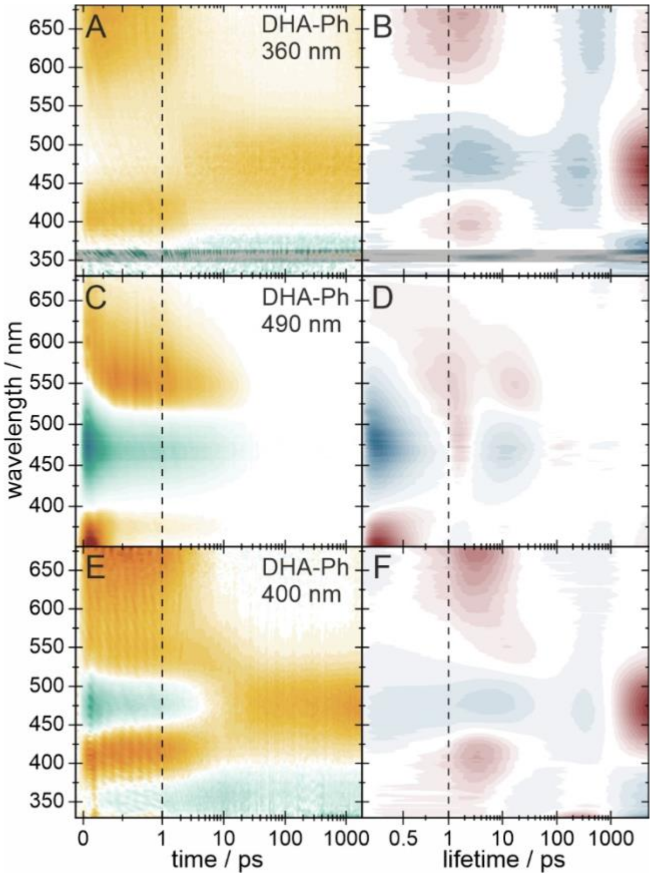

Fig. S103. Transient absorption spectra of DHA-Ph in DCM, excited at 360 nm (A), 400 nm (E) and from the 405 nm photostationary state with 490 nm (C) along the corresponding lifetime

density maps (B,D,F). Positive signals in A, C and E refer to absorption of excited states (ESA) or photoproducts (PA) whereas negative signals account for stimulated emission (SE) or ground state bleaching (GSB). In B, D and F positive components describe the rise of negative or decay of positive signals and negative components indicate decay of negative or rise of positive signals.

Ultrafast spectroscopic analysis of SP switching

2-*para*, **1**-*ortho*, **2**-*meta* and SP in DCM on the ns timescale

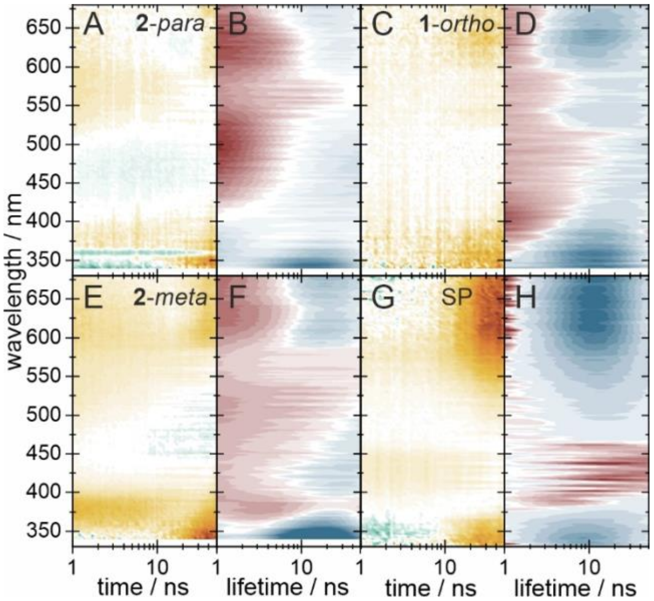

Fig. S104. ns Transient absorption spectra of **2**-*para* (A), **1**-*ortho* (C), **2**-*meta* (E) and SP monomer (G) along the corresponding lifetime density maps (B, D, F, H) in DCM after excitation from the 405 nm photostationary state with 360 nm. Positive signals in A, C, E and G refer to absorption of excited states (ESA) or photoproducts (PA) whereas negative signals account for stimulated emission (SE) or ground state bleaching (GSB). In B, D, F and H positive components describe the rise of negative or decay of positive signals and negative components indicate decay of negative or rise of positive signals.

SP in DCM

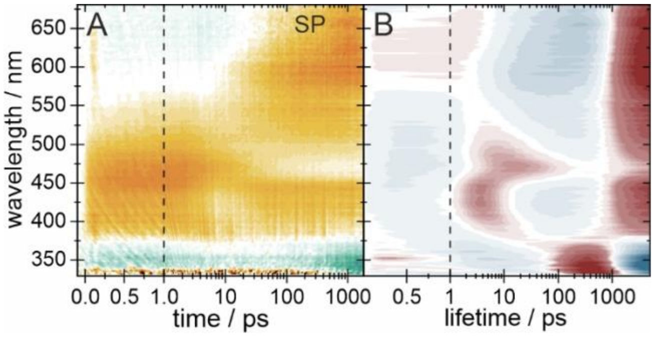

Fig. S105. Transient absorption spectrum of SP monomer in DCM (A) along the corresponding lifetime density map (B) after excitation from the 405 nm photostationary state with 360 nm. Positive signals in A refer to absorption of excited states (ESA) or photoproducts (PA) whereas negative signals account for stimulated emission (SE) or ground state bleaching (GSB). In B positive components describe the rise of negative or decay of positive signals and negative components indicate decay of negative or rise of positive signals.

Comparison of Steady State with Transient Spectrum after 100 ns

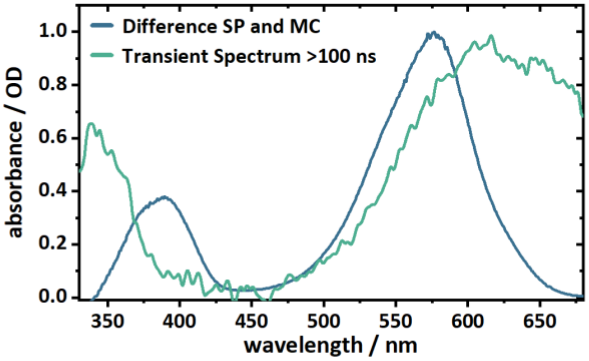

Fig. S106. Normalized steady state absorption of the difference between NO_2-SP and NO_2-MC in blue and normalized transient spectrum averaged over several timepoints after 100 ns.

Steady State Switching in CH_2Cl_2

<u>**2**-*para* and **2**-*meta* CH_2Cl_2</u>

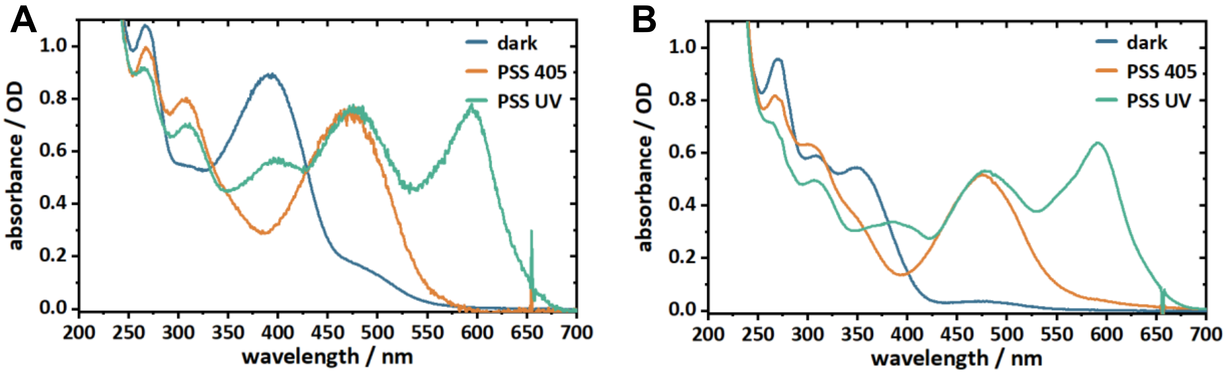

Fig. S.107: Photoswitching of **2**-*para* (A) and **2**-*meta* (B) from their dark DHA-SP state (blue line) with 405 nm LED light to a DHA-VHF dominated photostationary state (orange line). Subsequent or immediate UV illumination (<370 nm) leads to a photostationary state consisting mostly of VHF-MC (green line).

Final Overview

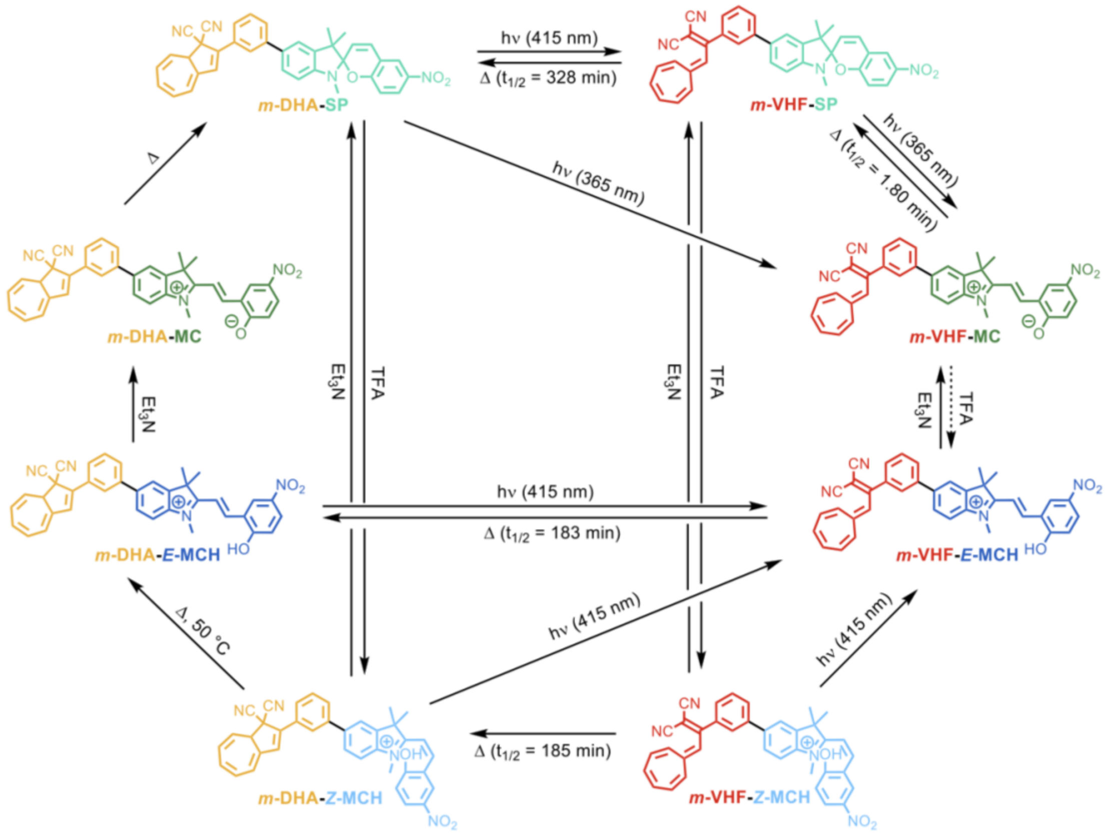

Fig. S108. General Overview of the switching states for **2**-*meta*

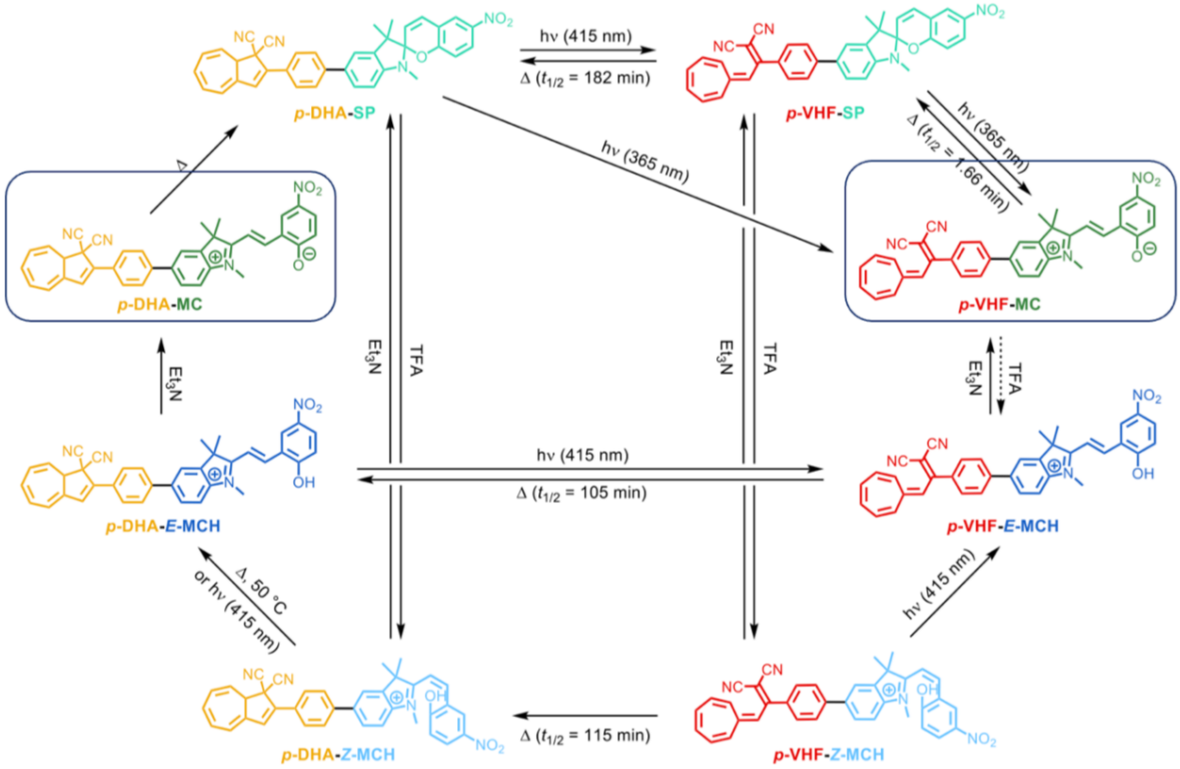

Fig. S109. General Overview of the switching states for **2**-*para*

Crystal Data

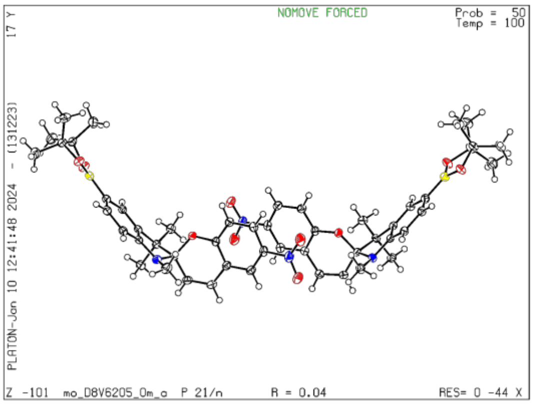

Crystals suitable for single-crystal X-ray diffraction experiments were grown by slow evaporation from a solution of compound **6** in CD_3CN. A colourless, block-shaped crystal of compound **6** was mounted on a MiTeGen micromount with perfluoroether oil. Data were collected from a shock-cooled single crystal at 100(2) K on a Bruker D8 VENTURE dual wavelength Mo/Cu three-circle diffractometer with a microfocus sealed X-ray tube using a mirror optics as monochromator and a Bruker PHOTON III detector. The diffractometer was equipped with an Oxford Cryostream 800 low temperature device and used MoKα radiation (λ = 0.71073 Å). All data were integrated with SAINT and a multi-scan absorption correction using SADABS was applied.[33,34] The structure was solved by direct methods using SHELXT and refined by full-matrix least-squares methods against F^2 by SHELXL-2019/2.[35,36] All non-hydrogen atoms were refined with anisotropic displacement parameters. All hydrogen atoms were refined isotropic on calculated positions using a rigid riding model. Crystallographic data for the structures reported here have been deposited with the Cambridge Crystallographic Data Centre.[37] CCDC 2327468 contain the supplementary crystallographic data for this paper. These data can be obtained free of charge from The Cambridge Crystallographic Data Centre via www.ccdc.cam.ac.uk/structures. This report and the CIF file were generated using FinalCif.[38]

Table S4. Crystal data and structure refinement for compound 6

CCDC number	2327468
Empirical formula	$C_{25}H_{29}BN_2O_5$
Formula weight	448.31
Temperature [K]	100(2)
Crystal system	monoclinic
Space group (number)	$P2_1/n$ (14)
a [Å]	11.6679(13)
b [Å]	35.757(4)
c [Å]	11.7420(12)
α [°]	90
β [°]	102.866(4)
γ [°]	90
Volume [Å^3]	4775.9(9)
Z	8
ρ_{calc} [gcm^{-3}]	1.247
μ [mm^{-1}]	0.086
F(000)	1904
Crystal size [mm^3]	0.186×0.082×0.071
Crystal colour	colourless
Crystal shape	block
Radiation	MoK_α (λ=0.71073 Å)
2θ range [°]	3.76 to 54.21 (0.78 Å)
Index ranges	−14 ≤ h ≤ 14<br>−45 ≤ k ≤ 45<br>−15 ≤ l ≤ 15
Reflections collected	113491
Independent reflections	10533<br>R_{int} = 0.1103<br>R_{sigma} = 0.0615
Completeness to θ = 25.242°	100.0 %
Data / Restraints / Parameters	10533/0/609
Absorption correction T_{min}/T_{max} (method)	0.7195/0.7431 (multi-scan)
Goodness-of-fit on F^2	1.021
Final *R* indexes [$I \geq 2\sigma(I)$]	R_1 = 0.0454<br>wR_2 = 0.1026
Final *R* indexes [all data]	R_1 = 0.0665<br>wR_2 = 0.1171
Largest peak/hole [eÅ^{-3}]	0.27/−0.29

Bibliography

[31] J. Oriou, F. Ng, G. Hadziioannou, G. Garbay, M. Bousquet, L. Vignau, E. Cloutet, C. Brochon, *Polym. Chem*. **2014**, *5*, 7100–7108.

[32] Deposition Number CCDC 2327468 (SP boronic ester; compound **6** in the SI) contains the supplementary crystallographic data for this paper. These data are provided free of charge by the joint Cambridge Crystallographic Data Centre and Fachinformationszentrum Karlsruhe Access Structures service.

[33] Bruker, *SAINT*, *V8.40B*, Bruker AXS Inc., Madison, Wisconsin, USA.

[34] L. Krause, R. Herbst-Irmer, G. M. Sheldrick, D. Stalke, *J. Appl. Cryst.* **2015**, *48*, 3–10, doi:10.1107/S1600576714022985.

[35] G. M. Sheldrick, *Acta Cryst.* **2015**, *A71*, 3–8, doi:10.1107/S2053273314026370.

[36] G. M. Sheldrick, *Acta Cryst.* **2015**, *C71*, 3–8, doi:10.1107/S2053229614024218.

[37] C. R. Groom, I. J. Bruno, M. P. Lightfoot, S. C. Ward, *Acta Cryst.* **2016**, *B72*, 171–179, doi:10.1107/S2052520616003954.

[38] D. Kratzert, *FinalCif*, *V131*, https://dkratzert.de/finalcif.html.
